# Secure Data Access Control for Fog Computing Based on Multi-Authority Attribute-Based Signcryption with Computation Outsourcing and Attribute Revocation

**DOI:** 10.3390/s18051609

**Published:** 2018-05-17

**Authors:** Qian Xu, Chengxiang Tan, Zhijie Fan, Wenye Zhu, Ya Xiao, Fujia Cheng

**Affiliations:** Department of Computer Science and Technology, Tongji University, Cao An Highway, Shanghai 201804, China; jerrytan@tongji.edu.cn (C.T.); 1310898@tongji.edu.cn (Z.F.); 1310513@tongji.edu.cn (W.Z.); 1710053@tongji.edu.cn (Y.X.); 1631585@tongji.edu.cn (F.C.)

**Keywords:** Internet of Things, fog computing, Attribute Based Signcryption, multi-authority, access control, anonymous authentication

## Abstract

Nowadays, fog computing provides computation, storage, and application services to end users in the Internet of Things. One of the major concerns in fog computing systems is how fine-grained access control can be imposed. As a logical combination of attribute-based encryption and attribute-based signature, Attribute-based Signcryption (ABSC) can provide confidentiality and anonymous authentication for sensitive data and is more efficient than traditional “encrypt-then-sign” or “sign-then-encrypt” strategy. Thus, ABSC is suitable for fine-grained access control in a semi-trusted cloud environment and is gaining more and more attention recently. However, in many existing ABSC systems, the computation cost required for the end users in signcryption and designcryption is linear with the complexity of signing and encryption access policy. Moreover, only a single authority that is responsible for attribute management and key generation exists in the previous proposed ABSC schemes, whereas in reality, mostly, different authorities monitor different attributes of the user. In this paper, we propose OMDAC-ABSC, a novel data access control scheme based on Ciphertext-Policy ABSC, to provide data confidentiality, fine-grained control, and anonymous authentication in a multi-authority fog computing system. The signcryption and designcryption overhead for the user is significantly reduced by outsourcing the undesirable computation operations to fog nodes. The proposed scheme is proven to be secure in the standard model and can provide attribute revocation and public verifiability. The security analysis, asymptotic complexity comparison, and implementation results indicate that our construction can balance the security goals with practical efficiency in computation.

## 1. Introduction

With the rapid development of cloud computing, more people are coming to prefer moving both the large burden of data storage and computation overhead to cloud servers in a cost-effective manner [1]. However, the advance of the Internet of Things (IoTs) has posed a challenge to the centralized cloud computing system due to its geo-distribution, location awareness, and low latency requirements. To solve the problem, Cisco proposed the concept of fog computing in 2014, where a layer consisting of fog devices (such as routers, access points, and IP video cameras) bridges between the cloud server and end users [2]. In a fog computing system, the fog devices, termed as fog nodes, are distributed and implemented at the edge of networks [3]. Since fog nodes are much closer to end users than the cloud server and have plentiful computing resources and wireless communication facility, some of the computing tasks can be outsourced to fog nodes from the nearby end user, which alleviates the computation burden of the users and significantly improve the efficiency. Thus, the fog computing paradigm can be applied in many real-time and geographically distributed applications, such as wireless sensors, smart grids and health fog applications [4].

However, there are still various challenging obstacles in fog computing systems, such as the privacy and security of users’ data [5,6]. Traditionally, a cloud server is not fully trusted by the data owner in cloud computing system, and the data uploaded may contain sensitive information; hence, the data should be encrypted before outsourcing to the cloud. In accord with cloud computing, message confidentiality should also be considered in fog computing systems. Moreover, since the fog nodes are more easily compromised than cloud servers [6], it is required that fog nodes should alleviate the computation burden of end devices without degrading the privacy in fog computing systems. In addition to confidentiality, data owners may wish to impose fine-grained access control such that only users with certain attributes have access to the data [7]. For example, in a health fog system, which combines the advantage of both the fog computing and original cloud-based healthcare services [8], personal health records usually contain abundant sensitive information, such as weight, heart rate, and blood type. After gathering by sensors, the personal health record may be uploaded to the cloud for the user’s individual needs or to perform real-time analytics. To ensure the privacy of the health data, an access control system should guarantee that only the users authorized by the data owner can access the data. For instance, to analyze whether the blood pressure is normal, the owner “Alice” wants to share her health data to users with attributes “*Institution* = *Hospital* ∧ *Role* = *Doctor* ∧ *Gender* = *Female*”. One of the effective techniques to address this fine-grained access requirement is attribute-based encryption (ABE) [9]. It realizes the confidentiality and access control on data based on encryption under an access policy defined over the set of attributes.

Besides the confidentiality and fine-grained access control, it is also necessary to provide anonymity authentication for data sharing between users in the access control mechanism. For instance, the owner “Alice”, aged 20, would like to encrypt and store some sensitive health information in the cloud but does not want to be recognized. When a data user, such as the doctor or researcher, accesses the data, he/she can verify that the data is actually uploaded by a patient with certain credentials such as “*Gender* = *Female* ∧ *Age* ∈ [18,30]” without knowing the patient’s real identity “Alice” or her real age.

A feasible and promising solution is the Attribute Based Signcryption (ABSC) scheme, which takes advantages of Attribute-Based Encryption (ABE) and Attribute-Based Signature (ABS), and is more efficient than do the traditional “encrypt-then-sign” or “sign-then-encrypt” strategies. ABSC employs ABE to provide confidentiality and fine-grained access control, and uses ABS to achieve authentication without revealing the data owner’s sensitive attributes. Traditionally, ABE can be classified into two categories: Key-Policy ABE (KP-ABE) and Ciphertext-Policy ABE (CP-ABE). In KP-ABE, the secret key is associated with an access structure (predicate), and the message is encrypted with a set of attributes. While in CP-ABE, predicate is assigned to the plaintext message. Similarly, ABS has two categories: Signature-Policy ABS (SP-ABS) wherein the predicate is embedded in the signature, and Key-Policy ABS (KP-ABS) wherein the predicate is associated with the secret key. The Ciphertext-Policy ABSC (CP-ABSC) [10] supports CP-ABE and SP-ABS, and the Key-Policy ABSC (KP-ABSC) [11] supports KP-ABE and KP-ABS. Recently, many data access control schemes based on ABSC have been proposed, as in [12,13,14,15]. Although some of them are efficient, three problems must be considered when implementing ABSC scheme in fog computing environment. The first one is performance. The traditional ABSC scheme is typically computationally intensive. In particular, the cost of signcryption and designcryption on the user side are proportional to the complexity of predicates. One possible strategy to alleviate the computation overhead required on end user is to outsource the most computation-consuming job of signcryption and designcryption to the fog node. Although many ABE schemes with outsourcing encryption and decryption, as in [16,17,18,19,20], have been proposed in recent years for secure data sharing in fog computing system, realizing ABSC scheme with anonymous authentication and efficient computation outsourcing is still a challenge since ABSC schemes contain both of the signing and encryption protocols. The second problem is multi-authority. In traditional ABSC schemes, as in [12,13,14,15], a central authority is responsible for attribute management and key generation. However, in many applications, the predicate embedded in the ciphertext or signature can be written over attributes issued by different trust domains and authorities. For example, the health data uploaded by “Alice” may contain the encryption predicate as “(*Doctor* ∨ *Researcher*) ∨ *Female*”. Since only a hospital can authorize a person the attribute “*Doctor*” and only a research organization can certify that a person is a “*Researcher*”, it is not practical to authorize access right to a person by a single authority. Therefore, it is necessary to distribute attribute management and secret key generation from a single central authority over many authorities. Some multi-authority ABE schemes for fog computing, as in [17], have been proposed, whereas constructing multi-authority ABSC scheme with outsourcing capability is still a blank. The third one is attribute revocation. For example, when the attributes of a doctor are updated from *A* = {*Institution* = *Hospital* ∧ *Role* = *Doctor* ∧ *Gender* = *Female*} to *B* = {*Institution* = *Hospital* ∧ *Gender* = *Female*}, her access rights should be modified accordingly. Attribute revocation is not trivial and straightforward in ABE schemes. However, it has not been taken into account in multi-authority ABSC schemes with outsourcing capability.

The problem of designing a multi-authority data access control scheme based on ABSC with signcryption and designcryption outsourcing capabilities and attribute revocation for fog computing system, has received very little attention so far, although some schemes based on Multi-Authority ABE (MA-ABE) and ABS (MA-ABS) for cloud storage setting have been proposed, as in [21,22,23,24,25,26]. Meng et al. [27] proposed a decentralized KP-ABSC scheme for secure data sharing in the cloud. However, the scheme is just a combination of identity signature and MA-ABE, and only supports the threshold predicate. It also does not provide any security definition or computation outsourcing. Hong et al. [28] proposed a KP-ABSC scheme with outsourced designcryption and key exposure protection. However, the computation overhead of signcryption increases with the complexity of the predicate, and since the verification and decryption both have to be performed on the user side, the number of pairing operations evaluated on the user side is proportional to the sum of the required attributes, which is not acceptable to IoT devices. Moreover, the scheme in [28] does not support multi authorities and attribute revocation. We focus on CP-ABSC in access control application, as CP primitives are more suitable for the data owner to choose the predicate to determine who can access the sensitive data [14].

### 1.1. Contributions

In this paper, we propose OMDAC-ABSC, a novel data access control scheme for fog computing system based on Multi-Authority CP-ABSC (MACP-ABSC) supporting the computation outsourcing for both signcryptor (data owner) and designcryptor (data user). To the best of our knowledge, OMDAC-ABSC is the first scheme that significantly reduces computation burden from both data owners and data users in the multi-authority ABSC setting. Public verifiability, expressiveness and attribute revocation are also considered in our scheme. The main contributions can be summarized as follows:

(1) We propose a data access control scheme OMDAC-ABSC for fog computing system, in which fog nodes serve as a bridge between the cloud server and end users. In our scheme, heavy signcryption and designcryption operations can be outsourced from end users (e.g., tablet computers and smartphones) to fog nodes. In signcryption phase, the fog nodes are in charge of generating part of the ciphertext. In designcryption phase, the fog nodes can perform the partial decryption without degrading the data confidentiality, and the data user only requires a constant number of exponentiations to decrypt the ciphertext. Additionally, unlike other existing works such as [27,28], our scheme supports public verification, since the verification mechanism does not require the plaintext message or the data owner’s public key. Thus the verification algorithm can be performed by any trusted party, which alleviates the computation burden of the end user. Therefore, our construction is efficient from computation point of view.
(2) Unlike some existing ABE schemes for fog computing such as [16,18,19] and ABSC schemes such as [15,27,28], the proposed OMDAC-ABSC scheme is more expressiveness and supports any monotone Boolean function predicates represented by monotone span programs (MSP) for both signing and encryption. Moreover, we remove the limitation that the labeling functions *ρ* in signing and encryption predicates should be injective functions.
(3) Our OMDAC-ABSC scheme is proven to be secure in the standard model. We also formally prove that our construction satisfies the properties of signcryptor privacy and collusion resistance.
(4) We also consider the attribute revocation in our OMDAC-ABSC scheme. In attribute revocation phase, the authority supervising the revoked attribute only distributes the update keys to the non-revoked users and the cloud server to update the corresponding components. It is also proved that our scheme guarantees both the forward and backward revocation security.

### 1.2. Paper Organization

The remainder of this paper is organized as follows: in Section 2, we discuss some related works. Then in Section 3, we review the necessary notations and cryptographic background that are used throughout the paper. In Section 4, we give the definition of our scheme and the security requirements. The details of the scheme and the security proof are elaborated in Section 5 and Section 6, respectively. Section 7 is dedicated to discussing the functionality and performance of the scheme. Finally, we conclude this paper in Section 8.

## 2. Related Works

### 2.1. Access Control Schemes Based on ABE

ABE was first introduced by Sahai and Waters [9]. In ABE, a data owner can share sensitive data with others according to predicates (or access policies). Several works on ABE have been presented to address data access control in untrusted cloud servers. Recently, the ABE scheme was adopted in fog-computing systems to guarantee confidentiality and fine-grained access control. Heavy computations of encryption or decryption are outsourced to fog nodes to improve the efficiency. In [16], an anonymous user authentication in ciphertext update phase was realized, whereas the scheme only supports AND-gate predicate. Zuo et al. [18] proposed a CCA-secure ABE scheme with decryption outsourcing. However, the encryption phase of the scheme in [18] incurs heavy computation cost. Additionally, the scheme in [18] is only provably secure in the random oracle model and only supports the AND-gate encryption predicate. Zhang et al. [19] presented an ABE- based access control scheme for fog computing with outsourced encryption and decryption. Although the computation operations (pairings and exponentiations) for users to encrypt and decrypt are irrelevant to the complexity of predicate, the scheme only supports threshold encryption predicate, and requires both the cloud server and fog nodes to be trusted. Lounis et al. [29] proposed a cloud-based architecture for medical wireless sensor networks, in which the resource-constrained end devices outsource the costly computations to the trusted gateway. However, the decryption phase incurs heavy computation cost. Xiao et al. [30] constructed a fine-grained hybrid scheme for fog computing with the advantages of efficient data search and access authorization through online/offline encryption, delegation of search task and decryption to fog nodes, and provable security. Mao et al. [20] proposed an ABE scheme with verifiable outsourced decryption, whereas it incurs a heavy computation overhead in encryption phase. Li et al. [31] also proposed a fully verifiable ABE scheme with outsourcing capability. However, Liao et al. [32] showed that the verification mechanism proposed in [31] is not always correct.

In many ABE schemes, the attribute universe is assumed to be managed by a single authority. In reality, however, users’ attributes may be monitored by different authorities. To track this problem, MA-ABE scheme was proposed by Chase et al. [33]. In MA-ABE, the attribute universe is divided into multiple disjoint sets, and each authority controls one of these attribute sets. The user can successfully decrypt the ciphertext if and only if the user possesses at least a pre-specified number of attributes from each authority. Furthermore, Chase et al. [34] proposed an improved MA-ABE scheme to remove the fully trusted central authority by adopting a Pseudo Random Function (PRF) and a secure 2-party anonymous secret-key-issuing protocol. However, the multiple authorities must cooperate with each other, and the number of authorities must be determined in the initialization phase. Recently, many distributed access control schemes based on MA-ABE have been proposed, such as [21,22,23,24,25,26,35,36]. Han et al. [21] proposed a privacy-preserving decentralized CP-ABE based access scheme (PPDCP-ABE) to protect the user’s privacy. However, PPDCP-ABE cannot resist collusion attack or support anonymous authentication. Rui et al. [22] constructed a MA-ABE scheme with secure attribute-level immediate attribute revocation. The scheme is only provably secure under the random oracle model. Lewko et al. [23] proposed a decentralized attribute-based encryption using the dual system encryption methodology. The secret keys of the user are tied to his global identity in order to resist collusion attack. However, the scheme realizes the security in random oracle model using the composite-order bilinear group, which incurs great computation overhead. Sourya et al. [25] proposed a decentralized data sharing scheme with outsourced decryption and user revocation. They also proposed a decentralized data sharing scheme where multiple attribute authorities distribute secret keys to the user [24]. In [26], the authors outsourced the main computation overhead in a decryption algorithm to the cloud. However, the security cannot be guaranteed if the revoked user eavesdrops to obtain the update keys and retrieves the ability to decrypt as a non-revoked user. To implement multi-authority ABE in fog computing system, Fan et al. [17] proposed a VO-MAACS scheme with verification mechanism. Although the encryption and decryption algorithms are outsourced, the scheme cannot support anonymous authentication and attribute revocation, and does not have security proof. Jung et al. [35] presented an anonymous privilege control scheme to address data and identity privacy in multi-authority cloud storage system. To guarantee the confidentiality of user’s identity information, the scheme in [35] decomposes the central authority to multiple ones while preserving tolerance to compromise attack on the authorities. However, the security is realized in random oracle model, and the encryption predicate is the AND gate. In [36], the authors constructed a multi-authority data access control scheme with decryption outsourcing and attribute-level user revocation. The scheme supports any monotone encryption predicate and is adaptively secure in the standard model. Nevertheless, the scheme in [36] needs to deal with large composite-order group elements and thus incurs heavy computation overhead.

### 2.2. Attribute-Based Signature and Multi-Authority Attribute-Based Signature

ABS was first introduced by Maji et al. [37]. Due to their anonymity and authentication properties, many ABS schemes have been proposed. Like ABE, to overcome the drawback that only a single authority exists in the system, the concept of MA-ABS was introduced in [38]. In MA-ABS, there are multiple authorities and each authority controls one of disjoint attribute sets. The user is able to successfully sign the plaintext if he/she possesses a pre-specified number of attributes from multiple authorities.

### 2.3. Access Control Schemes Based on ABSC

ABSC scheme, first introduced by Gagné et al. [10], is a logical combination of ABE and ABS and can support many practical properties, including confidentiality, fine-grained access control, and authentication. Recently, many data access control schemes based on ABSC have been proposed, as in [11,12,13,14,15,27,28]. Y. Sreenivasa [11] proposed a Key-Policy attribute-based signcryption scheme that supports any monotone Boolean function and constant size ciphertext. However, the message confidentiality and unforgeability of the scheme against selectively adversary are proven in the random oracle model. Chen et al. [12] focused on the joint security of signature and encryption schemes and presented a CP-ABSC scheme in the joint security setting. However, it cannot support public verifiability since plaintext is required in verification mechanism. Liu et al. [13] proposed a secure PHR data access control scheme based on CP-ABE [39] and ABS [37]. However, it is only provably secure in a random oracle model. In [14], the authors constructed a CP-ABSC based access control scheme with public verifiability, but the scheme does not support computation outsourcing. Yu et al. [15] proposed the hybrid access policy ABSC scheme that supports KP-ABS and CP-ABE. The size of the ciphertext is constant, and the scheme realizes security in the standard model. Nevertheless, it only supports the threshold predicate in the encryption phase. Moreover, the above ABSC schemes only have a single authority and cannot be applied in the multi-authority system.

## 3. Preliminaries

By $a\overset{R}{\leftarrow }A$, we denote that a is selected randomly from A. |A| denotes the cardinality of a finite set A. ${\mathbb{Z}}_{p}$ denotes a finite field with prime order p, and ${\mathbb{Z}}_{p}^{*}$ stands for ${\mathbb{Z}}_{p}\backslash \left\{0\right\}$. y←A(x) denotes that y is computed by running algorithm A with input x. [n] represents the set {1,2,…,n}. ${\vec{a}}^{\left(i\right)}$ denotes the ith element of the vector $\vec{a}$. A function ϵ: ℤ→R is negligible if, for any z∈ℤ, there exists a k such that $\epsilon \left(x\right)<1/x^{z}$ when x>k. We use s and e as superscripts for signing and encryption, respectively. Pr[E] denotes the probability of an event E occurring. For an unambiguous presentation of the paper, we define the important notations used in our scheme in the Appendix A.

**Definition** **1.** *Bilinear maps [22]: Let G and $G_{T}$ be two cyclic groups with the prime order p, and g∈G be the generator of G. Then the bilinear map $e:G\times G\to G_{T}$ can be defined as follows:*

- *Bilinear. For all u,v∈G, $a,b\in {\mathbb{Z}}_{p}$, $e\left(u^{a},v^{b}\right)=e{\left(u,v\right)}^{ab}$.*
- *Non-degenerate. e(g,g)≠1.*
- *Computable. There is an efficient algorithm to compute the map e.*

$GG\left(1^{k}\right)\to \left(e,p,G,G_{T}\right)$ takes as input a security parameter $1^{k}$ and outputs a bilinear group $\left(e,p,G,G_{T}\right)$ with prime order p and a bilinear map $e:G\times G\to G_{T}$.

**Definition** **2.** Decisional Bilinear Diffie-Hellman (BDH) Assumption [22]: Let g be a generator of G with prime order p and $a,b,c\in {\mathbb{Z}}_{p}^{*}$ be randomly chosen. Given a vector $\vec{Y}=\left(g,g^{a},g^{b},g^{c}\right)$, the decisional BDH assumption holds if no PPT adversary A can distinguish $\left(\vec{Y},\Omega =e{\left(g,g\right)}^{abc}\right)$ from $\left(\vec{Y},\Omega \overset{R}{\leftarrow }G_{T}\right)$ with the advantage $Adv_{A}=\left|Pr\left[A\left(\vec{Y},\Omega =e{\left(g,g\right)}^{abc}\right)=1\right]-Pr\left[A\left(\vec{Y},\Omega \overset{R}{\leftarrow }G_{T}\right)=1\right]\right|\geq \epsilon \left(k\right)$.

**Definition** **3.** *Decisional q-Parallel Bilinear Diffie-Hellman Exponent (q-PBDHE) Assumption [21]: Suppose that $a,w,b_{1},b_{2},\ldots ,b_{q}\overset{R}{\leftarrow }{\mathbb{Z}}_{p}$, $GG\left(1^{k}\right)\to \left(e,p,G,G_{T}\right)$ and g is a generator of G. Given $\vec{Y}=\left(g,g^{w},g^{a},\ldots ,g^{a^{q}},g^{a^{q+2}},\ldots ,g^{a^{2q}},\forall 1\leq j\leq q,g^{wb_{j}},g^{\frac{a}{b_{j}}},\ldots ,g^{\frac{a^{q}}{b_{j}}},g^{\frac{a^{q+2}}{b_{j}}}\ldots ,g^{\frac{a^{2q}}{b_{j}}},\forall 1\leq j,k\leq q,k\neq j,g^{\frac{awb_{k}}{b_{j}}},\ldots ,g^{\frac{a^{q}wb_{k}}{b_{j}}}\right)$, the decisional q-PBDHE assumption holds if no PPT adversary A can distinguish $\left(\vec{Y},\Omega =e{\left(g,g\right)}^{a^{q+1}w}\right)$ from $\left(\vec{Y},\Omega \overset{R}{\leftarrow }G_{T}\right)$ with the advantage $Adv_{A}=\left|Pr\left[A\left(\vec{Y},\Omega =e{\left(g,g\right)}^{a^{q+1}w}\right)=1\right]-Pr\left[A\left(\vec{Y},\Omega \overset{R}{\leftarrow }G_{T}\right)=1\right]\right|\geq \epsilon \left(k\right)$.*

**Definition** **4.** *Monotone Span Program (MSP) [11]: Assume $\left\{v_{1},v_{2},\ldots ,v_{m}\right\}$ is a set of variables. An MSP is a labeled matrix $\Omega ≔\left(M_{\ell \times n},\rho \right)$, where M is an ℓ×n matrix over ${\mathbb{Z}}_{p}$ and ρ is the labeling function $\rho :\left[\ell \right]\to \left\{v_{1},v_{2},\ldots ,v_{m}\right\}$.*

*Let $\vec{x}=\left(x_{1},x_{2},\ldots ,x_{m}\right)\in {\left\{0,1\right\}}^{m}$ and $X_{\mu }=\left\{i\in \left[\ell \right]:\left[\rho \left(i\right)=v_{j}\right]\wedge \left[x_{j}=\mu \right]\right\}$ where μ∈{0,1}. $X_{1}\cup X_{0}=\left[\ell \right]$. Let $M^{i}$ be the ith row of M. We denote $\Omega \left(\vec{x}\right)=1$ if Ω accepts the input $\vec{x}$. Likewise, $\Omega \left(\vec{x}\right)=0$ means Ω rejects $\vec{x}$. Then $\Omega \left(\vec{x}\right)=1\Leftrightarrow \left[\exists \left(a_{1},a_{2},\ldots ,a_{\ell }\right)\in {\mathbb{Z}}_{p}^{\ell }\;such\;that\;\sum_{i\in \left[\ell \right]}a_{i}M^{i}=\vec{1}\right]$ where $a_{i}=0$ for all $i\in X_{0}$.*

*An MSP Ω computes a monotone Boolean function $R:\;{\left\{0,1\right\}}^{m}\to \left\{0,1\right\}$ if $\Omega \left(\vec{x}\right)=1$ for all $\vec{x}\in \left\{\vec{x}:\;R\left(\vec{x}\right)=1\right\}$.*

**Lemma** **1** **[14].** *If $\Omega \left(\vec{x}\right)=0$, then there exists a vector $\vec{\omega }=\left(\omega_{1},\omega_{2},\ldots ,\omega_{n}\right)\in {\mathbb{Z}}_{p}^{n}$ with $\omega_{1}=-1$ such that $\vec{\omega }M^{i}=0$ for all $i\in X_{1}$.*

**Definition** **5.** *Predicates [14]: Assume U is the universe of attributes. A predicate over U is a monotone Boolean function whose inputs are associated with the attributes of U. Let W⊂U is a subset of attributes. A predicate R accepts W⊂U if R(W)=1. If W does not satisfy R then R(W)=0. A predicate R is said to be monotone, if R(W)=1⇒R(C)=1 for every attribute set C⊃W.*

*Suppose R is a predicate and $L_{R}$ is the set of attributes utilized in R. Then the corresponding MSP for R is a labeled matrix $\Omega ≔\left(M_{\ell \times n},\rho \right)$, where $\rho :\left[\ell \right]\to L_{R}$.*

*Define $X_{1}=\left\{i\in \left[\ell \right]:\left[\rho \left(i\right)=a\right]\wedge \left[a\in W\right]\right\}$ and $X_{0}=\left\{i\in \left[\ell \right]:\left[\rho \left(i\right)=a\right]\wedge \left[a\notin W\right]\right\}$. $X_{1}\cup X_{0}=\left[\ell \right]$. Then*

*$R\left(W\right)=1\Leftrightarrow \Omega \left(W\right)=1\Leftrightarrow \left[\exists \left(a_{1},a_{2},\ldots ,a_{\ell }\right)\in {\mathbb{Z}}_{p}^{\ell }\;such\;that\;\sum_{i\in \left[\ell \right]}a_{i}M^{i}=\vec{1}\;and\;a_{i}=0\;\forall i,\rho \left(i\right)\notin W\;\right]$.*

**Lemma** **2** **[14].** *If R(W)=0, then there exists a vector $\vec{\omega }=\left(\omega_{1},\omega_{2},\ldots ,\omega_{n}\right)\in {\mathbb{Z}}_{p}^{n}$ with $\omega_{1}=-1$ such that $\vec{\omega }M^{i}=0$ for all i where ρ(i)∈W.*

**Definition** **6** **[14].** *Let $M_{\ell \times n}$ be a matrix of size ℓ×n over a field F. rank(M) is rank of $M_{\ell \times n}$. If rank(M)<ℓ, then $V=\left\{\left(b_{1},b_{2},\ldots ,b_{\ell }\right)\in F^{\ell }:\sum_{i\in \left[\ell \right]}b_{i}M^{i}=\vec{0}\right\}$ contains a polynomial number of vectors $\left(b_{1},b_{2},\ldots ,b_{\ell }\right)$, and the predicate for which MSP is $\Omega ≔\left(M_{\ell \times n},\rho \right)$ consists of both AND and OR gates. Otherwise, $V=\left\{\vec{0}\right\}$ and the predicate is an AND gate. In our construction, we consider the signing and encryption predicates consisting of both AND and OR gates.*

## 4. Scheme and Security Definitions

Our OMDAC-ABSC scheme consists of a multi-authority attribute-based signcryption (MACP-ABSC) scheme.

### 4.1. Multi-Authority Attribute-Based Signcryption

The MACP-ABSC scheme consists of the following five algorithms:

$GlobalSetup\;\left(1^{k}\right)$. Taking as input a security parameter $1^{k}$, the algorithm outputs the public parameters PP. It also generates the public key $PK_{uid}$ for the user with identity uid.

AuthoritySetup(PP). It takes as input PP and outputs the public key and secret key pairs {PK,SK} for the authority.

$SecretKeyGen\left(PP,PK_{aid},SK_{aid},PK_{uid},\tilde{U}\right)$. Taking as input PP, $\left\{PK_{aid},SK_{aid}\right\}$ of authority $AA_{aid}$, user’s public key $PK_{uid}$ and attribute set $\tilde{U}=\tilde{U^{d}}\cup \tilde{U^{s}}$, where $\tilde{U^{d}}$ denotes the set of decryption attributes, and $\tilde{U^{s}}$ is the set of signing attributes. $\tilde{U^{d}}\cap \tilde{U^{s}}=\emptyset .$ The algorithm outputs the secret signing and decryption keys $SK_{uid,aid}=\left\{SK_{uid,aid}^{s},SK_{uid,aid}^{d}\right\}$ for the user.

$Signcryption\left(M,PP,R_{s},R_{e},{\left\{SK_{do,k}^{s}\right\}}_{k\in I}\right)$. Taking as input the plaintext M, public parameters PP, signing and encryption predicates $R_{s},R_{e}$, and the set of signcryptor’s secret signing keys ${\left\{SK_{do,k}^{s}\right\}}_{k\in I}$, where I is the set of involved authorities in signcryption and do is signcryptor’s identity. The algorithm outputs the ciphertext CT.

$DeSigncryption\left(PP,CT,PK_{du},{\left\{SK_{uid,k}^{d}\right\}}_{k\in I}\right)$. This algorithm intakes the public parameters PP, ciphertext CT, public key $PK_{du}$ of the data user $U_{du}$ (designcryptor), and the set of designcryptor’s secret decryption keys ${\left\{SK_{uid,k}^{d}\right\}}_{k\in I}$, outputs the plaintext M or ⊥.

**Definition** **7.** *Assume the signcryptor is denoted by $U_{do}$ and designcryptor is denoted by $U_{du}$. We say that the MACP-ABSC scheme is correct if $R_{s}\left(\tilde{U_{du}^{s}}\right)=1,R_{e}\left(\tilde{U_{du}^{d}}\right)=1$, then $Pr\left[M\leftarrow DeSigncryption\left(PP,CT,PK_{du},{\left\{SK_{du,k}^{d}\right\}}_{k\in I}\right)\right]=1$, where $\left\{PP,PK_{do}PK_{du}\right\}\leftarrow GlobalSetup\left(1^{k}\right)$, $\left\{PK_{k},SK_{k}\right\}\leftarrow AuthoritySetup\left(PP\right)$, $SK_{do,k}^{s}\leftarrow SecretKeyGen\left(PP,PK_{k},SK_{k},PK_{do},\tilde{U_{do}}\right)$, $SK_{du,k}^{d}\leftarrow SecretKeyGen\left(PP,PK_{k},SK_{k},PK_{du},\tilde{U_{du}}\right)$, $CT\leftarrow Signcryption\left(M,PP,R_{s},R_{e},{\left\{SK_{do,k}^{s}\right\}}_{k\in I}\right)$.*

### 4.2. High-Level Overview of OMDAC-ABSC Scheme

Based on MACP-ABSC scheme, we propose OMDAC-ABSC scheme, a novel data access control scheme for fog computing system supporting the computation outsourcing for both signcryptor and designcryptor.

#### 4.2.1. Scheme Description

**Figure 1:**
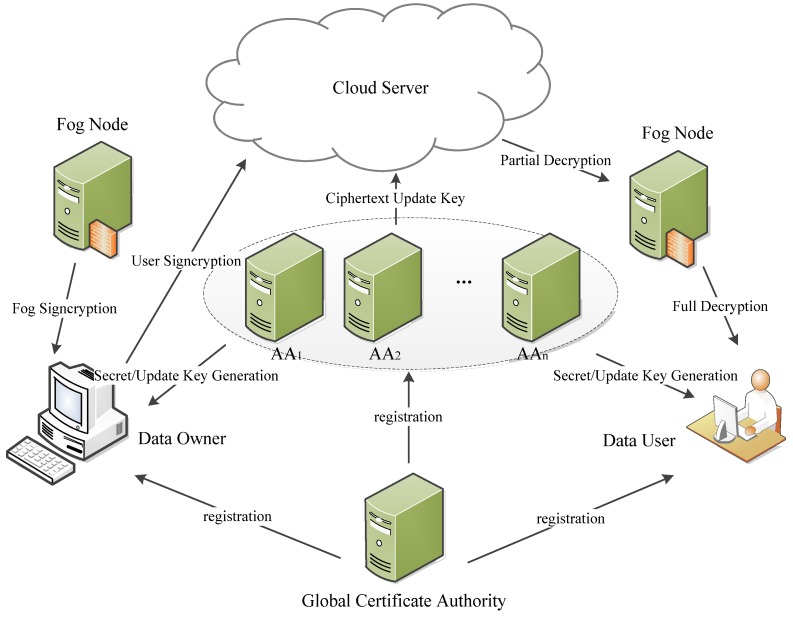
System Architecture.

As shown in Figure 1, our OMDAC-ABSC scheme has five types of entities: the global certificate authority (CA), cloud server, users (including signcryptors and designcryptors), independent attribute authorities (AAs) and fog nodes.

*Global Certificate Authority*: The global certificate authority (CA) is fully trusted in the system and generates the public parameters for the system. CA is also responsible for the users’ and authorities’ registrations. However, CA is not involved in any attribute management and the creations of the secret keys that are associated with attributes. With the help of CA, we can improve the privacy of our scheme by realizing the identity authentication and preventing authorities from forging a virtual user to decrypt the ciphertext. In secret key generation phase, the attribute authority verifies user’s certification using the verification key of CA and then generates the secret key for the user. In designcryption phase, the cloud server can verify user’s identifier and return the ciphertext to the fog node if the user is valid.

*Cloud Server*: The cloud server is a semi-trusted party and also provides data storage and data access service to users. Since our scheme supports public verification, the cloud server can verify that the ciphertext is valid and is signcrypted by the data owner whose attributes satisfy the signing predicates contained in the ciphertext. If the ciphertext is not valid, the cloud server can reject it.

*User*: Users who are attached to fog nodes and equipped with IoT devices in our system include the signcryptor and designcryptor. When the signcryptor signcrypts a message, he/she can select the signing and encryption predicates over the attributes from multiple authorities and outsource the resulting ciphertext to the cloud server. We assume that the ciphertext implicitly contains the signing and encryption predicates. Only legally registered users can endorse the data, and only users satisfying the encryption predicate can decrypt the data.

*Attribute Authority*: The authority can initialize itself to setup its public and secret keys. To compute the secret keys for users, the authority verifies the user’s identity and generates the secret keys according to the user’s attributes.

*Fog Node*: Fog nodes, deployed at the edge of the network, offer a variety of services, such as low latency, location awareness, and real-time applications. Each of them is linked to the cloud server. Fog nodes are also in charge of part of signcryption and designcryption computations. Note that in designcryption phase, only if the data user’s attributes satisfy the encryption predicate will the fog nodes partially designcrypt the ciphertext with the proxy secret keys.

**Figure 2:**
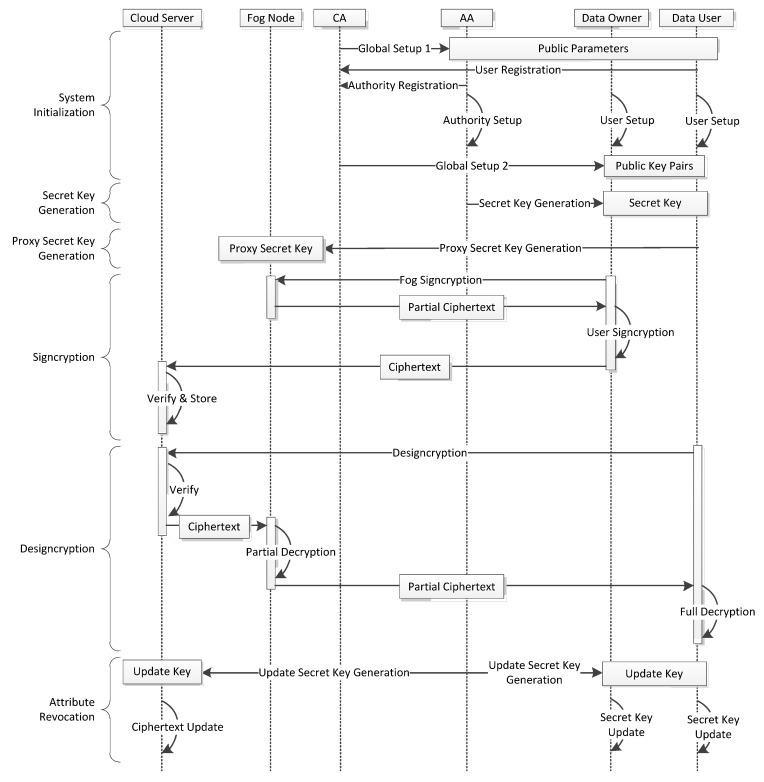
Work flow of OMDAC-ABSC scheme.

The work flow of OMDAC-ABSC scheme is shown in Figure 2. The scheme consists of the following six phases.

(1) System Initialization

In this phase, CA generates the public parameters for the system, and also accepts the registrations of the attribute authorities and the users. The initialization phase contains the following six algorithms:

$GlobalSetup1\left(1^{k}\right)$. This algorithm is run by CA. Taking as input the security parameter $1^{k}$, the algorithm outputs the public parameters PP.

UserReg(PP). This algorithm is run by CA and data user. Taking as input the public parameters, CA assigns the global identity uid and partial public key $PPK_{uid}$ to the user.

AuthorityReg(PP). This algorithm is run by CA and the attribute authority. Taking as input the public parameters, CA assigns the global identity aid and partial public key $PPK_{aid}$ for the attribute authority.

$UserSetup\left(PP,PPK_{uid}\right)$. Given the global identity uid, public parameters PP, and partial public key $PPK_{uid}$, the data user runs $UserSetup\left(PP,PPK_{uid}\right)$ to initialize himself/herself. The algorithm outputs the public key $PK_{uid}$ and secret key $SK_{uid}$ for the user. Additionally, the public key certificate cert(uid) generated by CA is sent to the user for identity authentication.

$AuthoritySetup\left(PP,PPK_{aid}\right)$. Given the global identity aid, public parameters PP, and partial public key $PPK_{aid}$, the attribute authority runs $AuthoritySetup\left(PP,PPK_{aid}\right)$ to initialize itself. The algorithm outputs the public key $PK_{aid\;},PK_{uid,aid}^{1}$ and secret key $SK_{aid\;}$ for the attribute authority $AA_{aid}$.

$GlobalSetup2\left(1^{k},PP,\;{\left\{PK_{aid},PK_{uid,aid}^{1}\right\}}_{U_{uid}\in S_{U},AA_{aid}\in S_{A}}\right)$. This algorithm is run by CA to end the system initialization phase. Taking as input the public parameters PP and authorities’ public keys ${\left\{PK_{aid},PK_{uid,aid}^{1}\right\}}_{U_{uid}\in S_{U},AA_{aid}\in S_{A}}$, CA generates the public key $PK_{uid,aid}$ for each pair of user $U_{uid}$ and authority $AA_{aid}$.

(2) Secret Key Generation

After system initialization, the attribute authority $AA_{aid}$ can verify the user’s identity using the public key certificate cert(uid) and then run $SecretKeyGen\left(PP,PK_{aid},SK_{aid},PK_{uid},\tilde{U}\right)$ algorithm to compute the secret signing and decryption keys for the valid user according to the user’s attribute set $\tilde{U}$.

$SecretKeyGen\left(PP,PK_{aid},SK_{aid},PK_{uid},\tilde{U}\right)$. The algorithm intakes the public parameters PP, the public key and secret key pair $\left\{PK_{aid},SK_{aid}\right\}$ of the authority $AA_{aid}$, the public key $PK_{uid}$ and user’s attribute set $\tilde{U}$, outputs the user’s secret signing and decryption keys $SK_{uid,aid}=\left\{SK_{uid,aid}^{s},SK_{uid,aid}^{d}\right\}$.

(3) Proxy Secret Key Generation

In this phase, the data user runs $PxSecretKeyGen\left(SK_{uid},SK_{uid,aid}\right)$ algorithm to compute the proxy secret signing and decryption keys $PSK_{uid,aid}=\left\{PSK_{uid,aid}^{s},PSK_{uid,aid}^{d}\right\}$ and then sends $PSK_{uid,aid}$ to the fog nodes to outsource the signcryption and designcryption computation overhead.

$PxSecretKeyGen\left(SK_{uid},SK_{uid,aid}\right)$. Taking as input the secret key $SK_{uid}$ and secret signing and decryption keys $SK_{uid,aid}$, this algorithm outputs the proxy secret signing and decryption keys $PSK_{uid,aid}=\left\{PSK_{uid,aid}^{s},PSK_{uid,aid}^{d}\right\}$. $PSK_{uid,aid}$ are sent to the fog nodes.

(4) Data Signcryption

To achieve high efficiency, the signcryptor first encrypts the plaintext with a random content key by applying a symmetric encryption algorithm. Then the signcryptor defines the signing and encryption predicates $R_{s}$ and $R_{e}$, and signcrypts the content secret key with the following two algorithms:

$Fog\_Signcryption\left(PP,{\left\{PSK_{uid,k}^{s}\right\}}_{k\in I_{A}^{s}},PK_{uid},R_{s},R_{e}\right)$. This algorithm is performed in the fog nodes. Taking as input the public parameters PP, proxy secret signing key $PSK_{uid,k}^{s}$ of the attribute authority $AA_{k}$ whose attributes are selected for signing, the public key $PK_{uid}$ of signcryptor, the signing and encryption predicates $R_{s},R_{e}$, the algorithm outputs part of the ciphertext $CT^{\prime }$.

$User\_Signcryption\left(M,PP,{\left\{PK_{aid}\right\}}_{aid\in I_{A}^{e}},SK_{uid},CT^{\prime }\right)$. This algorithm intakes the message to be signcrypted, the public parameters PP, the public key $PK_{aid}$ of attribute authorities whose attributes are selected for encryption, secret key $SK_{uid}$ of signcryptor and partial ciphertext $CT^{\prime }$, outputs the ciphertext CT and sends CT to the cloud server.

(5) Data Designcryption

When the user queries the ciphertext, the cloud server verifies the user’s identifier and returns the ciphertext to the fog node if the user is valid. If the decryption attribute set $\tilde{U^{d}}$ satisfies the encryption predicate $R_{e}$ embedded in ciphertext, the data user can obtain the plaintext by running $DeSigncryption\left(PP,CT,PK_{uid},{\left\{PSK_{uid,k}^{d}\right\}}_{k\in I_{A}^{e}},SK_{uid}\right)$ algorithm which includes the following three sub-algorithms: Verify(PP,CT) run by any trusted party (public verifiability), $PartialDecryption\left(PP,CT,PK_{uid},{\left\{PSK_{uid,k}^{d}\right\}}_{k\in I_{A}^{e}}\right)$ run by fog nodes and $FullDecryption\left(PP,CT^{p},SK_{uid}\right)$ performed by the user. $I_{A}^{s}$ (resp. $I_{A}^{e}$) denotes the set of the indexes of the authorities involved in signing (resp. encryption). Note that $I_{A}^{s}$ (resp. $I_{A}^{e}$) can be obtained from $R_{s}$ (resp. $R_{e}$) which is implicitly contained in CT.

Verify(PP,CT). This algorithm takes as input the public parameters PP and ciphertext CT, outputs ⊥ if CT contains an invalid signature corresponding to the signing predicate $R_{s}$ embedded in CT. Otherwise, proceed $Decryption\left(PP,CT,PK_{uid},{\left\{PSK_{uid,k}\right\}}_{k\in I_{A}^{e}},SK_{uid}\right)$ algorithm as follows:

$Decryption\left(PP,CT,PK_{uid},{\left\{PSK_{uid,k}\right\}}_{k\in I_{A}^{e}},SK_{uid}\right)$. This algorithm contains two sub-algorithms:

$PartialDecryption\left(PP,CT,PK_{uid},{\left\{PSK_{uid,k}^{d}\right\}}_{k\in I_{A}^{e}}\right)$. This algorithm intakes the public parameters PP, the ciphertext CT, the public key $PK_{uid}$ of the user and the proxy secret decryption key $PSK_{uid,k}^{d}$, outputs the partial decryption result $CT^{p}$ and returns $CT^{p}$ to the user.

$FullDecryption\left(PP,CT^{p},SK_{uid}\right)$. Taking as input the public parameters PP, the partial decryption result $CT^{p}$ and secret key $SK_{uid}$, the algorithm outputs the final plaintext M or ⊥.

(6) Attribute revocation

In this phase, suppose the attribute x of the user U is revoked from $AA_{k}$. After randomly chooses a new attribute version key, the authority $AA_{k}$ distributes the update keys implicitly containing the latest attribute version key to the non-revoked users and cloud server respectively. Only the x-related components of secret keys and ciphertext will be updated.

$UpSecretKeyGen\left(PK_{uid},SK_{k},SK_{uid,k}\right)$. This algorithm is run by attribute authority $AA_{k}$. The algorithm intakes the public key $PK_{uid}$ of non-revoked user $U_{uid}$, the secret key of $AA_{k}$, outputs the signing and decryption update keys $sUK_{uid,x},dUK_{uid,x}$, and ciphertext update keys cUK,sUK.

$UpSecretKey\left(SK_{uid,k},sUK_{uid,x},dUK_{uid,x}\right)$. This algorithm is run by the non-revoked user $U_{uid}$. Taking as input the secret signing and decryption key $SK_{uid,k}$, and the signing and decryption update keys $sUK_{uid,x},dUK_{uid,x}$, the algorithm outputs the updated secret signing and decryption keys.

UpCiphertext(CT,cUK,sUK). This algorithm is run by the cloud server. Taking as input the ciphertext tagged with the revoked attribute, and the ciphertext update keys cUK,sUK, the algorithm outputs the updated ciphertext.

#### 4.2.2. Threat Assumption

Assume CA is fully trusted. The authorities can honestly issue the secret keys for the user and will not collude with the user to access the sensitive data. However, the authorities can be corrupted and disclose the information sent from the data user to the adversary. The fog nodes can also be corrupted and leak the information such as proxy secret keys to the adversary. The cloud server is semi-trusted. It will execute the protocol in general but will leak the signcrypted data to some malicious users and get illegal access privileges. The data users (including the signcryptor and designcryptor) are malicious and can collude with other users and even the cloud server and fog nodes to sign or decrypt the unauthorized data.

#### 4.2.3. Security Requirements

Following [12,14], the confidentiality, unforgeability and signcryptor privacy of OMDAC-ABSC scheme are presented in Definitions 8, 9 and 10 as follows by defining the security games between a challenger and an adversary A. Then in Definition 11 and Definition 12, we provide the definitions of collusion resistance and attribute revocation security.

**Definition** **8.** *Indistinguishability of ciphertext under selective encryption predicate and adaptively chosen ciphertext attack (IND-sEP-CCA2).*

The scheme is $\left(T,q_{sk},q_{psk},q_{SC},q_{DS},\epsilon \right)$-IND-sEP-CCA2 secure if for any PPT adversary A which runs in time at most T and makes at most $q_{sk}$ SecretKey queries, $q_{psk}$ Proxy SecretKey queries, $q_{SC}$ Signcryption queries, and $q_{DS}$ DeSigncryption queries, the advantage $Adv_{A}^{IND-sEP-CCA2}$ of A in the following game with a challenger C is at most ϵ.

Init. A specifies the space of attributes and the set of corrupted authorities. A submits the challenge encryption predicate $R_{e}^{*}=\left(M_{e}^{*},\rho_{e}^{*}\right)$ over encryption attributes that will be used to encrypt the challenge ciphertext. Note that the adversary cannot decrypt the challenge ciphertext with any secret decryption keys queried from SecretKey queries and the keys directly generated from the corrupted authorities.

Setup. The challenger runs the algorithms in system initialization phase to generate the public parameters, and the pairs of public key and the secret key of the attribute authorities. Then the challenger sends the public keys to the adversary. For the corrupted authorities, the challenger sends the secret keys to the adversary.

Phase 1. In this phase, the challenger C answers the queries from A as follows:

$SecretKey\;query\;O^{sk}\left(\tilde{U},AA_{k},uid\right)$. A can adaptively query the secret key for a user U with identity uid and a set of attributes $\tilde{U}=\tilde{U^{d}}\cup \tilde{U^{s}}$ to the authority $AA_{k}$. $\tilde{U^{d}}$ does not satisfy $R_{e}^{*}$ together with any keys that can be obtained from corrupted authorities. The challenger runs SecretKeyGen and returns the secret key to the adversary.

$Proxy\;SecretKey\;query\;O^{psk}\left(\tilde{U},AA_{k},uid\right)$. A can adaptively query the proxy secret key for a user U with identity uid. The challenger runs PxSecretKeyGen and returns the proxy secret key to the adversary.

$Signcryption\;query\;O^{SC}\left(M,R_{s},R_{e}\right)$. Upon receiving a message $M\in G_{T}$, signing and encryption predicts $R_{s},R_{e}$, the challenger C selects a signing attribute set $\tilde{U^{s}}$ such that $R_{s}\left(\tilde{U^{s}}\right)=1$ and returns the ciphertext to the adversary.

$DeSigncryption\;query\;O^{DS}\left(CT,\tilde{U^{d}}\right)$. A submits a ciphertext CT, and a decryption attribute set $\tilde{U^{d}}$. C returns the plaintext to A if $R_{e}\left(\tilde{U^{d}}\right)=1$ and CT contains a valid signature corresponding to the signing predicate $R_{s}$, where $R_{e}$ and $R_{s}$ are implicitly contained in CT.

Challenge. A submits two messages $M_{0},M_{1}$ with the same length and signing predicate $R_{s}^{*}=\left(M_{s}^{*},\rho_{s}^{*}\right)$ to the challenger. C selects a signing attribute set $\tilde{U^{s}}$ satisfying $R_{s}^{*}\left(\tilde{U^{s}}\right)=1$. The challenger randomly chooses a bit 𝒷∈{0,1} and runs the Signcryption algorithm to signcrypt the message $M_{𝒷}$ and returns the ciphertext $CT^{*}$ to A as the challenge ciphertext.

Phase 2. Phase 1 is repeated. In this phase, A cannot issue $O^{DS}$ with the challenge ciphertext $CT^{*}$ obtained in Challenge phase and attribute set $\tilde{U^{d}}$ such that $R_{e}^{*}\left(\tilde{U^{d}}\right)=1$.

Guess. A outputs a guess bit ${𝒷}^{\prime }$ on 𝒷. A wins the game if ${𝒷}^{\prime }=𝒷$.

The advantage of A is defined by $Adv_{A}^{IND-sEP-CCA2}=\left|Pr\left[{𝒷}^{\prime }=𝒷\right]-1/2\right|$.

**Definition** **9.** *Existential unforgeability under selective signing predicate and adaptively chosen message attack (EUF-sSP-CMA).*

The proposed scheme is $\left(T,q_{sk},q_{psk},q_{SC},q_{DS},\epsilon \right)$-EUF-sSP-CMA secure if for any PPT adversary A which runs in time at most T and makes at most $q_{sk}$ SecretKey queries, $q_{psk}$ Proxy SecretKey queries, $q_{SC}$ Signcryption queries, and $q_{DS}$ DeSigncryption queries, the advantage $Adv_{A}^{EUF-sSP-CMA}$ of A in the following game with a challenger C is at most ϵ.

Init. A specifies the space of attributes and a set of corrupted authorities, and then submits the challenge signing predicate $R_{s}^{*}=\left(M_{s}^{*},\rho_{s}^{*}\right)$ over signing attributes that will be used to forge the ciphertext. Note that the adversary cannot sign the plaintext under the signing predicate $R_{s}^{*}$ with any secret signing keys queried from SecretKey queries and the keys directly generated from the corrupted authorities.

Setup,Proxy SecretKey query,Signcryption query and DeSigncryption query are the same as Definition 8.

$SecretKey\;query\;O^{sk}\left(\tilde{U},AA_{k},uid\right)$. A can adaptively query the secret key for a user U with a set of attributes $\tilde{U}=\tilde{U^{d}}\cup \tilde{U^{s}}$ to the authority $AA_{k}$. $\tilde{U^{s}}$ does not satisfy $R_{s}^{*}$ together with any keys that can be obtained from corrupted authorities. The challenger runs SecretKeyGen and returns the secret key to the adversary.

Forgery. A outputs the forgery ciphertext $CT^{*}$ for the selective signing predicate $R_{s}^{*}$ and an arbitrary encryption predicate $R_{e}^{*}$.

A wins the game if $CT^{*}$ is a valid ciphertext and A has never issued $O^{SC}\left(M,R_{s}^{*},R_{e}^{*}\right)$. The advantage of A is defined as $Adv_{A}^{EUF-sSP-CMA}=Pr\left[A\;\mathrm{wins}\right]$.

Note that in our scheme, the fog nodes can be corrupted. In this case, the proxy secret keys sent from the users might be obtained by the adversary. This kind of attack is captured by the proxy secret key query $O^{psk}\left(\tilde{U},AA_{k},uid\right)$, which makes the access control scheme proven secure in our security model have a wider spectrum of applications.

**Definition** **10.** *Signcryptor Privacy.*

It is required that the signature of the proposed scheme reveals nothing about the attributes of the data owner except that the attributes satisfy the signing predicate. We define signcryptor privacy as a game between a challenger C and an adversary A.

Assume the public parameters PP and public and secret key pairs ${\left\{PK_{k},SK_{k}\right\}}_{I_{A}}$ of attribute authorities are given to A. A submits two signing attribute sets $\tilde{U_{0}^{s}},\tilde{U_{1}^{s}}$ satisfying $R_{s}\left(\tilde{U_{0}^{s}}\right)=R_{s}\left(\tilde{U_{1}^{s}}\right)=1$ to the challenger. The challenger then chooses a bit $𝒷\overset{R}{\leftarrow }\left\{0,1\right\}$ and signcrypts the plaintext M with the signing and encryption predicates $R_{s},R_{e}$, and secret signing key $SK_{uid,k}^{s,𝒷}$ for $\tilde{U_{𝒷}^{s}}$. C sends the ciphertext $CT_{𝒷}$ to A. A then outputs a guess bit ${𝒷}^{\prime }$ on 𝒷. A wins the game if ${𝒷}^{\prime }=𝒷$. We say OMDAC-ABSC scheme satisfies signcryptor privacy if for any adversary A,

$$Pr[{𝒷}^{\prime }=𝒷\;:\;\begin{array}{l} PP\leftarrow GlobalSetup1\left(1^{k}\right)\\ {\left\{PK_{k},SK_{k}\right\}}_{I_{A}}\leftarrow AuthoritySetup\left(PP,PPK_{k}\right)\\ \left(\tilde{U_{0}^{s}},\tilde{U_{1}^{s}},M,R_{s},R_{e}\right)\leftarrow A\left(PP,{\left\{PK_{k},SK_{k}\right\}}_{I_{A}}\right)\\ R_{s}\left(\tilde{U_{0}^{s}}\right)=1=R_{s}\left(\tilde{U_{1}^{s}}\right)\\ 𝒷\overset{R}{\leftarrow }\left\{0,1\right\}\\ CT_{𝒷}\leftarrow C\left(M,PP,R_{s},R_{e},\tilde{U_{𝒷}^{s}},{\left\{SK_{uid,k}^{s,𝒷}\right\}}_{k\in I_{A}^{s}}\right)\\ {𝒷}^{\prime }\leftarrow A\left(PP,CT_{𝒷},{\left\{PK_{k},SK_{k}\right\}}_{I_{A}}\right) \end{array}]=\frac{1}{2}$$

**Definition** **11.** *Collusion Resistance.*

OMDAC-ABSC scheme is secure against collusion attack of two or more communication entities (e.g., data users, fog nodes, and cloud server) if there does not exist a set of polynomial time adversaries that can sign the plaintext (collusion resistance of signing) or decrypt the ciphertext (collusion resistance of decryption) by cooperating with each other when none of adversaries is authorized to sign or decrypt the data.

**Definition** **12.** *Suppose the attribute x is revoked.*

Forward Security. If x is the signing attribute, then OMDAC-ABSC scheme supports forward revocation security if the newly joined user can successfully sign the plaintext with the x-corresponding signing attribute set. Otherwise, the forward revocation security guarantees if each newly joined user can decrypt x-corresponding ciphertext if the decryption attributes of the user satisfy the encryption predicate contained in the ciphertext.

Backward Security. If x is the signing attribute, then OMDAC-ABSC scheme supports backward revocation security if the updated ciphertext cannot be reversed back to the non-revoked state while maintaining the verification algorithm holds. Otherwise, the backward revocation security guarantees if the attribute revoked user cannot decrypt the x-corresponding ciphertext as a non-revoked user.

## 5. Construction of OMDAC-ABSC Scheme

In this section, we propose the construction of OMDAC-ABSC scheme in detail. The notations of the scheme are listed in Appendix A.

### 5.1. System Initialization

#### 5.1.1. System Setup 1

$GlobalSetup1\left(1^{k}\right)$. Taking as input a security parameter $1^{k}$, the algorithm outputs the public parameters PP as follows.

(1) Generate a bilinear group $GG\left(1^{k}\right)\to \left(e,p,G,G_{T}\right)$, where the prime p is the order of group G. Let g,θ be the random generators of G. Randomly select $\gamma_{1},\gamma_{2},\;\left\{k_{0},k_{1},\ldots ,k_{l}\right\},\left\{V_{1},V_{2}\ldots ,V_{\ell_{m}}\right\}$ from G. Choose three cryptographic collision resistant hash functions $H_{1}:\;G\to {\mathbb{Z}}_{p}^{*}$, $H_{2}:\;{\left\{0,1\right\}}^{*}\to {\left\{0,1\right\}}^{l}$ and $H_{3}:\;{\left\{0,1\right\}}^{*}\to {\mathbb{Z}}_{p}^{*}$.
(2) CA generates a pair of keys $\left\{sk_{CA},vk_{CA}\right\}$ for signing and verification in identity authentication.
(3) Output $PP=\left\{g,\theta ,\gamma_{1},\gamma_{2},\;\left\{k_{0},k_{1},\ldots ,k_{l}\right\},\left\{V_{1},V_{2}\ldots ,V_{\ell_{m}}\right\}\right\}$ as the system public parameter. CA accepts both user registration UserReg(PP) and authority registration AuthorityReg(PP).

UserReg(PP). CA verifies user U’s identity information then runs this algorithm to register U. CA selects a unique identity number uid and sends $PPK_{uid}=\left\{g^{s_{uid}},g^{d_{uid}},{\left\{V_{i}^{s_{uid}}\right\}}_{i\in \left[\ell_{m}\right]}\right\}$ as the partial public key to user. $s_{uid}$ and $d_{uid}$ are kept secret in the system.

AuthorityReg(PP). CA verifies the identity information of the authority then runs this algorithm to register the authority. CA selects a unique identity number $aid\in \left[1,N_{A}\right]$, then selects $\alpha_{aid}$ and publishes the partial public key $PPK_{aid}=\Delta_{aid}=e{\left(g,g\right)}^{\alpha_{aid}}$ to $AA_{aid}$.

$UserSetup\left(PP,PPK_{uid}\right)$. Given the global identity uid, the user runs $UserSetup\left(PP,PPK_{uid}\right)$ to initialize itself and compute the public key $PK_{uid}$ and secret key $SK_{uid}$ as follows.

1. Set $SK_{uid}=z_{uid}$ where $z_{uid}\overset{R}{\leftarrow }{\mathbb{Z}}_{p}$.
2. Set $PK_{uid}=\left\{g^{s_{uid}},g^{d_{uid}},g^{1/z_{uid}},\theta^{z_{uid}},g^{z_{uid}},{\left\{V_{i}^{s_{uid}}\right\}}_{i\in \left[\ell_{m}\right]}\right\}$.
3. CA sets $cert\left(uid\right)=Sign_{sk_{CA}}\left(uid,PK_{uid}\right)$ as the public key certificate.

$AuthoritySetup\left(PP,PPK_{aid}\right)$. Each authority $AA_{aid}$ runs this algorithm to initialize itself and compute the public key $PK_{aid},PK_{uid,aid}^{1}$ and secret key $SK_{aid}$ as follows:

(1) Set $SK_{aid}=\left\{\beta_{aid},\gamma_{aid},{\left\{\varphi_{x}\right\}}_{x\in \tilde{AA_{aid}}}\right\}$, where $\beta_{aid},\gamma_{aid},\varphi_{x}\overset{R}{\leftarrow }{\mathbb{Z}}_{p}$.
(2) Set $PK_{aid}=\left\{\Delta_{aid},X_{aid},Y_{aid},Z_{aid},{\left\{A_{x}\right\}}_{x\in \tilde{AA_{aid}}}\right\}$, where $A_{x}=g^{\varphi_{x}},X_{aid}=g^{1/\beta_{aid}},Y_{aid}=\theta^{1/\beta_{aid}},Z_{aid}=\theta^{1/\gamma_{aid}}$.
(3) Set $PK_{uid,aid}^{1}=g^{1/\left(\gamma_{aid}z_{uid}\right)}$ for each user $U_{uid}\in S_{U}$.

#### 5.1.2. System Setup 2

$GlobalSetup2\left(1^{k},PP,\;{\left\{PK_{aid},PK_{uid,aid}^{1}\right\}}_{U_{uid}\in S_{U},AA_{aid}\in S_{A}}\right)$. Taking as input the public parameters PP and authorities’ public keys ${\left\{PK_{aid},PK_{uid,aid}^{1}\right\}}_{U_{uid}\in S_{U},AA_{aid}\in S_{A}}$, CA generates the public key $PK_{uid,aid}$ for each pair of user $U_{uid}$ and authority $AA_{aid}$ as follows:

For $U_{uid}\in S_{U},AA_{aid}\in S_{A}$, $PK_{uid,aid}=\left\{PK_{uid,aid}^{1},PK_{uid,aid}^{2},PK_{uid,aid}^{3}\right\}$, where $PK_{uid,aid}^{2}={\left(PK_{uid,aid}^{1}\right)}^{\alpha_{aid}}Z_{aid}^{d_{uid}}=g^{\alpha_{aid}/\left(\gamma_{aid}z_{uid}\right)}\theta^{d_{uid}/\gamma_{aid}}$ and $PK_{uid,aid}^{3}=X_{aid}^{\alpha_{aid}}Y_{aid}^{s_{uid}}=g^{\alpha_{aid}/\beta_{aid}}\theta^{s_{uid}/\beta_{aid}}$.

### 5.2. Secret Key Generation

$AA_{aid}$ runs the secret key generation algorithm SecretKeyGen to generate the secret signing and decryption keys for the user $U_{uid}$.

$SecretKeyGen\left(PP,PK_{aid},SK_{aid},PK_{uid},\tilde{U}\right)$. $AA_{aid}$ first verifies the user’s cert(uid) with verification key $vk_{CA}$. If the user is a legal user, $AA_{aid}$ computes the user’s secret signing and decryption keys $SK_{uid,aid}=\left\{SK_{uid,aid}^{s},SK_{uid,aid}^{d}\right\}$ as:

(1) $SK_{uid,aid}^{s}=\left\{K_{uid,aid}^{s}={\left(PK_{uid,aid}^{3}\right)}^{\beta_{aid}}=g^{\alpha_{aid}}\theta^{s_{uid}},{\left\{F_{uid,x}^{s}={\left(g^{s_{uid}}\right)}^{\varphi_{x}}=A_{x}^{s_{uid}}\right\}}_{x\in \tilde{U^{s}}\cap \tilde{AA_{aid}}}\right\}$.
(2) $SK_{uid,aid}^{d}=\left\{K_{uid,aid}^{d}={\left(PK_{uid,aid}^{2}\right)}^{\gamma_{aid}}=g^{\alpha_{aid}/z_{uid}}\theta^{d_{uid}},{\left\{F_{uid,x}^{d}={\left(g^{d_{uid}}\right)}^{\varphi_{x}}=A_{x}^{d_{uid}}\right\}}_{x\in \tilde{U^{d}}\cap \tilde{AA_{aid}}}\right\}$.

### 5.3. Proxy Secret Key Generation

Each user $U_{uid}$ runs the $PxSecretKeyGen\left(SK_{uid},SK_{uid,aid}\right)$ to generate the proxy secret key $PSK_{uid,aid}=\left\{PSK_{uid,aid}^{s},PSK_{uid,aid}^{d}\right\}$ as:

(1) $PSK_{uid,aid}^{s}=\left\{PS_{uid,aid}={\left(K_{uid,aid}^{s}\right)}^{z_{uid}},PV_{uid}=g^{z_{uid}s_{uid}},{\left\{PF_{uid,x}^{1}={\left(F_{uid,x}^{s}\right)}^{z_{uid}},PF_{uid,x}^{2}={\left(A_{x}\right)}^{z_{uid}}\right\}}_{x\in \tilde{U^{s}}\cap \tilde{AA_{aid}}},{\left\{V_{i}^{z_{uid}},V_{i}^{s_{uid}z_{uid}}\right\}}_{i\in \left[\ell_{m}\right]}\right\}$.
(2) $PSK_{uid,aid}^{d}=SK_{uid,aid}^{d}$.

The transformed secret keys $\left\{PSK_{uid,aid}\right\}$ are sent to the fog node.

### 5.4. Data Signcryption

The data owner first encrypts the data component with a content secret key k by using symmetric encryption algorithm $En_{k}$, then it runs Signcryption to signcrypt the secret key. Signcryption contains two phases: fog signcrypt Fog_Signcryption and user signcrypt User_Signcryption.

$Signcryption\left(ℳ,PP,{\left\{PSK_{uid,k}^{s}\right\}}_{k\in I_{A}^{s}},PK_{uid},{\left\{PK_{aid}\right\}}_{aid\in I_{A}^{e}},SK_{uid},{ℛ}_{s},{ℛ}_{e}\right)$. Assume that ${ℛ}_{s}≔\left(M_{s},\rho_{s}\right)$ (resp. ${ℛ}_{e,j}≔\left(M_{e},\rho_{e}\right)$) is the signing predicate (resp. encryption predicate) over all the attributes selected from the set of attribute authorities $I_{A}^{s}$ (resp. $I_{A}^{e}$), where $M_{s}$ (resp. $M_{e}$) is a $\ell_{s}\times n_{s}$, $\ell_{s}\leq \ell_{m}$ (resp. $\ell_{e}\times n_{e}$) matrix with row labeling function $\rho_{s}:\;\left[\ell_{s}\right]\to {\mathbb{Z}}_{p}$ (resp. $\rho_{e}:\;\left[\ell_{e}\right]\to {\mathbb{Z}}_{p}$). Note that we remove the limitation that $\rho_{s}$ (resp. $\rho_{e}$) should be an injective function (i.e., an attribute can associate with more than one rows of $M_{s}$ (resp. $M_{e}$)). Let $M_{s}^{i}$ (resp. $M_{e}^{i}$) be the ith row of the matrix $M_{s}$ (resp. $M_{e}$). Assume the signing attribute set is $\tilde{U^{s}}$ and ${ℛ}_{s}\left(\tilde{U^{s}}\right)=1$. The algorithm contains two phases as follows:

(1) $Fog\_Signcryption\left(PP,{\left\{PSK_{uid,k}^{s}\right\}}_{k\in I_{A}^{s}},PK_{uid},{ℛ}_{s},{ℛ}_{e}\right)$. This algorithm is performed in the fog node FD as follows:
  - It first computes a vector $\vec{a}=\left(a_{1},a_{2},\ldots ,a_{\ell_{s}}\right)\in {\mathbb{Z}}_{p}^{\ell_{s}}$ such that $\vec{a}\cdot M_{s}=\vec{1}$ since ${ℛ}_{s}\left(\tilde{U^{s}}\right)=1$. Note that $a_{i}=0$ for all i where $\rho_{s}\left(i\right)\notin \tilde{U^{s}}$. Then the algorithm chooses $\vec{b}=\left(b_{1},b_{2},\ldots ,b_{\ell_{s}}\right)\in {\mathbb{Z}}_{p}^{\ell_{s}}$ such that $\sum_{i\in \left[\ell_{s}\right]}b_{i}M_{s}^{i}=\vec{0}$.
  - The algorithm randomly chooses $s_{uid}^{\prime }\overset{R}{\leftarrow }{\mathbb{Z}}_{p}^{*}$ and re-randomize the proxy secret key $PSK_{uid,aid}^{s}$ as
    $PS_{uid,k}=PS_{uid,k}{\left(\theta^{z_{uid}}\right)}^{s_{uid}^{\prime }}=g^{\alpha_{k}z_{uid}}\theta^{z_{uid}s_{uid}^{\prime \prime }}$,
    $PV_{uid}=PV_{uid}{\left(g^{z_{uid}}\right)}^{s_{uid}^{\prime }}=g^{z_{uid}s_{uid}^{\prime \prime }}$,
    ${\left\{PF_{uid,x}=PF_{uid,x}^{1}{\left(PF_{uid,x}^{2}\right)}^{s_{uid}^{\prime }}=A_{x}^{z_{uid}s_{uid}^{\prime \prime }}\right\}}_{x\in \tilde{U^{s}}}$,
    $V_{i}^{z_{uid}s_{uid}^{\prime \prime }}=V_{i}^{s_{uid}z_{uid}}{\left(V_{i}^{z_{uid}}\right)}^{s_{uid}^{\prime }}$, where $s_{uid}^{\prime \prime }=s_{uid}+s_{uid}^{\prime }$.
  - The fog node randomly picks $w^{\prime }\overset{R}{\leftarrow }{\mathbb{Z}}_{p}^{*}$. Then it selects $\left\{r_{1}^{\prime },r_{2}^{\prime },\ldots ,r_{\ell_{e}}^{\prime }\right\}\overset{R}{\leftarrow }{\mathbb{Z}}_{p}$, $\left\{\lambda_{1}^{\prime },\lambda_{2}^{\prime },\ldots ,\lambda_{\ell_{e}}^{\prime }\right\}\overset{R}{\leftarrow }{\mathbb{Z}}_{p}$, and computes the following terms: ${\left\{C_{2,i}^{\prime }=\theta^{\lambda_{i}^{\prime }}A_{\rho_{e}\left(i\right)}^{-r_{i}^{\prime }},D_{i}^{\prime }=g^{r_{i}^{\prime }}\right\}}_{i\in \left[\ell_{e}\right]}$, ${\left\{S_{1,i}^{\prime }=PV_{uid}^{a_{i}}g^{z_{uid}b_{i}}=g^{z_{uid}\left(a_{i}s_{uid}^{\prime \prime }+b_{i}\right)}\right\}}_{i\in \left[\ell_{s}\right]}$. $S_{2}^{\prime }=\left(\prod_{I_{A}^{s}}PS_{uid,k}\right)\left(\prod_{i\in \left[\ell_{s}\right]}{\left(PF_{uid,\rho_{s}\left(i\right)}V_{i}^{z_{uid}s_{uid}^{\prime \prime }}\right)}^{a_{i}}{\left(PF_{uid,\rho_{s}\left(i\right)}^{2}V_{i}^{z_{uid}}\right)}^{b_{i}}\right)$.

FD outputs the partially signcrypted ciphertext $CT^{\prime }=\left\{w^{\prime },{\left\{C_{2,i}^{\prime },D_{i}^{\prime },\lambda_{i}^{\prime },r_{i}^{\prime }\right\}}_{i\in \left[\ell_{e}\right]},{\left\{S_{1,i}^{\prime }\right\}}_{i\in \left[\ell_{s}\right]},S_{2}^{\prime }\right\}$ to the user.

(2) $User\_Signcryption\left(ℳ,PP,{\left\{PK_{aid}\right\}}_{aid\in I_{A}^{e}},SK_{uid},CT^{\prime }\right)$. The user randomly picks $w\overset{R}{\leftarrow }{\mathbb{Z}}_{p}^{*}$ and $\left\{r_{1},r_{2},\ldots ,r_{\ell_{e}}\right\}\overset{R}{\leftarrow }{\mathbb{Z}}_{p}$. Then the user computes $\lambda_{i}=M_{e}^{i}\vec{\varepsilon }$ where $\vec{\varepsilon }=\left(w,\varepsilon_{2},\ldots ,\varepsilon_{n_{e}}\right)\in {\mathbb{Z}}_{p}^{n_{e}}$. The algorithm computes the following terms:

$C_{0}=ℳ\prod_{k\in I_{A}^{e}}\Delta_{k}{}^{w}$, $C_{1}=g^{w}$, ${\left\{C_{2,i}^{\prime \prime }=\lambda_{i}-\lambda_{i}^{\prime },D_{i}^{\prime \prime }=r_{i}-r_{i}^{\prime }\right\}}_{i\in \left[\ell_{e}\right]}$, $\pi =H_{1}\left(C_{1}\right)$, $C_{3}={\left(\gamma_{1}\gamma_{2}{}^{\pi }\right)}^{w}$, ${\left\{S_{1,i}={\left(S_{1,i}^{\prime }\right)}^{1/z_{uid}}\right\}}_{i\in \left[\ell_{s}\right]}$, $H_{2}\left(\prod_{i\in \left[\ell_{s}\right]}S_{1,i},tt,{ℛ}_{s},{ℛ}_{e}\right)=\left(c_{1},c_{2},\ldots ,c_{l}\right)\in {\left\{0,1\right\}}^{l}$, $H_{3}\left(C_{0},C_{1},C_{3},{ℛ}_{s},{ℛ}_{e}\right)=\beta$ and $S_{2}={\left(S_{2}^{\prime }\right)}^{1/z_{uid}}{\left(k_{0}\prod_{i=1}^{l}k_{i}{}^{c_{i}}\right)}^{w}C_{3}^{\beta }$.

The ciphertext is $CT=\left\{C_{0},C_{1},{\left\{C_{2,i}^{\prime },C_{2,i}^{\prime \prime },D_{i}^{\prime },D_{i}^{\prime \prime }\right\}}_{i\in \left[\ell_{e}\right]},{\left\{S_{1,i}\right\}}_{i\in \left[\ell_{s}\right]},S_{2},tt\right\}$.

### 5.5. Data Designcryption

If the owner’s attributes satisfy the signing predicate implicitly contained in the ciphertext, then any party can successfully verify the ciphertext (public verifiability). If the receiver’s decryption attributes satisfy the encryption predicates embedded in the ciphertext, then the decryption phase can be launched to access the plaintext.

$DeSigncryption\left(PP,CT,PK_{uid},{\left\{PSK_{uid,k}^{d}\right\}}_{k\in I_{A}^{e}},SK_{uid}\right)$. Assume that $thre_{tt}$ is a predefined time threshold for designcryption and $\tilde{tt}$ is the current time. If $\left|\tilde{tt}-tt\right|>thre_{tt}$ or ${ℛ}_{e}\left(\tilde{U^{d}}\right)\neq 1$, the algorithm returns ⊥. Otherwise, the algorithm performs as follows. Note that $I_{A}^{s}$ (resp. $I_{A}^{e}$) can be obtained from the implicitly contained predicate ${ℛ}_{s}$ (resp. ${ℛ}_{e}$).

Verify(PP,CT). This verification algorithm can be performed in FD or other trusted third party since it only takes the ciphertext and public parameter PP as the input.

The algorithm samples $\left\{\tau_{2},\tau_{3},\ldots ,\tau_{n_{s}}\right\}\overset{R}{\leftarrow }{\mathbb{Z}}_{p}^{*}$ and computes $\varpi_{i}=\left(1,\tau_{2},\tau_{3},\ldots ,\tau_{n_{s}}\right)\cdot M_{s}^{i}$, where $i\in \left[\ell_{s}\right]$. $H_{1}\left(C_{1}\right)=\pi$ and $H_{2}\left(\prod_{i\in \left[\ell_{s}\right]}S_{1,i},tt,{ℛ}_{s},{ℛ}_{e}\right)=\left(c_{1},c_{2},\ldots ,c_{l}\right)$, and $H_{3}\left(C_{0},C_{1},C_{3},{ℛ}_{s},{ℛ}_{e}\right)=\beta$. Then the algorithm checks the validity of the ciphertext using the following equation:

$\prod_{I_{A}^{s}}\Delta_{k}=\frac{e\left(S_{2},g\right)}{e\left(k_{0}\prod_{i=1}^{l}k_{i}{}^{c_{i}},C_{1}\right)e\left({\left(\gamma_{1}\gamma_{2}{}^{\pi }\right)}^{\beta },C_{1}\right)\left(\prod_{i=1}^{\ell_{s}}e\left(A_{\rho_{s}\left(i\right)}V_{i}\theta^{\varpi_{i}N_{A}^{s}},S_{1,i}\right)\right)}$, where $N_{A}^{s}=\left|I_{A}^{s}\right|$.

If it is invalid, return ⊥, otherwise, proceed $Decryption\left(PP,CT,PK_{uid},{\left\{PSK_{uid,k}\right\}}_{k\in I_{A}^{e}},SK_{uid}\right)$ algorithm as follows:

- $PartialDecryption\left(PP,CT,PK_{uid},{\left\{PSK_{uid,k}^{d}\right\}}_{k\in I_{A}^{e}}\right)$. If the user’s attributes satisfy the encryption predicate, the cloud server sends the ciphertext to the FD. FD chooses a set of constants $\vec{\sigma }=\left(\sigma_{1},\sigma_{2},\ldots ,\sigma_{\ell_{e}}\right)\in {\mathbb{Z}}_{p}^{\ell_{e}}$ such that $\sum_{i=1}^{\ell_{e}}\sigma_{i}M_{e}^{i}=\vec{1}$, where $\sigma_{i}=0$ for all i where $\rho_{e}\left(i\right)\notin \tilde{U^{d}}$. Then it computes: $CT_{x}=\frac{\prod_{k\in I_{A}^{e}}e\left(K_{uid,k}^{d},C_{1}\right)}{\prod_{k\in I_{A}^{e}}\prod_{i\in I_{A_{k}}}{\left[e\left(C_{2,i}^{\prime }\theta^{C_{2,i}^{\prime \prime }}A_{\rho_{e}\left(i\right)}^{-D_{i}^{\prime \prime }},g^{d_{uid}}\right)e\left(D_{i}^{\prime }g^{D_{i}^{\prime \prime }},F_{uid,\rho_{e}\left(i\right)}^{d}\right)\right]}^{\sigma_{i}N_{A}^{e}}}$, where $I_{A_{k}}$ is defined as $I_{A_{k}}=\left\{i:\rho_{e}\left(i\right)\in \tilde{AA_{k}}\right\}$. FD sends $CT^{p}=\left\{C_{0},CT_{x}\right\}$ to the user.
- $FullDecryption\left(PP,CT^{p},SK_{uid}\right)$. This algorithm is performed on the user side. After receiving $CT^{p}$, the data user recovers the message ℳ as: $ℳ=\frac{C_{0}}{{\left(CT_{x}\right)}^{z_{uid}}}$.

*Correctness*

Assume the identity of signcryptor (data owner) is do. If $\left|\tilde{tt}-tt\right|\leq thre_{tt}$ and ${ℛ}_{e}\left(\tilde{U^{d}}\right)=1$, then the ciphertext can be verified and decrypted as explained subsequently.

$$\begin{array}{l} S_{2}={\left(S_{2}^{\prime }\right)}^{1/z_{do}}{\left(k_{0}\prod_{i=1}^{l}k_{i}{}^{b_{i}}\right)}^{w}C_{3}^{\beta }\\ =\left(\prod_{I_{A}^{s}}g^{\alpha_{k}}\theta^{s_{do}^{\prime \prime }}\right)\left(\prod_{i\in \left[\ell_{s}\right]}{\left(A_{\rho_{s}\left(i\right)}^{s_{do}^{\prime \prime }}V_{i}^{s_{do}^{\prime \prime }}\right)}^{a_{i}}{\left(A_{\rho_{s}\left(i\right)}V_{i}\right)}^{b_{i}}\right){\left(k_{0}\prod_{i=1}^{l}k_{i}{}^{c_{i}}\right)}^{w}{\left(\gamma_{1}\gamma_{2}{}^{\pi }\right)}^{w\beta }\\ =\left(\prod_{I_{A}^{s}}g^{\alpha_{k}}\right)\theta^{s_{do}^{\prime \prime }N_{A}^{s}}\left(\prod_{i\in \left[\ell_{s}\right]}{\left(A_{\rho_{s}\left(i\right)}V_{i}\right)}^{a_{i}s_{do}^{\prime \prime }+b_{i}}\right){\left(k_{0}\prod_{i=1}^{l}k_{i}{}^{c_{i}}\right)}^{w}{\left(\gamma_{1}\gamma_{2}{}^{\pi }\right)}^{w\beta } \end{array}$$

Since $\vec{a}\cdot M_{s}=\vec{1}$ and $\sum_{i\in \left[\ell_{s}\right]}b_{i}M_{s}^{i}=\vec{0}$, we have

$\sum_{i=1}^{\ell_{s}}\varpi_{i}\left(s_{do}^{\prime \prime }a_{i}+b_{i}\right)=\sum_{i=1}^{\ell_{s}}\left(1,\tau_{2},\tau_{3},\ldots ,\tau_{n_{s}}\right)\cdot M_{s}^{i}\left(s_{do}^{\prime \prime }a_{i}+b_{i}\right)=\left(1,\tau_{2},\tau_{3},\ldots ,\tau_{n_{s}}\right)s_{do}^{\prime \prime }\sum_{i=1}^{\ell_{s}}M_{s}^{i}a_{i}+\left(1,\tau_{2},\tau_{3},\ldots ,\tau_{n_{s}}\right)\sum_{i=1}^{\ell_{s}}M_{s}^{i}b_{i}=s_{do}^{\prime \prime }$. Thus we have

$$\begin{array}{l} \frac{e\left(S_{2},g\right)}{e\left(k_{0}\prod_{i=1}^{l}k_{i}{}^{c_{i}},C_{1}\right)e\left({\left(\gamma_{1}\gamma_{2}{}^{\pi }\right)}^{\beta },C_{1}\right)\left(\prod_{i=1}^{\ell_{s}}e\left(A_{\rho_{s}\left(i\right)}V_{i}\theta^{\varpi_{i}N_{A}^{s}},S_{1,i}\right)\right)}\\ =\frac{e\left(\left(\prod_{I_{A}^{s}}g^{\alpha_{k}}\right)\theta^{s_{do}^{\prime \prime }N_{A}^{s}}\left(\prod_{i\in \left[\ell_{s}\right]}{\left(A_{\rho_{s}\left(i\right)}V_{i}\right)}^{a_{i}s_{do}^{\prime \prime }+b_{i}}\right){\left(k_{0}\prod_{i=1}^{l}k_{i}{}^{c_{i}}\right)}^{w}{\left(\gamma_{1}\gamma_{2}{}^{\pi }\right)}^{w\beta },g\right)}{e\left(k_{0}\prod_{i=1}^{l}k_{i}{}^{c_{i}},g^{w}\right)e\left({\left(\gamma_{1}\gamma_{2}{}^{\pi }\right)}^{\beta },g^{w}\right)\left(\prod_{i=1}^{\ell_{s}}e\left(A_{\rho_{s}\left(i\right)}V_{i},g^{a_{i}s_{do}^{\prime \prime }+b_{i}}\right)\right){\left(\prod_{i=1}^{\ell_{s}}e\left(\theta^{\varpi_{i}},g^{a_{i}s_{do}^{\prime \prime }+b_{i}}\right)\right)}^{N_{A}^{s}}}\\ =\frac{e\left(\left(\prod_{I_{A}^{s}}g^{\alpha_{k}}\right)\theta^{s_{do}^{\prime \prime }N_{A}^{s}},g\right)}{{\left(e{\left(\theta ,g\right)}^{s_{do}^{\prime \prime }}\right)}^{N_{A}^{s}}}=\prod_{I_{A}^{s}}\Delta_{k} \end{array}$$

This demonstrates the correctness of Verify algorithm. Assume the identity of designcryptor (data user) is uid. If $\sum_{i=1}^{\ell_{e}}\sigma_{i}\lambda_{i}=w$, then

$$\begin{array}{l} \prod_{k\in I_{A}^{e}}\prod_{i\in I_{A_{k}}}{\left[e\left(C_{2,i}^{\prime }\theta^{C_{2,i}^{\prime \prime }}A_{\rho_{e}\left(i\right)}^{-D_{i}^{\prime \prime }},g^{d_{uid}}\right)e\left(D_{i}^{\prime }g^{D_{i}^{\prime \prime }},F_{uid,\rho_{e}\left(i\right)}^{d}\right)\right]}^{\sigma_{i}N_{A}^{e}}\\ \;=\prod_{k\in I_{A}^{e}}\prod_{i\in I_{A_{k}}}{\left[e\left(\theta^{\lambda_{i}}A_{\rho_{e}\left(i\right)}^{-r_{i}},g^{d_{uid}}\right)e\left(g^{r_{i}},F_{uid,\rho_{e}\left(i\right)}^{d}\right)\right]}^{\sigma_{i}N_{A}^{e}}\\ \;=\prod_{k\in I_{A}^{e}}\prod_{i\in I_{A_{k}}}{\left[e\left(\theta^{\lambda_{i}},g^{d_{uid}}\right)\right]}^{\sigma_{i}N_{A}^{e}}=e{\left(\theta ,g\right)}^{wN_{A}^{e}d_{uid}} \end{array}$$

Hence $CT_{x}=\frac{\prod_{k\in I_{A}}e\left(K_{uid,k}^{d},C_{1}\right)}{\prod_{k\in I_{A}^{e}}\prod_{i\in I_{A_{k}}}{\left[e\left(C_{2,i}^{\prime }\theta^{C_{2,i}^{\prime \prime }}A_{\rho_{e}\left(i\right)}^{-D_{i}^{\prime \prime }},g^{d_{uid}}\right)e\left(D_{i}^{\prime }g^{D_{i}^{\prime \prime }},F_{uid,\rho_{e}\left(i\right)}^{d}\right)\right]}^{\sigma_{i}N_{A}^{e}}}=\frac{\prod_{k\in I_{A}^{e}}e\left(g^{\alpha_{k}/z_{uid}}\theta^{d_{uid}},g^{w}\right)}{e{\left(\theta ,g\right)}^{wN_{A}^{e}d_{uid}}}=\prod_{k\in I_{A}^{e}}e{\left(g^{\alpha_{k}},g\right)}^{w/z_{uid}}=\prod_{k\in I_{A}^{e}}\Delta_{k}{}^{w/z_{uid}}$ and $\frac{C_{0}}{{\left(CT_{x}\right)}^{z_{uid}}}=\frac{ℳ\prod_{k\in I_{A}^{e}}\Delta_{k}{}^{w}}{\prod_{k\in I_{A}^{e}}\Delta_{k}{}^{w}}=ℳ$. This exhibits the correctness of Decryption algorithm.

### 5.6. Attribute Revocation

Suppose the attribute x of user U is revoked from $AA_{k}$.

$UpSecretKeyGen\left(PK_{uid},SK_{k},SK_{uid,k}\right)$. $AA_{k}$ randomly chooses a new attribute version key $\varphi_{x}^{\prime }\overset{R}{\leftarrow }{\mathbb{Z}}_{p}$ and computes the updated attribute public key $A_{x}^{\prime }=g^{\varphi_{x}^{\prime }}$. $AA_{j}$ sets $dUK_{uid,x}=g^{d_{uid}\left(\varphi_{x}^{\prime }-\varphi_{x}\right)},sUK_{uid,x}=g^{s_{uid}\left(\varphi_{x}^{\prime }-\varphi_{x}\right)}$ for the non-revoked users to update their secret decryption and signing keys.

If there exists i such that $\rho_{e}\left(i\right)=x$, namely the attribute x of $AA_{k}$ is selected as the encryption attribute, then $AA_{k}$ queries $D_{i}^{\prime }$ where $\rho_{e}\left(i\right)=x$. Then it computes $cUK={\left\{cUK_{i}={\left(D_{i}^{\prime }\right)}^{\varphi_{x}-\varphi_{x}^{\prime }}\right\}}_{\rho_{e}\left(i\right)=x}$, and sets sgUK=⊥.

Otherwise, if x is selected as the signing attribute, $AA_{k}$ sets cUK=⊥ and $sgUK=\prod_{i=1}^{ℒ}S_{1,i}{}^{\varphi_{x}^{\prime }-\varphi_{x}}$, where ℒ is the set consisting of all the rows that $\rho_{s}\left(i\right)=x$.

$AA_{k}$ sends ciphertext update keys cUK,sUK to the cloud server to update the corresponding ciphertext.

$UpSecretKey\left(SK_{uid,k},sUK_{uid,x},dUK_{uid,x}\right)$. Upon receiving the update keys $sUK_{uid,x}$ and $dUK_{uid,x}$, the non-revoked user $U_{uid}\neq U$ then update his/her secret signing key or decryption key as follows:

If $x\in \tilde{U^{s}}$, $F_{uid,x}^{s}{}^{\prime }=F_{uid,x}^{s}sUK_{uid,x}={\left(A_{x}^{\prime }\right)}^{s_{uid}}$.

If $x\in \tilde{U^{d}}$, $F_{uid,x}^{d}{}^{\prime }=F_{uid,x}^{d}dUK_{uid,x}={\left(A_{x}^{\prime }\right)}^{d_{uid}}$.

UpCiphertext(CT,cUK,sUK). Upon receiving cUK,sUK, the cloud server updates the ciphertext to contain the latest attribute version key as follows:

If $cUK={\left\{cUK_{i}={\left(D_{i}^{\prime }\right)}^{\varphi_{x}-\varphi_{x}^{\prime }}\right\}}_{\rho_{e}\left(i\right)=x}$ and sgUK=⊥, the server randomly chooses ${\left\{r_{i}^{\prime \prime }\in {\mathbb{Z}}_{p}\right\}}_{\rho_{e}\left(i\right)=x}$ and computes $C_{2,i}^{\prime }=C_{2,i}^{\prime }cUK_{i}A_{\rho_{e}\left(i\right)}^{\prime }{}^{-r_{i}^{\prime \prime }}=\theta^{\lambda_{i}^{\prime }}A_{\rho_{e}\left(i\right)}^{\prime }{}^{-\left(r_{i}^{\prime }+r_{i}^{\prime \prime }\right)}$.

$D_{i}^{\prime }=D_{i}^{\prime }g^{r_{i}^{\prime \prime }}=g^{r_{i}^{\prime }+r_{i}^{\prime \prime }}$, where $\rho_{e}\left(i\right)=x$.

Otherwise, the cloud server updates the signature component $S_{2}$ as: $S_{2}=S_{2}sgUK=\left(\prod_{I_{A}^{s}}g^{\alpha_{k}}\theta^{s_{do}^{\prime \prime }}\right)\left(\prod_{i\in \left[\ell_{s}\right]}{\left(A_{\rho_{s}\left(i\right)}V_{i}\right)}^{a_{i}s_{do}^{\prime \prime }+b_{i}}\right){\left(k_{0}\prod_{i=1}^{l}k_{i}{}^{c_{i}}\right)}^{w}C_{3}^{\beta }sgUK=\left(\prod_{I_{A}^{s}}g^{\alpha_{k}}\theta^{s_{do}^{\prime \prime }}\right)\left(\prod_{i\in \left[\ell_{s}\right]\backslash ℒ}{\left(A_{\rho_{s}\left(i\right)}\right)}^{a_{i}s_{do}^{\prime \prime }+b_{i}}\right)\left(\prod_{i\in ℒ}{\left(A_{\rho_{s}\left(i\right)}^{\prime }\right)}^{a_{i}s_{do}^{\prime \prime }+b_{i}}\right)\left(\prod_{i\in \left[\ell_{s}\right]}V_{i}^{a_{i}s_{do}^{\prime \prime }+b_{i}}\right){\left(k_{0}\prod_{i=1}^{l}k_{i}{}^{c_{i}}\right)}^{w}C_{3}^{\beta }$

*Correctness of Attribute Revocation.*

By running $UpSecretKey\left(SK_{uid,k},sUK_{uid,x},dUK_{uid,x}\right)$, the secret signing and decryption keys of non-revoked user $U_{uid}$ are associated with the new attribute version key $\varphi_{x}^{\prime }$, which is the same as the updated ciphertext components ${\left\{C_{2,i}^{\prime }=\theta^{\lambda_{i}^{\prime }}A_{\rho_{e}\left(i\right)}^{\prime }{}^{-\left(r_{i}^{\prime }+r_{i}^{\prime \prime }\right)}\right\}}_{\rho_{e}\left(i\right)=x}$ or $S_{2}=\left(\prod_{I_{A}^{s}}g^{\alpha_{k}}\theta^{s_{do}^{\prime \prime }}\right)\left(\prod_{i\in \left[\ell_{s}\right]ℒ}{\left(A_{\rho_{s}\left(i\right)}\right)}^{a_{i}s_{do}^{\prime \prime }+b_{i}}\right)\left(\prod_{i\in ℒ}{\left(A_{\rho_{s}\left(i\right)}^{\prime }\right)}^{a_{i}s_{do}^{\prime \prime }+b_{i}}\right)\left(\prod_{i\in \left[\ell_{s}\right]}V_{i}^{a_{i}s_{do}^{\prime \prime }+b_{i}}\right){\left(k_{0}\prod_{i=1}^{l}k_{i}{}^{c_{i}}\right)}^{w}C_{3}^{\beta }$.

For verification, since the updated signature component $S_{2}$ is associated with $A_{\rho_{s}\left(i\right)}^{\prime }$ for i such that $\rho_{s}\left(i\right)=x$, we have $\frac{e\left(S_{2},g\right)}{e\left(k_{0}\prod_{i=1}^{l}k_{i}{}^{c_{i}},C_{1}\right)e\left({\left(\gamma_{1}\gamma_{2}{}^{\pi }\right)}^{\beta },C_{1}\right)\left(\prod_{i\in \left[\ell_{s}\right]ℒ}e\left(A_{\rho_{s}\left(i\right)},S_{1,i}\right)\right)\left(\prod_{i\in ℒ}e\left(A_{\rho_{s}\left(i\right)}^{\prime },S_{1,i}\right)\right)\left(\prod_{i\in \left[\ell_{s}\right]}e\left(V_{i}\theta^{\varpi_{i}N_{A}^{s}},S_{1,i}\right)\right)}=\prod_{I_{A}^{s}}\Delta_{k}$, which exhibits the correctness of Verify algorithm.

Additionally, the operations $C_{2,i}^{\prime }=C_{2,i}^{\prime }cUK_{i}A_{\rho_{e}\left(i\right)}^{\prime }{}^{-r_{i}^{\prime \prime }}$ and $D_{i}^{\prime }=D_{i}^{\prime }g^{r_{i}^{\prime \prime }}=g^{r_{i}^{\prime }+r_{i}^{\prime \prime }}$ are equivalent to assigning a new random number $r_{i}^{\prime }+r_{i}^{\prime \prime }$ to the corresponding components of ciphertext. Then in $PartialDecryption\left(PP,CT,PK_{uid},{\left\{PSK_{uid,k}^{d}\right\}}_{k\in I_{A}^{e}}\right)$ algorithm, we have $e\left(C_{2,i}^{\prime }\theta^{C_{2,i}^{\prime \prime }}A_{\rho_{e}\left(i\right)}^{\prime }{}^{-D_{i}^{\prime \prime }},g^{d_{uid}}\right)e\left(D_{i}^{\prime }g^{D_{i}^{\prime \prime }},F_{uid,\rho_{e}\left(i\right)}^{d}\right)=e\left(\theta^{\lambda_{i}}A_{\rho_{e}\left(i\right)}^{\prime }{}^{-r_{i}-r_{i}^{\prime \prime }},g^{d_{uid}}\right)e\left(g^{r_{i}+r_{i}^{\prime \prime }},{\left(A_{\rho_{e}\left(i\right)}^{\prime }\right)}^{d_{uid}}\right)=e\left(\theta^{\lambda_{i}},g^{d_{uid}}\right)$ for i such that $\rho_{e}\left(i\right)=x$, which exhibits the correctness of Decryption algorithm.

## 6. Security Analysis

In this section, we state the security of our OMDAC-ABSC scheme in the following theorems. In Theorems 1 and 2, we prove the message confidentiality and ciphertext unforgeability of our scheme respectively. In Theorem 3 we demonstrate the signcryptor privacy. Then in Theorems 4 and 5, we analyze the collusion resistance and revocation security.

Throughout this section, assume $T^{e}$ is the cost time for one exponentiation in group G or $G_{T}$, and $T^{p}$ is the cost time for one pairing operation. $\ell_{e,m},n_{e,m},\ell_{s,m},n_{s,m}$ are the maximum values of $\left\{\ell_{e},n_{e},\ell_{s},n_{s}\right\}$. Suppose that the Hash functions $H_{1},H_{2},H_{3}$ are collision resistant.

### 6.1. Message Confidentiality

Based on the security model defined in Definition 8 and Theorem 1, we can prove that our proposed scheme guarantees the message confidentiality under the hardness of the q-PBDHE assumption.

**Theorem** **1.** *If an adversary A can break $\left(T,q_{sk},q_{psk},q_{SC},q_{DS},\epsilon \right)$-IND-sEP-CCA2 security of our scheme, then there is an algorithm ℬ that can solve the q-PBDHE assumption with an advantage $\epsilon^{\prime }=\frac{1}{2}\epsilon -\frac{q_{DS}}{p}$ in a time $T^{\prime }=T+O\left(\ell_{e,m}n_{e,m}u_{m}+\left(n_{e,m}+\left|\tilde{U}\right|\ell_{e,m}n_{e,m}^{2}\right)q_{sk}+\left(\left|\tilde{U}\right|+\ell_{s,m}\right)q_{psk}+\left(\left|\tilde{U}\right|+l+\ell_{s,m}+\ell_{e,m}\right)q_{SC}+q_{DS}\right)T^{e}+O\left(q_{DS}\right)T^{p}$.*

**Proof.**  Assume A can $\left(T,q_{sk},q_{psk},q_{SC},q_{DS},\epsilon \right)$ break our scheme, we will construct the algorithm ℬ as follows: ℬ is given with the q-PBDHE challenge instance $\vec{Y}$. The challenger C runs $GG\left(1^{k}\right)\to \left(e,p,G,G_{T}\right)$ to generate the bilinear group and chooses 𝒷∈{0,1}. If 𝒷=0, C sends $\left(\vec{Y},\Omega =e{\left(g,g\right)}^{a^{q+1}w}\right)$ to ℬ; otherwise it sends $\left(\vec{Y},\Omega \overset{R}{\leftarrow }G_{T}\right)$ to ℬ.

Init. The same as defined in Definition 8. Assume ${ℛ}_{e}^{*}=\left(M_{e}^{*},\rho_{e}^{*}\right)$ is the challenge encryption access structure over all the attributes selected from the set of authorities $I_{A}^{*e}$. Assume $M_{e}^{*}$ is a $\ell_{e}^{*}\times n_{e}^{*}$ matrix and $n_{e}^{*}\leq q$.

Setup. The adversary chooses a set $S_{A}^{\prime }\subset S_{A}$ consisting of the corrupted authorities, and sends $S_{A}^{\prime }$ to the simulator ℬ. For each uncorrupted authority $AA_{k}\in S_{A}-S_{A}^{\prime }$, ℬ randomly chooses $\alpha_{k}^{\prime }\overset{R}{\leftarrow }{\mathbb{Z}}_{p}$ and implicitly sets $\alpha_{k}=\alpha_{k}^{\prime }+a^{q+1}$. ℬ publishes $\Delta_{k}=e{\left(g,g\right)}^{\alpha_{k}}=e\left(g^{a},g^{a^{q}}\right)e{\left(g,g\right)}^{\alpha_{k}^{\prime }}$.

Let $\psi \overset{R}{\leftarrow }{\mathbb{Z}}_{p},\theta =g^{a},\left\{{𝓀}_{0},{𝓀}_{1},\ldots ,{𝓀}_{l}\right\},\left\{v_{1},v_{2}\ldots ,v_{\ell_{s,m}}\right\}\overset{R}{\leftarrow }{\mathbb{Z}}_{p},{\left\{k_{i}=g^{{𝓀}_{i}}\right\}}_{i\in \left[l\right]},{\left\{V_{i}=g^{v_{i}}\right\}}_{i\in \left[\ell_{s,m}\right]}$. $\gamma_{1}=g^{\psi }{\left(g^{a^{q}}\right)}^{-1},\gamma_{2}={\left(g^{a^{q}}\right)}^{\frac{1}{\pi^{*}}}$, where $\pi^{*}=H_{1}\left(g^{w}\right)$.

ℬ sends $PP=\left\{e,p,G,G_{T},g,\theta ,\gamma_{1},\gamma_{2},\;\left\{k_{0},k_{1},\ldots ,k_{l}\right\},\left\{V_{1},V_{2}\ldots ,V_{\ell_{s,m}}\right\},H_{1},H_{2},H_{3}\right\}$ to A. ℬ initializes the empty list $L_{sk}$.

For the authority $AA_{k}\in S_{A}-S_{A}^{\prime }$, ℬ chooses $\beta_{k},\gamma_{k}\overset{R}{\leftarrow }{\mathbb{Z}}_{p}$ and sets $X_{k}=g^{1/\beta_{k}},Y_{k}=\theta^{1/\beta_{k}},Z_{k}=\theta^{1/\gamma_{k}}$. Let X be the set consisting of the indexes $i\in \left[\ell_{e}^{*}\right]$ with $\rho_{e}^{*}\left(i\right)=x\in \tilde{AA_{k}}$. For the attribute x where X≠∅, ℬ chooses $\varphi_{x}\overset{R}{\leftarrow }{\mathbb{Z}}_{p}$ and computes $A_{x}=g^{\varphi_{x}}\prod_{i\in X}\prod_{k\in \left[n_{e}^{*}\right]}g^{\frac{a^{k}M_{e}^{*\left(i,k\right)}}{b_{i}}}$, where $M_{e}^{*\left(i,k\right)}$ is the (i,k)th element of $M_{e}^{*}$. If X=∅, ℬ chooses $\varphi_{x}\overset{R}{\leftarrow }{\mathbb{Z}}_{p}$ and computes $A_{x}=g^{\varphi_{x}}$. This assignment describes that $A_{x}=g^{\varphi_{x}}$ for each signing attribute as the signing attributes are different from encryption attributes. ℬ sends $PK_{k}=\left\{X_{k},Y_{k},Z_{k},{\left\{A_{x}\right\}}_{x\in \tilde{AA_{k}}}\right\}$ to A. For the authority $AA_{k}\in S_{A}^{\prime }$, ℬ generates the public keys and secret keys of $AA_{k}$ as in the real scheme and sends both the public keys and secret keys to A.

Phase 1.

$SecretKey\;query\;O^{sk}\left(\tilde{U},AA_{k},uid\right)$. A adaptively queries the secret keys for the attribute set $\tilde{U}=\tilde{U^{d}}\cup \tilde{U^{s}}$ with identity uid to the authority $AA_{k}$. $\tilde{U^{d}}$ does not satisfy ${ℛ}_{e}^{*}$ together with any keys that can be obtained from corrupted authorities.

ℬ checks the list $L_{sk}$ that whether the entry $\left(uid,\tilde{U},PK_{uid},SK_{uid},PK_{uid,k},\;SK_{uid,k}\right)$ exists. If it does, ℬ sends $SK_{uid}$ and $SK_{uid,k}$ to the adversary and publishes the public key $PK_{uid}$ and $PK_{uid,k}$.

(1) Otherwise, ℬ randomly picks $d_{uid}^{\prime },s_{uid}^{\prime },z_{uid}$ from ${\mathbb{Z}}_{p}^{*}$ and chooses a vector $\vec{f}=\left(f_{1},f_{2},\ldots ,f_{n_{e}^{*}}\right)\in {\mathbb{Z}}_{p}^{n_{e}^{*}}$ such that f=−1 and $\vec{f}M_{e}^{*i}=0$ for all $\rho_{e}^{*}\left(i\right)\in \tilde{U^{d}}$ since ${ℛ}_{e}^{*}\left(\tilde{U^{d}}\right)=0$. ℬ sets $g^{d_{uid}}=g^{d_{uid}^{\prime }}\prod_{i=1}^{n_{e}^{*}}g^{\left(f_{i}a^{q-i+1}\right)/z_{uid}}$, $g^{s_{uid}}=g^{s_{uid}^{\prime }}g^{-a^{q}}$, and computes $g^{1/z_{uid}},\theta^{z_{uid}},g^{z_{uid}},{\left\{g^{s_{uid}v_{i}}\right\}}_{i\in \left[\ell_{s,m}\right]}$ as the public key $PK_{uid}$. Then ℬ computes

$PK_{uid,k}=\left\{PK_{uid,k}^{1}=g^{1/\left(\gamma_{k}z_{uid}\right)},PK_{uid,k}^{2}=g^{\alpha_{k}/\left(\gamma_{k}z_{uid}\right)}\theta^{d_{uid}/\gamma_{k}}={\left(g^{\frac{\alpha_{k}^{\prime }}{z_{uid}}}g^{ad_{uid}^{\prime }}g^{\sum_{2}^{n_{e}^{*}}f_{i}a^{q-i+2}/z_{uid}}\right)}^{\frac{1}{\gamma_{k}}},PK_{uid,k}^{3}=g^{\alpha_{k}/\beta_{k}}\theta^{s_{uid}/\beta_{k}}={\left(g^{\alpha_{k}^{\prime }}g^{as_{uid}^{\prime }}\right)}^{\frac{1}{\beta_{k}}}\right\}$, and sets $SK_{uid,k}$ as $K_{uid,k}^{d}={\left(PK_{uid,k}^{2}\right)}^{\gamma_{k}}=g^{\frac{\alpha_{k}^{\prime }}{z_{uid}}}g^{ad_{uid}^{\prime }}g^{\sum_{2}^{n_{e}^{*}}f_{i}a^{q-i+2}/z_{uid}},K_{uid,k}^{s}={\left(PK_{uid,k}^{3}\right)}^{\beta_{k}}=g^{\alpha_{k}^{\prime }}g^{as_{uid}^{\prime }},{\left\{F_{uid,x}^{s}={\left(g^{s_{uid}^{\prime }}g^{-a^{q}}\right)}^{\varphi_{x}}\right\}}_{x\in \tilde{U^{s}}\cap \tilde{AA_{k}}}$. For the attribute $x\in \tilde{U^{d}}\cap \tilde{AA_{k}}$ such that X=∅, ℬ computes $F_{uid,x}^{d}={\left(g^{d_{uid}}\right)}^{\varphi_{x}}$. Otherwise, $F_{uid,x}^{d}=g^{d_{uid}\varphi_{x}}\prod_{i\in X}\prod_{k\in \left[n_{e}^{*}\right]}{\left(g^{\frac{d_{uid}^{\prime }a^{k}}{b_{i}}}\prod_{j=1,k\neq j}^{n_{e}^{*}}g^{\frac{f_{j}a^{q+k-j+1}}{z_{uid}b_{i}}}\right)}^{M_{e}^{*\left(i,k\right)}}$. ℬ sends $SK_{uid}=z_{uid}$ and $SK_{uid,k}$ to the adversary and publishes the public key $PK_{uid}$ and $PK_{uid,k}$. ℬ inserts $\left(uid,\tilde{U},PK_{uid},SK_{uid},PK_{uid,k},\;SK_{uid,k}\right)$ into $L_{sk}$.

$Proxy\;SecretKey\;query\;O^{psk}\left(\tilde{U},AA_{k},uid\right)$. ℬ checks the list $L_{sk}$ that whether the entry $\left(uid,\tilde{U},PK_{uid},SK_{uid},PK_{uid,k},\;SK_{uid,k}\right)$ exists. If it does not exist, ℬ issues $O^{sk}\left(\tilde{U},AA_{k},uid\right)$ query to compute $SK_{uid}$ and $SK_{uid,k}$, and then runs $PxSecretKeyGen\left(SK_{uid},SK_{uid,k}\right)$ and returns $PSK_{uid,k}$ to A. Otherwise, ℬ directly performs $PxSecretKeyGen\left(SK_{uid},SK_{uid,k}\right)$ and returns $PSK_{uid,k}$ to A.

$Signcryption\;query\;O^{SC}\left(ℳ,{ℛ}_{s},{ℛ}_{e}\right)$. A submits a message $ℳ\in G_{T}$, signing and encryption predicts ${ℛ}_{s}=\left(M_{s},\rho_{s}\right),{ℛ}_{e}=\left(M_{e},\rho_{e}\right)$. ℬ selects a signing attribute set $\tilde{U^{s}}$ such that ${ℛ}_{s}\left(\tilde{U^{s}}\right)=1$. For each $k\in I_{A}^{s}$, ℬ computes the secret signing key $SK_{uid,k}^{s}$ and $PK_{uid},SK_{uid}$ from $O^{sk}\left(\tilde{U},AA_{k},uid\right)$, and $PSK_{uid,k}^{s}\leftarrow PxSecretKeyGen\left(SK_{uid},SK_{uid,k}^{s}\right)$, where uid is an arbitrary identity. Then ℬ returns the ciphertext $CT\leftarrow Signcryption\left(ℳ,PP,{\left\{PSK_{uid,k}^{s}\right\}}_{k\in I_{A}^{s}},PK_{uid},{\left\{PK_{k}\right\}}_{k\in I_{A}^{e}},SK_{uid},{ℛ}_{s},{ℛ}_{e}\right)$ to A.

$DeSigncryption\;query\;O^{DS}\left(CT,\tilde{U^{d}}\right)$. If $\left|tt-\bar{tt}\right|>thre_{tt}$ or ${ℛ}_{e}\left(\tilde{U^{d}}\right)=0$, then ℬ returns ⊥. If $C_{1}=g^{s}$, ℬ aborts. If Verify algorithm is invalid, ℬ returns ⊥.Otherwise, ℬ carries out the following steps.

Assume the encryption predicate contained in CT is ${ℛ}_{e}$ and $I_{A}^{e}$ is the set which consists of the indexes of the authorities whose attributes are associated with rows of $M_{e}$.

If $\tilde{U^{d}}$ does not satisfy the challenge encryption predicate ${ℛ}_{e}^{*}$, then ℬ can obtain $SK_{uid}$ and secret decryption key $SK_{uid,k}^{d}$ from $O^{sk}\left(\tilde{U},AA_{k},uid\right)$, and $PSK_{uid,k}^{d}\leftarrow PxSecretKeyGen\left(SK_{uid},SK_{uid,k}^{d}\right)$. ℬ returns the output of $DeSigncryption\left(PP,CT,PK_{uid},{\left\{PSK_{uid,k}^{d}\right\}}_{k\in I_{A}},SK_{uid}\right)$ to A.

Otherwise, if ${ℛ}_{e}^{*}\left(\tilde{U^{d}}\;\right)=1$, assume $\pi =H_{1}\left(C_{1}=g^{w_{1}}\right)$, where $w_{1}$ is the secret value chosen to generate CT in signcryption phase. Then for $k\in I_{A}^{e}$, ℬ compute $e\left(g^{\alpha_{k}^{\prime }},C_{1}\right)e{\left(\frac{C_{3}}{C_{1}^{\psi }},g^{a}\right)}^{{\left(\frac{\pi }{\pi^{*}}-1\right)}^{-1}}=e\left(g^{\alpha_{k}^{\prime }},g^{w_{1}}\right)e{\left(\frac{{\left(\gamma_{1}\gamma_{2}{}^{\pi }\right)}^{w_{1}}}{g^{s_{x}\psi }},g^{a}\right)}^{{\left(\frac{\pi }{\pi^{*}}-1\right)}^{-1}}=e\left(g^{\alpha_{k}^{\prime }},g^{w_{1}}\right)e{\left(\frac{{\left(g^{\psi }{\left(g^{a^{q}}\right)}^{-1}{\left(g^{a^{q}}\right)}^{\frac{\pi }{\pi^{*}}}\right)}^{w_{1}}}{g^{w_{1}\psi }},g^{a}\right)}^{{\left(\frac{\pi_{j}}{\pi^{*}}-1\right)}^{-1}}=e\left(g^{\alpha_{k}^{\prime }},g^{w_{1}}\right)e\left(g^{a^{q}},g^{aw_{1}}\right)=\Delta_{k}^{w_{1}}$. Thus ℬ can return $ℳ=\frac{C_{0}}{\prod_{k\in I_{A}^{e}}\Delta_{k}^{w_{1}}}$ to A.

Challenge. A submits two messages ${ℳ}_{0},{ℳ}_{1}$ with the same length and signing predicate ${ℛ}_{s}^{*}=\left(M_{s}^{*},\rho_{s}^{*}\right)$ to ℬ. Assume $I_{A}^{*s}$ is the set which consists of the indexes of the authorities whose attributes are associated with rows of $M_{s}^{*}$ and $M_{s}^{*}$ is a $\ell_{s}^{*}\times n_{s}^{*}$ matrix. ℬ chooses $\hat{𝒷}\in \left\{0,1\right\}$. ℬ selects a signing attribute set $\tilde{U^{s}}$ satisfying ${ℛ}_{s}^{*}\left(\tilde{U^{s}}\right)=1$ and an arbitrary identity $uid_{A}$.

Let $\vec{a}=\left(a_{1},a_{2},\ldots ,a_{\ell_{s}^{*}}\right)\in {\mathbb{Z}}_{p}^{\ell_{s}^{*}}$ such that $\vec{a}\cdot M_{s}^{*}=\vec{1}$, $\vec{b}=\left(b_{1},b_{2},\ldots ,b_{\ell_{s}^{*}}\right)\in {\mathbb{Z}}_{p}^{\ell_{s}^{*}}$ such that $\sum_{i\in \left[\ell_{s}^{*}\right]}b_{i}M_{s}^{*i}=\vec{0}$. Implicitly set $s_{uid_{A}}=s_{uid_{A}}^{\prime \prime }-a^{q}$. Then compute the challenge ciphertext as follows:

Let $\vec{\varepsilon }=\left(w,wa+\varepsilon_{2},\ldots ,sa^{n_{e}^{*}-1}+\varepsilon_{n_{e}^{*}}\right)\in {\mathbb{Z}}_{p}^{n_{e}^{*}}$ and implicitly sets $r_{i}=r_{i}^{\prime }+wb_{i}$ for all $i\in \left[\ell_{e}^{*}\right]$. Select $\left\{r_{1}^{\prime \prime },r_{2}^{\prime \prime },\ldots ,r_{\ell_{e}^{*}}^{\prime \prime }\right\}\overset{R}{\leftarrow }{\mathbb{Z}}_{p}$, $\left\{\lambda_{1}^{\prime },\lambda_{2}^{\prime },\ldots ,\lambda_{\ell_{e}^{*}}^{\prime }\right\}\overset{R}{\leftarrow }{\mathbb{Z}}_{p}$.

$C_{0}={ℳ}_{\hat{𝒷}}\prod_{k\in I_{A}^{*e}}\Omega e{\left(g^{w},g\right)}^{\alpha_{k}^{\prime }}$, $C_{1}=g^{w}$.

$C_{2,i}^{\prime }=g^{a\left(\lambda_{i}-\lambda_{i}^{\prime }\right)}A_{\rho_{e}^{*}\left(i\right)}{}^{-\left(r_{i}-r_{i}^{\prime \prime }\right)}=A_{\rho_{e}^{*}\left(i\right)}{}^{-\left(r_{i}^{\prime }-r_{i}^{\prime \prime }\right)}g^{-wb_{i}\varphi_{\rho_{e}^{*}\left(i\right)}}g^{-a\lambda_{i}^{\prime }}\prod_{k=2}^{n_{e}^{*}}g^{a\varepsilon_{k}M_{e}^{*\left(i,k\right)}}\prod_{l\in X\backslash i}\prod_{k\in \left[n_{e}^{*}\right]}g^{\frac{-wa^{k}b_{i}M_{e}^{*\left(l,k\right)}}{b_{l}}}$, $C_{2,i}^{\prime \prime }=\lambda_{i}^{\prime }$.

$D_{i}^{\prime }=g^{r_{i}-r_{i}^{\prime \prime }}=g^{r_{i}^{\prime }-r_{i}^{\prime \prime }}g^{wb_{i}}$, $D_{i}^{\prime \prime }=r_{i}^{\prime \prime }$. $C_{3}=g^{\psi w}$.

$S_{1,i}=g^{a_{i}s_{uid_{A}}+b_{i}}=g^{a_{i}\left(s_{uid_{A}}^{\prime \prime }-a^{q}\right)+b_{i}}$,

$S_{2}=\left(\prod_{I_{A}^{*s}}g^{\alpha_{k}^{\prime }}g^{as_{uid_{A}}^{\prime \prime }}\right)\left(\prod_{i\in \left[\ell_{s}\right]}{\left(g^{\varphi_{\rho_{s}^{*}\left(i\right)}+v_{i}}\right)}^{a_{i}\left(s_{uid_{A}}^{\prime \prime }-a^{q}\right)+b_{i}}\right){\left(g^{w}\right)}^{{𝓀}_{0}+\sum_{i=1}^{l}{𝓀}_{i}c_{i}+\psi \beta^{*}}$, where $H_{2}\left(\prod_{i\in \left[\ell_{s}\right]}S_{1,i},tt,{ℛ}_{s},{ℛ}_{e}\right)=\left(c_{1},c_{2},\ldots ,c_{l}\right)$ and $H_{3}\left(C_{0},C_{1},C_{3},{ℛ}_{s},{ℛ}_{e}\right)=\beta^{*}$.

Finally, ℬ sends the challenge ciphertext $CT^{*}=\left\{C_{0},C_{1},{\left\{C_{2,i}^{\prime },C_{2,i}^{\prime \prime },D_{i}^{\prime },D_{i}^{\prime \prime }\right\}}_{i\in \left[\ell_{e}^{*}\right]},{\left\{S_{1,i}\right\}}_{i\in \left[\ell_{s}^{*}\right]},S_{2},tt\right\}$ to A.

Phase 2. Phase 1 is repeated. In this phase, A cannot issue DeSigncryption query with the challenge ciphertext $CT^{*}$ and attribute set $\tilde{U_{d}}$ such that ${ℛ}_{e}^{*}\left(\tilde{U^{d}}\;\right)=1$.

Guess. A outputs his guess $\tilde{𝒷}$ on $\hat{𝒷}$. If $\tilde{𝒷}=\hat{𝒷}$, ℬ outputs 0 and guess that $\Omega =e{\left(g,g\right)}^{a^{q+1}w}$; otherwise, ℬ outputs 1 to indicate that Ω is a random element in $G_{T}$.

If A issues DeSigncryption query with the ciphertext satisfying $C_{1}=g^{w}$, then the simulation aborts. The probability is at most $\frac{q_{DS}}{p}$. If 𝒷=0, $\Omega =e{\left(g,g\right)}^{a^{q+1}w}$ and ℬ does not abort, then $CT^{*}$ is a valid ciphertext of ${ℳ}_{0}$. In this case, we have $Pr\left[\tilde{𝒷}=\hat{𝒷}|𝒷=0\right]>\frac{1}{2}+\epsilon -\frac{q_{DS}}{p}$. If Ω is a random element in $G_{T}$, then $C_{0}$ is a random element and A cannot obtain ${ℳ}_{\hat{𝒷}}$, namely the advantage in this case is $Pr\left[\tilde{𝒷}\neq \hat{𝒷}|𝒷=1\right]=\frac{1}{2}$. Therefore, the advantage of ℬ which can break the q-PBDHE assumption is at least $\frac{1}{2}\epsilon -\frac{q_{DS}}{p}$. The runtime of ℬ is at most $T^{\prime }=T+O\left(\ell_{e,m}n_{e,m}u_{m}+\left(n_{e,m}+\left|\tilde{U}\right|\ell_{e,m}n_{e,m}^{2}\right)q_{sk}+\left(\left|\tilde{U}\right|+\ell_{s,m}\right)q_{psk}+\left(\left|\tilde{U}\right|+l+\ell_{s,m}+\ell_{e,m}\right)q_{SC}+q_{DS}\right)T^{e}+O\left(q_{DS}\right)T^{p}$. □

### 6.2. Ciphertext Unforgeability

Based on the security model defined in Definition 9 and Theorem 2, we can prove that our proposed scheme guarantees the ciphertext unforgeability under the hardness of the q-PBDHE assumption.

**Theorem** **2.** *If an adversary A can break $\left(T,q_{sk},q_{psk},q_{SC},q_{DS},\epsilon \right)$-EUF-sSP-CMA security of our scheme, then there is an algorithm ℬ that can solve the q-PBDHE assumption with an advantage $\epsilon^{\prime }=\frac{\epsilon }{8\left(l+1\right)q_{SC}}$ in a time $T^{\prime }=T+O\left(\ell_{s,m}n_{s,m}u_{m}+\left(n_{s,m}+\left|\tilde{U}\right|\ell_{s,m}n_{s,m}^{2}\right)q_{sk}+\left(\left|\tilde{U}\right|+\ell_{s,m}\right)q_{psk}+\left(l+\ell_{e,m}+\ell_{s,m}+\ell_{e,m}n_{e,m}\right)q_{SC}+\ell_{e,m}q_{DS}\right)T^{e}+O\left(\ell_{e,m}q_{DS}\right)T^{p}$.*

**Proof.**  Assume A can $\left(T,q_{sk},q_{psk},q_{SC},q_{DS},\epsilon \right)$ break our basic scheme, we will construct the algorithm ℬ as follows: ℬ is given with the q-PBDHE challenge instance $\vec{Y}$. The challenger C runs $GG\left(1^{k}\right)\to \left(e,p,G,G_{T}\right)$ to generate the bilinear group and chooses 𝒷∈{0,1}. If 𝒷=0, C sends $\left(\vec{Y},\Omega =e{\left(g,g\right)}^{a^{q+1}w}\right)$ to ℬ; otherwise it sends $\left(\vec{Y},\Omega \overset{R}{\leftarrow }G_{T}\right)$ to ℬ.

Init. The same as defined in Definition 9. Assume ${ℛ}_{s}^{*}=\left(M_{s}^{*},\rho_{s}^{*}\right)$ is the challenge signing access structure over all the attributes selected from the involved set of authorities $I_{A}^{*s}$. $M_{s}^{*}$ is a $\ell_{s}^{*}\times n_{s}^{*}$ matrix and $n_{s}^{*}\leq q$.

Setup. The adversary chooses a set of $S_{A}^{\prime }\subset S_{A}$ consisting of the corrupted authorities, and sends $S_{A}^{\prime }$ to the simulator ℬ.

For each uncorrupted authority $AA_{k}\in S_{A}-S_{A}^{\prime }$, ℬ randomly chooses $\alpha_{k}^{\prime }\overset{R}{\leftarrow }{\mathbb{Z}}_{p}$ and implicitly sets $\alpha_{k}=\alpha_{k}^{\prime }+a^{q+1}$. ℬ publishes $\Delta_{k}=e{\left(g,g\right)}^{\alpha_{k}}=e\left(g^{a},g^{a^{q}}\right)e{\left(g,g\right)}^{\alpha_{k}^{\prime }}$.

Let $\sigma_{1},\sigma_{2}\overset{R}{\leftarrow }{\mathbb{Z}}_{p},\theta =g^{a}$. ℬ chooses $m\overset{R}{\leftarrow }\left\{0,1,\ldots ,l\right\},\varrho_{0},\varrho_{1},\ldots ,\varrho_{l}\overset{R}{\leftarrow }\left\{0,1,\ldots ,\varpi -1\right\},\sigma_{1},\sigma_{2},{𝓀}_{0},{𝓀}_{1},\ldots ,{𝓀}_{l}\overset{R}{\leftarrow }{\mathbb{Z}}_{p}^{*}$. Set $k_{0}={\left(g^{a^{q}}\right)}^{\varrho_{0}-\varpi m}g^{{𝓀}_{0}}$ and ${\left\{k_{i}={\left(g^{a^{q}}\right)}^{\varrho_{i}}g^{{𝓀}_{i}}\right\}}_{i\in \left[l\right]}$. $\gamma_{1}=g^{\sigma_{1}},\gamma_{2}=g^{\sigma_{2}}$. For $i\in \left[\ell_{s}^{*}\right]$, $V_{i}=\prod_{l\in X\backslash i}\prod_{k\in \left[n_{s}^{*}\right]}g^{a^{k}M_{s}^{*\left(l,k\right)}N_{A}^{*s}}$ where $N_{A}^{*s}=\left|I_{A}^{*s}\right|$. For $i\in \left[\ell_{s}^{*}+1,\ell_{s,m}\right]$, $V_{i}=g^{v_{i}}$ where $v_{i}\overset{R}{\leftarrow }{\mathbb{Z}}_{p}$.

Assume $\varpi =4q_{SC}$ and ϖ(l+1)<p. ℬ defines two functions $L_{1}\left(\vec{c}\right)=p-\varpi m+\varrho_{0}+\sum_{i=1}^{l}c_{i}\varrho_{i}$ and $L_{2}\left(\vec{c}\right)={𝒷}_{0}+\sum_{i=1}^{l}c_{i}{𝒷}_{i}$ for each $\vec{c}=\left(c_{1},c_{2},\ldots ,c_{l}\right)\in {\left\{0,1\right\}}^{l}$. Thus $k_{0}\prod_{i=1}^{l}k_{i}{}^{c_{i}}={\left(g^{a^{q}}\right)}^{L_{1}\left(\vec{c}\right)}g^{L_{2}\left(\vec{c}\right)}$. Let $L\left(\vec{c}\right)=\{\begin{array}{c} 0,\;\varrho_{0}+\sum_{i=1}^{l}c_{i}\varrho_{i}=0\;mod\;\varpi \;\\ 1,\;otherwise \end{array}$. Then $L\left(\vec{c}\right)=1$ implies $L_{1}\left(\vec{c}\right)\neq 0\;mod\;p$.

ℬ sends $PP=\left\{e,p,G,G_{T},g,\theta ,\gamma_{1},\gamma_{2},\;\left\{k_{0},k_{1},\ldots ,k_{l}\right\},\left\{V_{1},V_{2}\ldots ,V_{\ell_{s,m}}\right\},H_{1},H_{2},H_{3}\right\}$ to A. ℬ initializes the empty list $L_{sk}$.

For the authority $AA_{k}\in S_{A}-S_{A}^{\prime }$, ℬ chooses $\beta_{k},\gamma_{k}\overset{R}{\leftarrow }{\mathbb{Z}}_{p}$ and sets $X_{k}=g^{1/\beta_{k}},Y_{k}=\theta^{1/\beta_{k}},Z_{k}=\theta^{1/\gamma_{k}}$. Let X be the set consisting of the indexes $i\in \left[\ell_{s}^{*}\right]$ with $\rho_{s}^{*}\left(i\right)=x\in \tilde{AA_{k}}$. For the attribute x where X≠∅, ℬ chooses $\varphi_{x}\overset{R}{\leftarrow }{\mathbb{Z}}_{p}$ and computes $A_{x}=g^{\varphi_{x}}\prod_{i\in X}\prod_{k\in \left[n_{s}^{*}\right]}g^{-a^{k}M_{s}^{*\left(i,k\right)}N_{A}^{*s}}$. If X=∅, ℬ chooses $\varphi_{x}\overset{R}{\leftarrow }{\mathbb{Z}}_{p}$ and computes $A_{x}=g^{\varphi_{x}}$. This assignment describes that $A_{x}=g^{\varphi_{x}}$ for each encryption attribute as the signing attributes are different from encryption attributes. ℬ sends $PK_{k}=\left\{X_{k},Y_{k},Z_{k},{\left\{A_{x}\right\}}_{x\in \tilde{AA_{k}}}\right\}$ to A. For the authority $AA_{k}\in S_{A}^{\prime }$, ℬ generates the public keys and secret keys of $AA_{k}$ as in the real scheme and sends both the public keys and secret keys to A.

$SecretKey\;query\;O^{sk}\left(\tilde{U},AA_{k},uid\right)$. A adaptively queries the secret keys for the attribute set $\tilde{U}=\tilde{U^{d}}\cup \tilde{U^{s}}$ with identity uid to the authority $AA_{k}$. $\tilde{U^{s}}$ does not satisfy ${ℛ}_{s}^{*}$ together with any keys that can be obtained from corrupted authorities.

(1) ℬ checks the list $L_{sk}$ that whether the entry $\left(uid,\tilde{U},PK_{uid},SK_{uid},PK_{uid,k},\;SK_{uid,k}\right)$ exists. If it does, ℬ sends $SK_{uid}$ and $SK_{uid,k}$ to the adversary and publishes the public key $PK_{uid}$ and $PK_{uid,k}$.
(2) Otherwise, ℬ randomly picks $d_{uid}^{\prime },s_{uid}^{\prime },z_{uid}$ from ${\mathbb{Z}}_{p}^{*}$ and chooses a vector $\vec{f}=\left(f_{1},f_{2},\ldots ,f_{n_{s}^{*}}\right)\in {\mathbb{Z}}_{p}^{n_{s}^{*}}$ such that f=−1 and $\vec{f}M_{s}^{*i}=0$ for all $\rho_{s}^{*}\left(i\right)\in \tilde{U^{s}}$. since ${ℛ}_{s}^{*}\left(\tilde{U^{s}}\right)=0$. ℬ computes $g^{d_{uid}}=g^{d_{uid}^{\prime }}g^{-a^{q}/z_{uid}}$, $g^{s_{uid}}=g^{s_{uid}^{\prime }}\prod_{i=1}^{n_{s}^{*}}g^{f_{i}a^{q-i+1}}$, and ${\left\{V_{i}^{s_{uid}}=g^{s_{uid}v_{i}}\right\}}_{i\in \left[\ell_{s}^{*}+1,\ell_{s,m}\right]}$. For $i\in \left[\ell_{s}^{*}\right]$, $V_{i}^{s_{uid}}=V_{i}^{s_{uid}^{\prime }}\prod_{l\in Xi}\prod_{k\in \left[n_{s}^{*}\right]}{\left(\prod_{j=1,k\neq j}^{n_{s}^{*}}g^{f_{j}a^{q+k-j+1}}\right)}^{M_{s}^{*\left(l,k\right)}N_{A}^{*s}}$. Set $PK_{uid}=\left\{g^{s_{uid}},g^{d_{uid}},g^{1/z_{uid}},\theta^{z_{uid}},g^{z_{uid}},{\left\{V_{i}^{s_{uid}}\right\}}_{i\in \left[\ell_{s,m}\right]}\right\}$ and $PK_{uid,k}=\left\{PK_{uid,k}^{1}=g^{1/\left(\gamma_{k}z_{uid}\right)},PK_{uid,k}^{2}=g^{\alpha_{k}/\left(\gamma_{k}z_{uid}\right)}\theta^{d_{uid}/\gamma_{k}}={\left(g^{\frac{\alpha_{k}^{\prime }}{z_{uid}}}g^{ad_{uid}^{\prime }}\right)}^{\frac{1}{\gamma_{k}}},PK_{uid,k}^{3}=g^{\alpha_{k}/\beta_{k}}\theta^{s_{uid}/\beta_{k}}={\left(g^{\alpha_{k}^{\prime }}g^{as_{uid}^{\prime }}g^{\sum_{2}^{n_{s}^{*}}f_{i}a^{q-i+2}}\right)}^{\frac{1}{\beta_{k}}}\right\}$. Then ℬ sets . For the attribute $x\in \tilde{U^{s}}\cap \tilde{AA_{k}}$ such that X=∅, ℬ computes $F_{uid,x}^{s}={\left(g^{s_{uid}}\right)}^{\varphi_{x}}$. Otherwise, $F_{uid,x}^{s}=g^{s_{uid}\varphi_{x}}\prod_{i\in X}\prod_{k\in \left[n_{s}^{*}\right]}{\left(g^{-s_{uid}^{\prime }a^{k}}\prod_{j=1,k\neq j}^{n_{s}^{*}}g^{-f_{j}a^{q+k-j+1}}\right)}^{M_{s}^{*\left(i,k\right)}N_{A}^{s}}$. ℬ sends $SK_{uid}=z_{uid}$ and $SK_{uid,k}$ to the adversary and publishes the public key $PK_{uid}$ and $PK_{uid,k}$. ℬ inserts $\left(uid,\tilde{U},PK_{uid},SK_{uid},PK_{uid,k},\;SK_{uid,k}\right)$ into $L_{sk}$.

$Proxy\;SecretKey\;query\;O^{psk}\left(\tilde{U},AA_{k},uid\right)$. The same as Theorem 1.

$Signcryption\;query\;O^{SC}\left(ℳ,{ℛ}_{s},{ℛ}_{e}\right)$. A submits a message $ℳ\in G_{T}$, signing and encryption predicts ${ℛ}_{s}=\left(M_{s},\rho_{s}\right),{ℛ}_{e}=\left(M_{e},\rho_{e}\right)$. ℬ selects a signing attribute set $\tilde{U^{s}}$ such that ${ℛ}_{s}\left(\tilde{U^{s}}\right)=1$. ℬ performs as follows:

(1) It first computes a vector $\vec{a}=\left(a_{1},a_{2},\ldots ,a_{\ell_{s}}\right)\in {\mathbb{Z}}_{p}^{\ell_{s}}$ such that $\vec{a}\cdot M_{s}=\vec{1}$. Then ℬ chooses $\vec{b}=\left(b_{1},b_{2},\ldots ,b_{\ell_{s}}\right)\in {\mathbb{Z}}_{p}^{\ell_{s}}$ such that $\sum_{i\in \left[\ell_{s}\right]}b_{i}M_{s}^{i}=\vec{0}$.
(2) ℬ randomly chooses $s_{uid}^{\prime }\overset{R}{\leftarrow }{\mathbb{Z}}_{p}^{*}$ and computes ${\left\{S_{1,i}=g^{a_{i}s_{uid}^{\prime }+b_{i}}\right\}}_{i\in \left[\ell_{s}\right]}$.
(3) Assume $H_{2}\left(\prod_{i\in \left[\ell_{s}\right]}S_{1,i},tt,{ℛ}_{s},{ℛ}_{e}\right)=\left(c_{1},c_{2},\ldots ,c_{l}\right)=\vec{c}\in {\left\{0,1\right\}}^{l}$. If $L\left(\vec{c}\right)=0$, ℬ aborts. Otherwise, ℬ implicitly sets $w_{A}=w_{1}-\frac{aN_{A}^{s}}{L_{1}\left(\vec{c}\right)}$ where $w_{1}\overset{R}{\leftarrow }{\mathbb{Z}}_{p}^{*}$. Then $C_{0}=ℳ\prod_{k\in I_{A}^{e}}\Delta_{k}{}^{w_{A}}$, $C_{1}=g^{w_{A}}=g^{w_{1}}{\left(g^{a}\right)}^{-\frac{N_{A}^{s}}{L_{1}\left(\vec{c}\right)}}$, $C_{3}={\left(g^{\sigma_{1}}g^{\pi \sigma_{2}}\right)}^{w_{A}}$, where $\Delta_{k}{}^{w_{A}}=\Delta_{k}{}^{w_{1}}{\left(e\left(g^{a^{2}},g^{a^{q}}\right)e\left(g^{\alpha_{k}^{\prime }},g^{a}\right)\right)}^{-N_{A}^{s}/L_{1}\left(\vec{c}\right)}$ and $\pi =H_{1}\left(C_{1}\right)$.
(4) ℬ chooses $\left\{r_{1},r_{2},\ldots ,r_{\ell_{e}}\right\}\overset{R}{\leftarrow }{\mathbb{Z}}_{p}$, $\vec{\varepsilon }=\left(w_{1}-\frac{aN_{A}^{s}}{L_{1}\left(\vec{c}\right)},\varepsilon_{2},\ldots ,\varepsilon_{n_{e}}\right)\in {\mathbb{Z}}_{p}^{n_{e}}$, $\lambda_{i}=M_{e}^{i}\vec{\varepsilon }$. Then ℬ selects $\left\{r_{1}^{\prime },r_{2}^{\prime },\ldots ,r_{\ell_{e}}^{\prime }\right\}\overset{R}{\leftarrow }{\mathbb{Z}}_{p}$, $\left\{\lambda_{1}^{\prime },\lambda_{2}^{\prime },\ldots ,\lambda_{\ell_{e}}^{\prime }\right\}\overset{R}{\leftarrow }{\mathbb{Z}}_{p}$. For $i\in \left[\ell_{e}\right]$, ℬ computes $C_{2,i}^{\prime }=g^{a\left(\lambda_{i}-\lambda_{i}^{\prime }\right)}A_{\rho_{e}\left(i\right)}{}^{-\left(r_{i}-r_{i}^{\prime }\right)}=g^{a\left(\left(w_{1}-\frac{aN_{A}^{s}}{L_{1}\left(\vec{c}\right)}\right)M_{e}^{\left(i,1\right)}+\sum_{j=2}^{n_{e}^{*}}\varepsilon_{j}M_{e}^{\left(i,j\right)}-\lambda_{i}^{\prime }\right)}g^{-\varphi_{\rho_{e}\left(i\right)}\left(r_{i}-r_{i}^{\prime }\right)}$, $C_{2,i}^{\prime \prime }=\lambda_{i}^{\prime }$, $D_{i}^{\prime }=g^{r_{i}-r_{i}^{\prime }}$, $D_{i}^{\prime \prime }=r_{i}^{\prime }$.
(5) ℬ computes $H_{3}\left(C_{0},C_{1},C_{3},{ℛ}_{s},{ℛ}_{e}\right)=\beta$, $S_{2}=\left(\prod_{I_{A}^{s}}g^{\alpha_{k}}\right)\theta^{s_{uid}^{\prime }N_{A}^{s}}\left(\prod_{i\in \left[\ell_{s}\right]}{\left(A_{\rho_{s}\left(i\right)}V_{i}\right)}^{a_{i}s_{uid}^{\prime }+b_{i}}\right){\left(k_{0}\prod_{i=1}^{l}k_{i}{}^{c_{i}}\right)}^{w_{A}}{\left(\gamma_{1}\gamma_{2}{}^{\pi }\right)}^{w_{A}\beta }=\left(\prod_{I_{A}^{s}}g^{\alpha_{k}^{\prime }+a^{q+1}}\right)\theta^{s_{uid}^{\prime }N_{A}^{s}}C_{3}^{\beta }{\left({\left(g^{a^{q}}\right)}^{L_{1}\left(\vec{c}\right)}g^{L_{2}\left(\vec{c}\right)}\right)}^{w_{1}-\frac{aN_{A}^{s}}{L_{1}\left(\vec{c}\right)}}\left(\prod_{i=1}^{\ell_{s}}{\left(A_{\rho_{s}\left(i\right)}V_{i}\right)}^{a_{i}s_{uid}^{\prime }+b_{i}}\right)=\left(\prod_{I_{A}^{s}}g^{\alpha_{k}^{\prime }}\right)g^{a^{q+1}N_{A}^{s}}\theta^{s_{uid}^{\prime }N_{A}^{s}}C_{3}^{\beta }{\left({\left(g^{a^{q}}\right)}^{L_{1}\left(\vec{c}\right)}g^{L_{2}\left(\vec{c}\right)}\right)}^{w_{1}}{\left(g^{aN_{A}^{s}}\right)}^{\frac{-L_{2}\left(\vec{c}\right)}{L_{1}\left(\vec{c}\right)}}g^{-a^{q+1}N_{A}^{s}}\left(\prod_{i=1}^{\ell_{s}}{\left(A_{\rho_{s}\left(i\right)}V_{i}\right)}^{a_{i}s_{uid}^{\prime }+b_{i}}\right)=\left(\prod_{I_{A}^{s}}g^{\alpha_{k}^{\prime }}\right)\theta^{s_{uid}^{\prime }N_{A}^{s}}C_{3}^{\beta }{\left({\left(g^{a^{q}}\right)}^{L_{1}\left(\vec{c}\right)}g^{L_{2}\left(\vec{c}\right)}\right)}^{w_{1}}{\left(g^{aN_{A}^{s}}\right)}^{\frac{-L_{2}\left(\vec{c}\right)}{L_{1}\left(\vec{c}\right)}}\left(\prod_{i=1}^{\ell_{s}}{\left(A_{\rho_{s}\left(i\right)}V_{i}\right)}^{a_{i}s_{uid}^{\prime }+b_{i}}\right)$. Finally, ℬ sends $CT=\left\{C_{0},C_{1},{\left\{C_{2,i}^{\prime },C_{2,i}^{\prime \prime },D_{i}^{\prime },D_{i}^{\prime \prime }\right\}}_{i\in \left[\ell_{e}\right]},{\left\{S_{1,i}\right\}}_{i\in \left[\ell_{s}\right]},S_{2},tt\right\}$ to A.

$DeSigncryption\;query\;O^{DS}\left(CT,\tilde{U^{d}}\right)$. If $\left|tt-\bar{tt}\right|>thre_{tt}$ or ${ℛ}_{e}\left(\tilde{U^{d}}\right)=0$, then ℬ returns ⊥. Otherwise, ℬ issues the $O^{\mathrm{sk}}$ query to get the secret decryption key and returns the output of DeSigncryption to A.

Forgery. A submits a valid ciphertext $CT^{*}$ for the challenge signing predicate ${ℛ}_{s}^{*}$ and an encryption predicate ${ℛ}_{e}$. If $ℳ\leftarrow DeSigncryption\left(PP,CT^{*},PK,SK\right)$ and A has never issued $O^{\mathrm{SC}}\left(ℳ,{ℛ}_{s}^{*},{ℛ}_{e}\right)$. ℬ performs as follows:

(1) ℬ computes $H_{2}\left(\prod_{i\in \left[\ell_{s}^{*}\right]}S_{1,i},tt,{ℛ}_{s}^{*},{ℛ}_{e}\right)=\left(c_{1},c_{2},\ldots ,c_{l}\right)=\vec{c}\in {\left\{0,1\right\}}^{l}$. If $\varpi m\neq \varrho_{0}+\sum_{i=1}^{l}c_{i}\varrho_{i}$, ℬ aborts. Otherwise, $L_{1}\left(\vec{c}\right)=0\;mod\;p$.
(2) If $CT^{*}$ is a valid ciphertext, then $H_{3}\left(C_{0},C_{1},C_{3},{ℛ}_{s}^{*},{ℛ}_{e}\right)=\beta$ and $\pi =H_{1}\left(C_{1}\right)$. Then

$S_{2}=\left(\prod_{I_{A}^{*s}}g^{\alpha_{k}}\right)\theta^{s_{uid}^{\prime \prime }N_{A}^{*s}}\left(\prod_{i\in \left[\ell_{s}^{*}\right]}{\left(A_{\rho_{s}^{*}\left(i\right)}V_{i}\right)}^{a_{i}s_{uid}^{\prime \prime }+b_{i}}\right){\left(k_{0}\prod_{i=1}^{l}k_{i}{}^{c_{i}}\right)}^{w}{\left(\gamma_{1}\gamma_{2}{}^{\pi }\right)}^{w\beta }=\left(\prod_{I_{A}^{*s}}g^{\alpha_{k}^{\prime }+a^{q+1}}\right)\theta^{s_{uid}^{\prime \prime }N_{A}^{*s}}C_{1}^{L_{2}\left(\vec{c}\right)+\beta \left(\sigma_{1}+\pi \sigma_{2}\right)}\left(\prod_{i=1}^{\ell_{s}^{*}}{\left(g^{\varphi_{\rho_{s}^{*}\left(i\right)}}\prod_{j\in \left[n_{s}^{*}\right]}g^{-a^{j}M_{s}^{*\left(i,j\right)}N_{A}^{*s}}\right)}^{a_{i}s_{uid}^{\prime \prime }+b_{i}}\right)=\left(\prod_{I_{A}^{*s}}g^{\alpha_{k}^{\prime }}\right)g^{N_{A}^{*s}a^{q+1}}\theta^{s_{uid}^{\prime \prime }N_{A}^{*s}}C_{1}^{L_{2}\left(\vec{c}\right)+\beta \left(\sigma_{1}+\pi \sigma_{2}\right)}\prod_{i=1}^{\ell_{s}^{*}}S_{1,i}{}^{\varphi_{\rho_{s}^{*}\left(i\right)}}g^{N_{A}^{*s}\left(\sum_{i\in \left[\ell_{s}^{*}\right]}\sum_{j\in \left[n_{s}^{*}\right]}-a^{j}M_{s}^{*\left(i,j\right)}\left(a_{i}s_{uid}^{\prime \prime }+b_{i}\right)\right)}=\left(\prod_{I_{A}^{*s}}g^{\alpha_{k}^{\prime }}\right)g^{N_{A}^{*s}a^{q+1}}\theta^{s_{uid}^{\prime \prime }N_{A}^{*s}}C_{1}^{L_{2}\left(\vec{c}\right)+\beta \left(\sigma_{1}+\pi \sigma_{2}\right)}\prod_{i=1}^{\ell_{s}^{*}}S_{1,i}{}^{\varphi_{\rho_{s}^{*}\left(i\right)}}\theta^{-s_{uid}^{\prime \prime }N_{A}^{*s}}=\left(\prod_{I_{A}^{*s}}g^{\alpha_{k}^{\prime }}\right)g^{N_{A}^{*s}a^{q+1}}C_{1}^{L_{2}\left(\vec{c}\right)+\beta \left(\sigma_{1}+\pi \sigma_{2}\right)}\prod_{i=1}^{\ell_{s}^{*}}S_{1,i}{}^{\varphi_{\rho_{s}^{*}\left(i\right)}}$, where $\vec{a}\cdot M_{s}^{*}=\vec{1},\vec{b}\cdot M_{s}^{*}=\vec{0}$ and $\sum_{i\in \left[\ell_{s}^{*}\right]}\sum_{j\in \left[n_{s}^{*}\right]}-a^{j}M_{s}^{*\left(i,j\right)}\left(a_{i}s_{uid}^{\prime \prime }+b_{i}\right)=-as_{uid}^{\prime \prime }$.

Thus, ℬ can calculate $g^{a^{q+1}}={\left(\frac{S_{2}}{\left(\prod_{I_{A}^{*s}}g^{\alpha_{k}^{\prime }}\right)C_{1}^{L_{2}\left(\vec{c}\right)+\beta \left(\sigma_{1}+\pi \sigma_{2}\right)}\prod_{i=1}^{\ell_{s}^{*}}S_{1,i}{}^{\varphi_{\rho_{s}^{*}\left(i\right)}}}\right)}^{1/N_{A}^{*s}}$ and then break the q-PBDHE assumption by computing $e\left(g^{a^{q+1}},g^{w}\right)$. Let $E_{1}$ be the event that $L\left(\vec{c}\right)=0$ in some Signcryption query and $E_{2}$ be the event that $\varpi m\neq \varrho_{0}+\sum_{i=1}^{l}b_{i}\varrho_{i}$ in the forgery phase. Then we have $Pr\left[\neg E_{1}\wedge \neg E_{2}\right]=\frac{1}{\left(l+1\right)\varpi }\left(1-\frac{2q_{SC}}{\varpi }\right)$. If $\varpi =4q_{SC}$, then $Pr\left[\neg E_{1}\wedge \neg E_{2}\right]=\frac{1}{8\left(l+1\right)q_{SC}}$. Thus the advantage of ℬ solving the q-PBDHE assumption is at least $Adv_{ℬ}\geq \frac{\epsilon }{8\left(l+1\right)q_{SC}}$. The runtime of ℬ is at most $T^{\prime }=T+O\left(\ell_{s,m}n_{s,m}u_{m}+\left(n_{s,m}+\left|\tilde{U}\right|\ell_{s,m}n_{s,m}^{2}\right)q_{sk}+\left(\left|\tilde{U}\right|+\ell_{s,m}\right)q_{psk}+\left(l+\ell_{e,m}+\ell_{s,m}+\ell_{e,m}n_{e,m}\right)q_{SC}+\ell_{e,m}q_{DS}\right)T^{e}+O\left(\ell_{e,m}q_{DS}\right)T^{p}$. □

### 6.3. Signcryptor Privacy

Based on the security model defined in Definition 10, we prove that our scheme guarantees signcryptor privacy in Theorem 3.

**Theorem** **3.** *Our scheme guarantees the signcryptor privacy.*

**Proof.**  The challenger sends $PP,PK,{\left\{PK_{k},SK_{k}\right\}}_{I_{A}}$ to the adversary A. Then A outputs two signing attribute sets $\tilde{U_{0}^{s}},\tilde{U_{1}^{s}}$ satisfying ${ℛ}_{s}\left(\tilde{U_{0}^{s}}\right)=1={ℛ}_{s}\left(\tilde{U_{1}^{s}}\right)$. The challenger selects $𝒷\overset{R}{\leftarrow }\left\{0,1\right\}$ and computes $CT_{𝒷}$ with the secret signing key $SK_{uid,k}^{s,𝒷}\leftarrow SecretKeyGen\left(PP,PK_{k},SK_{k},PK_{uid},\tilde{U_{𝒷}^{s}}\right)$. Note that both the challenger and A can compute $SK_{uid,k}^{s,𝒷}$ for $\tilde{U_{𝒷}^{s}}$, where $k\in I_{A}$. Specifically, $K_{uid,k}^{s,𝒷}=g^{\alpha_{k}}\theta^{s_{uid}^{𝒷}},F_{uid,x}^{s,𝒷}=A_{x}^{s_{uid}^{𝒷}}$, where $s_{uid}^{𝒷}\overset{R}{\leftarrow }{\mathbb{Z}}_{p}^{*}$.

If the challenger uses $SK_{uid,k}^{s,0}$, then it can generate the ciphertext $CT_{0}=\left\{C_{0},C_{1}^{0},{\left\{C_{2,i}^{\prime 0},C_{2,i}^{\prime \prime 0},D_{i}^{\prime 0},D_{i}^{\prime \prime 0}\right\}}_{i\in \left[\ell_{e}\right]},{\left\{S_{1,i}^{0}\right\}}_{i\in \left[\ell_{s}\right]},S_{2}^{0},tt\right\}$ as follows.

$C_{1}^{0}=g^{w_{0}}$, $C_{3}^{0}={\left(\gamma_{1}\gamma_{2}{}^{\pi_{0}}\right)}^{w_{0}}$ where $\pi_{0}=H_{1}\left(C_{1}^{0}\right)$.

${\left\{C_{2,i}^{\prime 0}=\theta^{\lambda_{i}^{\prime 0}}A_{\rho_{e}\left(i\right)}^{-r_{i}^{\prime 0}},D_{i}^{\prime 0}=g^{r_{i}^{\prime 0}},C_{2,i}^{\prime \prime 0}=\lambda_{i}^{0}-\lambda_{i}^{\prime 0},D_{i}^{\prime \prime 0}=r_{i}-r_{i}^{\prime 0}\right\}}_{i\in \left[\ell_{e}\right]}$, ${\left\{S_{1,i}^{0}=g^{a_{i}^{0}s_{uid}^{\prime \prime 0}+b_{i}^{0}}\right\}}_{i\in \left[\ell_{s}\right]}$.

$H_{2}\left(\prod_{i\in \left[\ell_{s}\right]}S_{1,i}^{0},tt,{ℛ}_{s},{ℛ}_{e}\right)=\left(c_{1},c_{2},\ldots ,c_{l}\right)\in {\left\{0,1\right\}}^{l}$. $H_{3}\left(C_{0},C_{1}^{0},C_{3}^{0},{ℛ}_{s},{ℛ}_{e}\right)=\beta$.

$S_{2}^{0}=\left(\prod_{I_{A}^{s}}g^{\alpha_{k}}\right)\theta^{s_{uid}^{\prime \prime 0}N_{A}^{s}}\left(\prod_{i\in \left[\ell_{s}\right]}{\left(A_{\rho_{s}\left(i\right)}V_{i}\right)}^{v_{i}^{0}s_{uid}^{\prime \prime 0}+t_{i}^{0}}\right){\left(k_{0}\prod_{i=1}^{l}k_{i}{}^{c_{i}}\right)}^{w_{0}}{\left(C_{3}^{0}\right)}^{\beta }$, where $s_{uid}^{\prime \prime 0}=s_{uid}^{0}+s_{uid}^{\prime 0}$ and $s_{uid}^{\prime 0}\overset{R}{\leftarrow }{\mathbb{Z}}_{p}^{*}$.

If the challenger uses $SK_{uid,k}^{s,1}$, and sets $w_{0}=w_{1},\lambda_{i}^{\prime 0}=\lambda_{i}^{\prime 1},r_{i}^{0}=r_{i}^{1},r_{i}^{\prime 0}=r_{i}^{\prime 1},s_{uid}^{\prime 1}=s_{uid}^{\prime \prime 0}-s_{uid}^{1}$, then $\lambda_{i}^{0}=\lambda_{i}^{1}$, $s_{uid}^{\prime \prime 0}=s_{uid}^{\prime \prime 1}=s_{uid}^{\prime \prime }$. Thus $C_{1}^{0}=C_{1}^{1},\pi_{0}=\pi_{1},C_{2,i}^{\prime 0}=C_{2,i}^{\prime 1},C_{2,i}^{\prime \prime 0}=C_{2,i}^{\prime \prime 1},C_{3}^{0}=C_{3}^{1},D_{i}^{\prime 0}=D_{i}^{\prime 1},D_{i}^{\prime \prime 0}=D_{i}^{\prime \prime 1}$. The challenger sets $\vec{a^{1}}\cdot M_{s}=\vec{1}$ and sets $b_{i}^{1}=\left(a_{i}^{0}-a_{i}^{1}\right)s_{uid}^{\prime \prime }+b_{i}^{0}$. Then $\vec{b^{1}}\cdot M_{s}=\vec{0}$ and $a_{i}^{0}s_{uid}^{\prime \prime }+b_{i}^{0}=a_{i}^{1}s_{uid}^{\prime \prime }+b_{i}^{1}$. Hence $S_{1,i}^{0}=S_{1,i}^{1},S_{2}^{0}=S_{2}^{1}$, and $CT_{0}=CT_{1}$.

Similarly, if the challenger firstly uses $SK_{uid,k}^{s,1}$ to generate $CT_{1}=\left\{C_{0},C_{1}^{1},{\left\{C_{2,i}^{\prime 1},C_{2,i}^{\prime \prime 1},D_{i}^{\prime 1},D_{i}^{\prime \prime 1}\right\}}_{i\in \left[\ell_{e}\right]},{\left\{S_{1,i}^{1}\right\}}_{i\in \left[\ell_{s}\right]},S_{2}^{1},tt\right\}$, then it can generate $CT_{0}$ with $SK_{uid,k}^{s,0}$ and $CT_{1}=CT_{0}$. Therefore, A can only outputs a random guess ${𝒷}^{\prime }$ and the probability is at most $\frac{1}{2}$. □

### 6.4. Collusion Resistance

High-Level Overview

In our scheme, the secret keys of each user are associated the random elements $d_{uid},s_{uid}$ picked by CA which are difficult for each user, fog node, authority and cloud server to compute or learn. Therefore, the colluders such as the user, fog node, and cloud server cannot selectively replace or convert the components of the secret keys under the discrete logarithm assumption. Additionally, since uid chosen by CA is globally unique in the system and $d_{uid}$ and $s_{uid}$ are kept secret, secret keys generated from different authorities for the same uid can be tied together for signcryption and designcryption, and the secret keys generated for different users cannot be combined.

Let $S_{c}$ denote the set of colluders, and $\tilde{U^{d}}$ is the combined decryption attribute set of $S_{c}$. Recall that the message ℳ is blinded by $\prod_{k\in I_{A}^{e}}\Delta_{k}{}^{w}=\prod_{k\in I_{A}^{e}}e{\left(g,g\right)}^{\alpha_{k}w}$. It is infeasible to directly reconstruct $\prod_{k\in I_{A}^{e}}e{\left(g,g\right)}^{\alpha_{k}w}$ due to the blindness of $\alpha_{k}$ and the hardness of discrete logarithm assumption. Thus the colluders have to compute $\prod_{k\in I_{A}^{e}}e\left(K_{uid,k}^{d},C_{1}\right)$ and have to cancel the redundant element $e{\left(\theta ,g\right)}^{wN_{A}^{e}d_{uid}}=\prod_{k\in I_{A}^{e}}e{\left(g,g\right)}^{whd_{uid}}$, where $\theta =g^{h}$. Due to BDH assumption, the only way to cancel $e{\left(\theta ,g\right)}^{wN_{A}^{e}d_{uid}}$ is to compute the denominator $\prod_{k\in I_{A}^{e}}\prod_{i\in I_{A_{k}}}{\left[e\left(C_{2,i}^{\prime }\theta^{C_{2,i}^{\prime \prime }}A_{\rho_{e}\left(i\right)}^{-D_{i}^{\prime \prime }},g^{d_{uid}}\right)e\left(D_{i}^{\prime }g^{D_{i}^{\prime \prime }},F_{uid,\rho_{e}\left(i\right)}^{d}\right)\right]}^{\sigma_{i}N_{A}^{e}}$ in PartialDecryption algorithm, which means $F_{uid,\rho_{e}\left(i\right)}^{d}=A_{\rho_{e}\left(i\right)}^{d_{uid}}$ with the same $d_{uid}$ holds for all $\rho_{e}\left(i\right)\in \tilde{U^{d}}$. However, since the colluders are individually unauthorized for decryption, none of the colluders holds $A_{\rho_{e}\left(i\right)}^{d_{uid}}$ for all $\rho_{e}\left(i\right)\in \tilde{U^{d}}$ simultaneously. Moreover, since the secret key cannot be replaced, converted or combined, ${\left\{A_{\rho_{e}\left(i\right)}^{d_{uid}}\right\}}_{U_{uid}\in S_{c},\rho_{e}\left(i\right)\in \tilde{U^{d}}}$ are associated with different $d_{uid}$. Hence the colluders cannot successfully decrypt the ciphertext even though $\tilde{U^{d}}$ satisfies the encrypt predicate defined in the ciphertext. Specifically, according to Theorems 1 and 2, we can prove that our scheme guarantees the collusion resistance under q-PBDHE assumption in Theorem 4.

**Theorem** **4.** *The proposed data access control scheme is collusion resistance.*

**Proof.**  For the designcryptor, we state that the security game defined in Definition 9 implies the collusion resistance. Suppose that $S_{c}$ denotes the set of colluders who are unauthorized for decryption and $\tilde{U^{d}}=\cup {\left\{\tilde{U_{i}^{d}}\right\}}_{i\in S_{c}}$. If the colluders can decrypt $CT^{*}$ when ${ℛ}_{e}^{*}\left(\tilde{U^{d}}\right)=1$, then the algorithm ℬ which can solve the q-PBDHE assumption can be constructed as follows.

In the initialization phase, the challenger sets ${ℛ}_{e}^{*}$ as the selected challenge encryption predicate. In $O^{sk}$, A queries for the secret decryption key corresponding to the colluder’s individual attribute set $\tilde{U_{i}^{d}}$. Since the colluders are individually unauthorized for decryption, we have ${ℛ}_{e}^{*}\left(\tilde{U_{i}^{d}}\right)=0$, which satisfies the constraint of $O^{sk}$ defined in Definition 8. Then in challenge phase, the challenger encrypts ${ℳ}_{\hat{𝒷}}$ under ${ℛ}_{e}^{*}$. If the colluders can decrypt the ciphertext, then A can guess the bit $\hat{𝒷}$, and thus ℬ can solve the q-PBDHE assumption with non-negligible probability.

Similarly, for the signcryptor, the Theorem 2 guarantees that no colluders such as users, fog nodes or cloud server can generate the signature by combining their information if they are individually unauthorized to sign the plaintext. Otherwise, the colluders can build an adversary and output a forgery to win the game in Definition 9 and break q-PBDHE assumption.

Therefore, the colluding users, fog nodes, and cloud server cannot sign or decrypt the data, and our OMDAC-ABSC scheme guarantees collusion resistance. □

### 6.5. Revocation Security

Assume the attribute x of U is revoked from $AA_{k}$. $AA_{k}$ issues the update secret keys $dUK_{x}=g^{d_{uid}\left(\varphi_{x}^{\prime }-\varphi_{x}\right)},sUK_{x}=g^{s_{uid}\left(\varphi_{x}^{\prime }-\varphi_{x}\right)}$ and sends the keys to the non-revoked users. $dUK_{x}$ and $sUK_{x}$ are associated with the secret value $d_{uid},s_{uid}$ chosen by CA and attribute version key $\varphi_{x}^{\prime },\varphi_{x}$ chosen by $AA_{k}$. Therefore, due to the blindness of $d_{uid},s_{uid},\varphi_{x}^{\prime }$, and $\varphi_{x}$, the revoked user U cannot update his/her secret signing or decryption key, even though he/she can corrupt some attribute authorities (not the authority $AA_{k}$ corresponding to x) or collude with the non-revoked user.

**Theorem** **5.** *Our OMDAC-ABSC scheme guarantees the forward and backward revocation security.*

**Proof.**   

Forward Security. If there exists i such that $\rho_{s}\left(i\right)=x$, the newly joined user can sign the plaintext and generate the signature component $S_{2}$ associated with $A_{x}^{\prime }$, which is the same as the updated attribute public key of $AA_{k}$. Thus the Verify algorithm holds if user’s signing attributes satisfy the signing predicate. Otherwise, the newly joined user’s secret decryption keys are all associated with $A_{x}^{\prime }$, which is the same as that in the components $C_{2,i}^{\prime }$. Thus the newly joined user can decrypt ciphertext if his/her attribute set satisfies the embedded encryption predicate.

Backward security. If there exists i such that $\rho_{s}\left(i\right)=x$, and the revoked user reverse the signature component $S_{2}$ back to the non-revoked state which is associated with $A_{x}$, then the Verify algorithm cannot hold since the attribute public key of $AA_{k}$ has been updated to $A_{x}^{\prime }$.

Otherwise, assume $CT_{old}^{\prime }$ denotes the ciphertext which is updated from $CT_{old}$ in attribute revocation phase, we have $C_{2,i}^{\prime }=\theta^{\lambda_{i}^{\prime }}A_{\rho_{e}\left(i\right)}^{\prime }{}^{-\left(r_{i}^{\prime }+r_{i}^{\prime \prime }\right)}$ and $D_{i}^{\prime }=g^{r_{i}^{\prime }+r_{i}^{\prime \prime }}$. It is hard for the revoked user to cancel $cUK_{i}$ and $g^{r_{i}^{\prime \prime }}$ since they are associated with the values $\varphi_{x}^{\prime },\varphi_{x}$ which are secretly chosen by $AA_{k}$ and $r_{i}^{\prime \prime }$ randomly picked by cloud server. Therefore, the revoked user cannot reverse the $CT_{old}^{\prime }$ back to $CT_{old}$.

For the ciphertext $CT_{new}$ which is uploaded after the attribute revocation phase, we have $C_{2,i}^{\prime }=\theta^{\lambda_{i}^{\prime }}A_{\rho_{e}\left(i\right)}^{\prime }{}^{-r_{i}^{\prime }}$ for i such that $\rho_{e}\left(i\right)=x$. The revoked user cannot transform these components into the ones associated with $A_{\rho_{e}\left(i\right)}$ due to the blindness of the attribute version keys $\varphi_{x}^{\prime },\varphi_{x}$ chosen by $AA_{k}$ and random element $r_{i}^{\prime }$ picked by fog node. Therefore, our OMDAC-ABSC scheme guarantees the forward and backward revocation security. □

## 7. Scheme Analysis

### 7.1. Security and Functionality

In this subsection, we detail the comprehensive security and functionality comparison among the proposed scheme and some MA-ABE schemes [21,22,23,24,25,26], CP-ABSC schemes [12,13,14,15] and ABE based schemes used for fog computing [16,17,18,19,20] in Table 1, Table 2 and Table 3. Therein, ✓ represents the capability to achieve the corresponding index, whereas ⨯ denotes the opposite. MBF represents monotone Boolean function, and TG represents the threshold gate.

Table 1, Table 2 and Table 3 show that our scheme supports many useful properties, such as multi-authority, collusion resistance, computation outsourcing, anonymous authentication, expressiveness, public verifiability and attribute revocation. Our scheme also realizes the security in the standard model.

### 7.2. Asymptotic Complexity and Performance

This section numerically analyzes the asymptotic complexity and performance of the proposed OMDAC-ABSC scheme against some MACP-ABE schemes [21,22,24,25,26], CP-ABSC schemes [12,13,14,15], and ABE based schemes [16,17,18,19,20] used for fog computing in terms of the size of secret key, ciphertext and update key, and computation overhead (exponentiations and pairing computations) of Signcryption, DeSigncryption and UpCiphertext algorithms. We focus on the computation overhead on the user side because of the limited computation resources. For simplicity, in asymptotic complexity analysis we ignore the cost time of Hash functions and operations in ${\mathbb{Z}}_{p}$. Table 4 summarizes the notations used in this section.

#### 7.2.1. Asymptotic Complexity

Table 5 details the storage comparison on MACP-ABE schemes. It is clear that the size of the secret decryption key in our OMDAC-ABSC is larger than that in [24,25] due to the components ${\left\{K_{uid,k}^{d}\right\}}_{k\in I_{A}}$. Table 5 also illustrates that the size of ciphertext in our scheme is larger than that in [21,22,26], and has the advantage over [25]. Since our scheme supports public verification of signcryptor’s attributes, the ciphertext contains the signature components ${\left\{S_{1,i}\right\}}_{i\in \left[\ell_{s}\right]},S_{2}$, which result in a reducing $\left(1+l_{s}\right)\left|G\right|$ of storage overhead. Although the scheme in [24] can also verify the data owner’s attributes, it requires $2+2l_{s}$ signature group elements and is not publicly verifiable since it needs the plaintext message in verification algorithm. Additionally, both of our scheme and [25] requires the data owner to compute the ciphertext components ${\left\{C_{2,i}^{\prime \prime },D_{i}^{\prime \prime }\right\}}_{i\in \left[\ell_{e}\right]}$ when performing User_Signcryption algorithm. This cost is $2l_{e}\left|{\mathbb{Z}}_{p}\right|$.

For attribute revocation, it is apparent that our scheme and [22] incur relatively the same storage overhead. Compared with [26], our scheme requires the attribute authority supervising the revoked attribute x to compute the ciphertext update key $cUK={\left\{{\left(D_{i}^{\prime }\right)}^{\varphi_{x}-\varphi_{x}^{\prime }}\right\}}_{\rho_{e}\left(i\right)=x}$ when x is selected as an encryption attribute, and thus incurs at most $l_{e}$ group elements, whereas the scheme [26] only sends $\varphi_{x}-\varphi_{x}^{\prime }$ to the cloud. However, as shown in [22], DAC-MACS [26] cannot guarantee backward revocation security.

Table 6 shows the computation overhead comparison of Signcryption and Decryption algorithms on the user side and UpCiphertext algorithm on the cloud. From the table, we can see that the encryption and decryption cost of our scheme are both irrelevant to the number of attributes. In data signcryption phase, our scheme asks fog nodes to compute and generate part of the ciphertext which is associated with the signing and encryption predicates. Thus the signcryption cost of data owner can be reduced as $T_{G_{T}}^{e}+3T_{G}^{e}$ in encryption and $\left(l_{s}+l+2\right)T_{G}^{e}$ in signing. In decryption phase, our scheme only incurs the cost of one exponentiation in $G_{T}$. Hence the performance of ours is better than most schemes except for [25]. To guarantee the CCA security in the standard model (see Theorem 1), our scheme requires the data owner to compute the components $C_{1}$ and $C_{3}$, which results in a slight reducing $3T_{G}^{e}$ of computation efficiency compared with [25]. However, our scheme performs better than [25] with respect to attribute revocation. Moreover, the DAC-MACS scheme in [26] only incurs the cost of $l_{e}$ exponentiations in G in ciphertext update phase, while our scheme incurs twice this cost. The reason is that we re-randomize $C_{2,i}^{\prime }$ and $D_{i}^{\prime }$ in UpCiphertext algorithm to realize the backward revocation security.

If we set $N_{A}=1$, then the proposed scheme is a traditional CP-ABSC scheme. In Table 7, we compare the asymptotic complexity of OMDAC-ABSC with CP-ABSC schemes [12,13,14,15]. As seen from Table 7, the size of the secret key is linear to the size of the attribute universe, which is not different between our scheme and others. Our scheme incurs a slight reducing $l_{e}\left|G\right|+2l_{e}\left|{\mathbb{Z}}_{p}\right|$ of storage overhead than other schemes on the ciphertext. The reason is that we add ${\left\{C_{2,i}^{\prime \prime },D_{i}^{\prime },D_{i}^{\prime \prime }\right\}}_{i\in \left[\ell_{e}\right]}$ to realize the attribute revocation and outsourced encryption, which are not considered in other schemes. Meanwhile, the ciphertext in our scheme consists of $l_{s}+1$ group elements for verification, while that in [12] is $2l_{s}+2$. Table 7 also indicates that our scheme incurs less computation overhead of DeSigncryption on the user side than do the other schemes since most costly job of decryption is outsourced to fog nodes. Compared with [14], our construction requires $3+l_{s}$ pairing operations in total in decryption (user side) and verification, whereas in [14], $\left(5+l_{s}\right)$ pairings are needed. Moreover, since our scheme supports public verifiability, the verification algorithm can be performed by a trusted intermediate party. Thus the user can recover the plaintext within one exponentiation in $G_{T}$. In contrast, the schemes in [12,13,15] are not publicly verifiable, and thus incur large amount of computation overhead in verification and decryption on the user side. In [12,13], the number of pairings is linear to the number of attributes. In [15], although the size of ciphertext is only 6|G|, eight pairings are required to recover the plaintext.

Table 8 details the storage and computation overhead comparison of our scheme and some ABE based data access control schemes for fog computing. Since the schemes in [16,18,19,20] do not support multi-authority, we set $N_{A}=1$ in our scheme for comparison. It is illustrated that the size of secret decryption key in OMDAC-ABSC is less than that in others. Since our scheme enables any trusted third party to verify the data owner’s attributes, the ciphertext contains the signature components ${\left\{S_{1,i}\right\}}_{i\in \left[\ell_{s}\right]},S_{2}$, which result in a reducing $\left(1+l_{s}\right)\left|G\right|$ of storage overhead on the cloud side. For encryption, on the user side, our scheme incurs $3T_{G}^{e}$ to compute $C_{1}$ and $C_{3}$ and thus is less efficient than [17]. However, our scheme guarantees the CCA security, which is not considered in [17]. For decryption, on the user side, our scheme and [17] both incurs less computation overhead than other schemes since the two schemes only require one exponentiation in $G_{T}$. Therefore, our scheme is efficient from a computation point of view.

#### 7.2.2. Performance

We implement the whole architectures of MACP-ABE schemes [21,22,24,25,26], CP-ABSC schemes [12,13,14,15] and our scheme with Pairing-based Cryptography (PBC) library version 0.5.14 on an Ubuntu system 14.04 with a 2.6 GHz processor and 4G RAM. We employ 160-bit Type A elliptic curve group constructed on $y^{2}=x^{3}+x$ over a 512-bit finite field. The computation cost for one pairing operation is 2.9 ms, and that of exponentiation on G and $G_{T}$ are 0.7 and 0.2 ms, respectively. Each value in Figure 3, Figure 4, Figure 5, Figure 6, Figure 7 and Figure 8 is the mean of 10 simulation trials.

**Figure 3:**
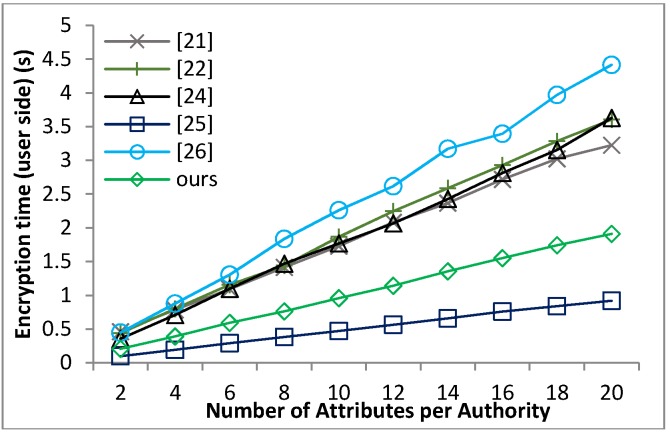
Encryption (user side).

**Figure 4:**
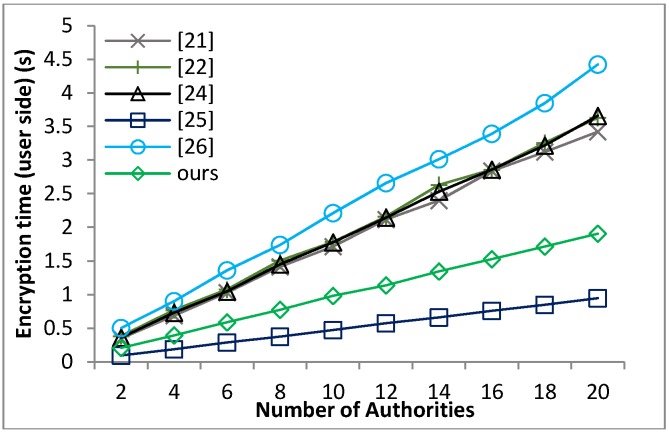
Encryption (user side).

**Figure 5:**
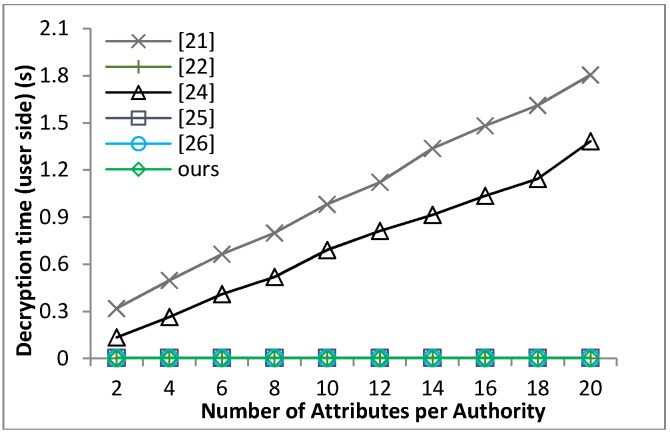
Decryption (user side).

**Figure 6:**
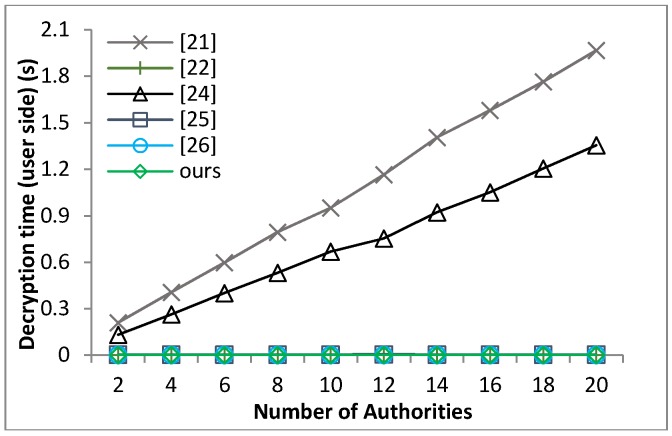
Decryption (user side).

For simplicity, suppose each user holds the same number of attributes $N_{AA}$ from each authority and $\left|\tilde{AA_{k}}\right|=\left|\tilde{AA_{k}}\cap \tilde{U^{d}}\right|=N_{AA}$, where $k\in \left[N_{A}\right]$. $N_{A}^{e}=N_{A}^{s}=N_{A}$. Then, in signcryption we set $l_{e}=l_{s}=N_{AA}\times N_{A}$, and thus the comparison of computation overhead of Signcryption (without signing) and Decryption algorithms on the user side between our scheme and [21,22,24,25,26] can be conducted according to parameters $N_{A}$ and $N_{AA}$. We also generate the signing and encryption predicates as AND-gate in the form of $\left(a_{1}\;\mathrm{and}\;a_{2}\;\mathrm{and}\ldots \mathrm{and}\;a_{l_{s}}\right)$ and $\left(a_{1}\;\mathrm{and}\;a_{2}\;\mathrm{and}\ldots \mathrm{and}\;a_{l_{e}}\right)$. In Figure 3 and Figure 5, we set $N_{A}=10$, while in Figure 4 and Figure 6, we assume $N_{AA}=10$. During the comparison between our scheme and the ones in [21,22,24,25,26], we do not take into account the signing protocol since the schemes in [21,22,25,26] do not support attribute-based signature.

Figure 3 and Figure 4 show that the encryption algorithm in our scheme is more efficient than that in [21,22,24,26]. The reason is that the most costly job of encryption has been outsourced to the fog nodes. Although our scheme incurs more computation overhead than the one in [25], we realize CCA security in the standard model and attribute-level revocation. Figure 5 and Figure 6 give the comparison of decryption time on the user side. It is illustrated that the performance of our scheme is relatively the same as that of [22,25,26], and is better than that of [21,24] because our scheme only incurs one exponentiation and one multiplication in $G_{T}$.

**Figure 7:**
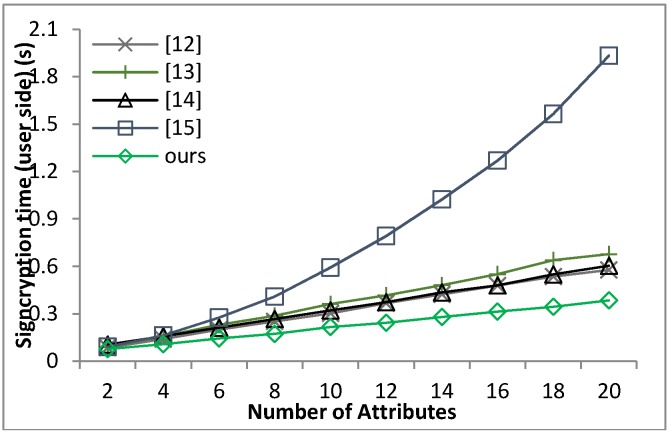
Signcryption (user side).

**Figure 8:**
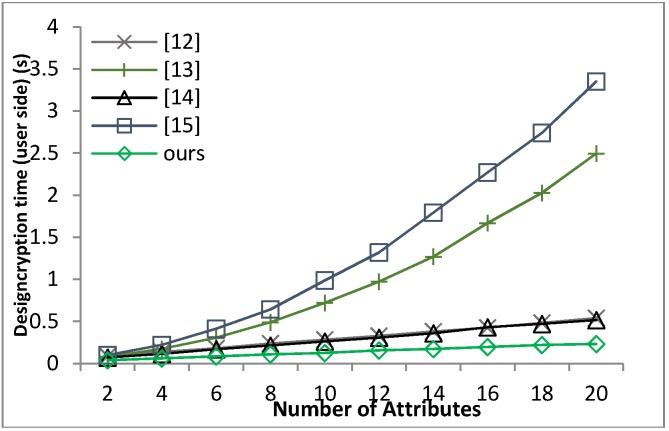
Designcryption (user side).

Assume that $N_{A}=1$ and $\ell_{e}=\ell_{s}=N_{AA}$. Figure 7 and Figure 8 describe the comparison of computation overhead of Signcryption and DeSigncryption algorithms among the schemes [12,13,14,15] and ours. It is clear that our Signcryption algorithm incurs less computation overhead than other schemes because of the outsourced signcryption. Since our scheme and Y. Sreenivasa’s scheme [14] are publicly verifiable, the Verify(PP,CT) algorithm can be outsourced to a trusted party, and then our scheme needs only one exponentiation and one multiplication in $G_{T}$ on the user side to recover the plaintext message.

**Figure 9:**
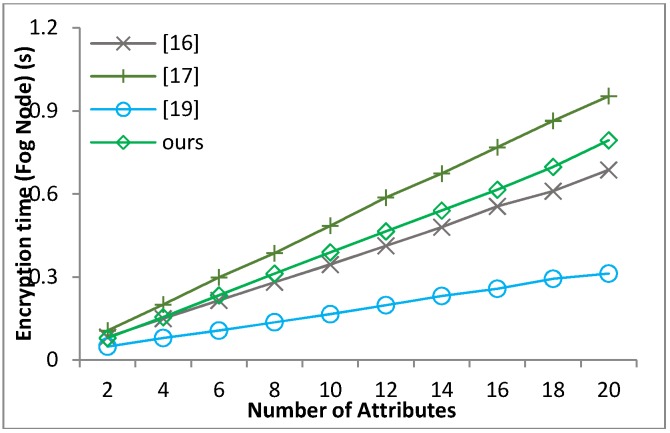
Encryption (fog node side).

**Figure 10:**
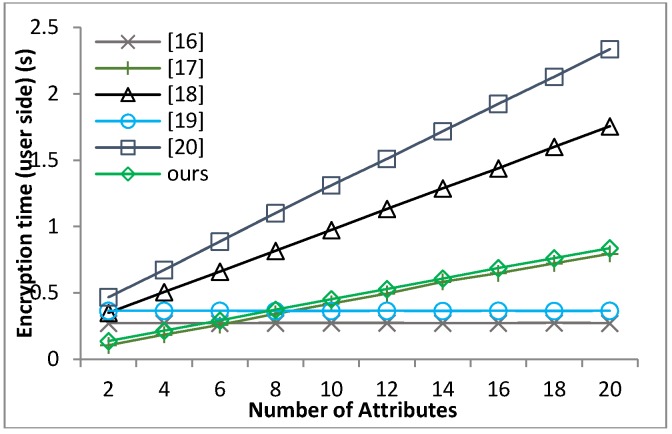
Encryption (user side).

**Figure 11:**
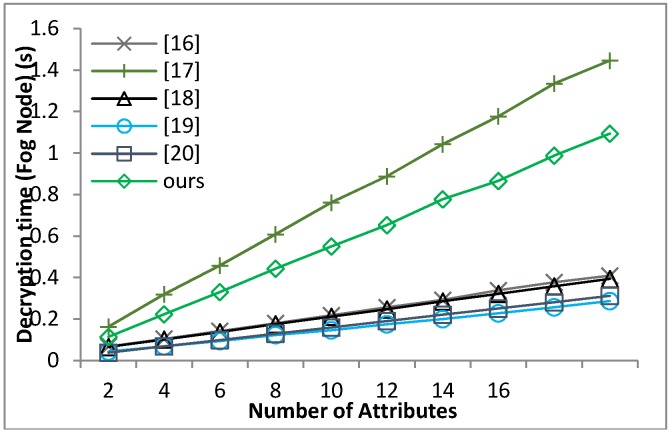
Decryption (fog node side).

**Figure 12:**
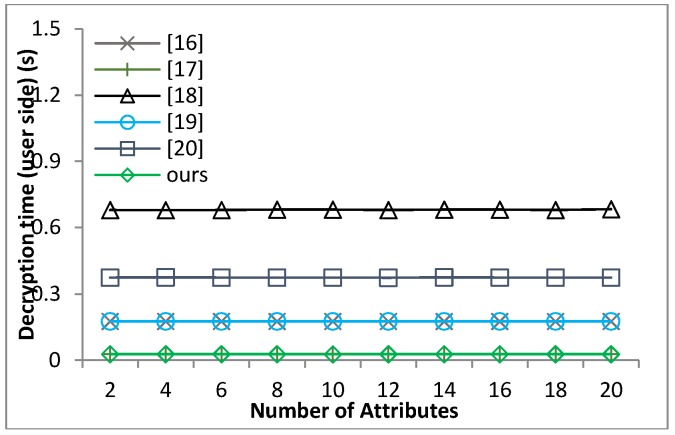
Decryption (user side).

Moreover, we simulate the schemes in [16,17,18,19,20] and our scheme on an android phone (MEIZU m1 note platform with an ARM Cortex A53-based processor MT6752@1.7 GHz, Android 5.1, and 2GB RAM) as user’s IoT device and a laptop (2.6 GHz processor, Ubuntu system 14.04, and 4G RAM) as the fog node. The underlying curve for pairings is also Type A curve in JPBC 2.0.0 [18], where the running time for pairing is 6 ms in Ubuntu system and 175 ms in Android. For comparison, we set $N_{A}=1$ in our scheme and do not consider the signing protocol since the schemes in [16,18,19,20] do not support multi-authority and the schemes in [16,17,18,19,20] do not support attribute-based signature. Figure 9 and Figure 10 show the comparison of computation overhead of encryption algorithm and Figure 11 and Figure 12 show the comparison of decryption algorithm. The results are the average number of 10 runs. In Figure 9 we only compare the cost time of encryption on fog node between ours and the schemes in [16,17,19] since the schemes in [18,20] do not support encryption outsourcing.

It is illustrated in Figure 10 that the computation time of encryption algorithm on data owner in our scheme is basically the same as that in [17], and is smaller than that in [18,20] because of the encryption outsourcing. Compared with [16,19], the encryption algorithm in our scheme incurs slightly more computation overhead since our scheme requires the data owner to sample ${\left\{C_{2,i}^{\prime \prime },D_{i}^{\prime \prime }\right\}}_{i\in \left[\ell_{e}\right]}$ and perform one Hash function $\pi =H_{1}\left(C_{1}\right)$ (we do not take into account the Hash functions $H_{2}$ and $H_{3}$ here since they are involved in signing protocol). However, the encryption time is approximately 0.14–0.8 s, which is acceptable to the end users.

Figure 11 indicates that on the fog node side, the decryption algorithm of our scheme incurs more computation overhead than the schemes in [16,18,19,20]. However, Figure 12 shows that our scheme performs better than other schemes except for [17] in efficiency of decryption time on the user side. This is because our scheme outsources the most computation-consuming job of decryption to the fog node and only incurs the cost of one exponentiation and one multiplication in $G_{T}$ on the user side. In Figure 11, the decryption time of our scheme one the fog node is approximately 0.1–1 s, which increases almost linearly with the number of attributes. 

However it is shown in Figure 12 that the running time of FullDecryption algorithm is nearly 0.03 s, which is acceptable for the end user. Since our scheme is public verifiable, the verification can be performed on any trusted third party and does not increase the computation burden of the user. Additionally, Huang et al. [16] and Zhang et al. [19] only support threshold access policy, while our scheme supports any monotone Boolean function. Overall, our scheme performs well in encryption and decryption on the user side and supports additional useful properties such as multi authorities, anonymous authentication, and public verifiability.

## 8. Conclusions

In this paper, we proposed OMDAC-ABSC scheme for data sharing in fog computing system. The proposed scheme realizes the security in the standard model and supports many practical properties, such as confidentiality, fine-grained access control, anonymous authentication, attribute revocation, and public verifiability. The heavy computation operations of the signcryption and designcryption algorithms are outsourced to the fog nodes making our scheme more efficient and more suitable for fog computing than the existing ABSC schemes. The security analysis, asymptotic complexity, and performance comparisons indicate that our construction hits a good balance between the security and overhead efficiency.

One problem with outsourced decryption is to verify that whether the partial decryption performed by fog nodes is correct. In ABE scheme, verifiable outsourcing has been adopted to overcome this problem, as in [17,30,31,32]. A similar technique can be used in our ABSC construction to address verifiable outsourcing, which will be our future work. Moreover, realizing a fully secure MACP-ABSC based access control scheme instead of a selectively secure scheme will be another challenge.

## Figures and Tables

**Table 1:** Security and Functionality Comparison of MACP-ABE Schemes.

Schemes	[21]	[22]	[23]	[24]	[25]	[26]	Ours
Collusion Resistance	⨯	✓	✓	✓	✓	✓	✓
Standard Model	✓	⨯	⨯	⨯	⨯	⨯	✓
Encryption Predicate	MBF	MBF	MBF	MBF	MBF	MBF	MBF
Encryption Outsourcing	⨯	⨯	⨯	⨯	✓	⨯	✓
Decryption Outsourcing	⨯	✓	⨯	⨯	✓	✓	✓
Anonymous Authentication	⨯	⨯	⨯	✓	⨯	⨯	✓
Attribute Revocation	⨯	✓	⨯	⨯	⨯	✓	✓

**Table 2:** Security and Functionality Comparison of CP-ABSC Schemes.

Schemes	[12]	[13]	[14]	[15]	Ours
Collusion Resistance	✓	✓	✓	✓	✓
Standard Model	✓	⨯	✓	⨯	✓
Signcryptor Privacy	✓	✓	✓	⨯	✓
Signing Predicate	MBF	MBF	MBF	MBF	MBF
Encryption Predicate	MBF	MBF	MBF	TG	MBF
Signcryption Outsourcing	⨯	⨯	⨯	⨯	✓
Designcryption Outsourcing	⨯	⨯	⨯	⨯	✓
Multi-Authority	⨯	⨯	⨯	⨯	✓
Public Verifiability	⨯	⨯	✓	✓	✓
Attribute Revocation	⨯	⨯	⨯	⨯	✓

**Table 3:** Security and Functionality Comparison of ABE based Schemes for Fog Computing.

Schemes	[16]	[17]	[18]	[19]	[20]	Ours
Collusion Resistance	✓	✓	✓	✓	✓	✓
Standard Model	⨯	⨯	⨯	✓	✓	✓
Encryption Predicate	TG	MBF	TG	TG	MBF	MBF
Encryption Outsourcing	✓	✓	⨯	✓	⨯	✓
Decryption Outsourcing	✓	✓	✓	✓	✓	✓
Multi-Authority	⨯	✓	⨯	⨯	⨯	✓
Anonymous Authentication	✓	⨯	⨯	⨯	⨯	✓
Attribute Revocation	⨯	⨯	⨯	✓	⨯	✓

**Table 4:** Notations.

Notations	Meaning
$T_{G}^{e}/T_{G_{T}}^{e}$	Running time required for one exponentiation in G and $G_{T}$.
$T^{p}$	Running time for one pairing operation.
$N_{A}$	Number of involved authorities.
$\left|G\right|/\left|G_{T}\right|/\left|{\mathbb{Z}}_{p}\right|$	Size of the element in G, $G_{T}$, and ${\mathbb{Z}}_{p}$.
$l_{e}\;/l_{s}$	Number of required attributes in decryption and verification.
$\left|\tilde{U^{d}}\right|$	Number of decryption attributes.
$\left|\tilde{U}\right|$	Number of signing and decryption attributes.
S	Least interior nodes satisfying the access policy tree.

**Table 5:** Storage Comparison of MACP-ABE based Schemes.

Schemes	Secret Decryption Key	Ciphertext	Update Key	
			Secret Key Update	Ciphertext Update Key
[21]	$\left(6N_{A}+\left|\tilde{U^{d}}\right|\right)\left|G\right|$	$\left|G_{T}\right|+\left(3N_{A}+2l_{e}\right)\left|G\right|$	-	-
[22]	$\left(2+2\left|\tilde{U^{d}}\right|\right)\left|G\right|$	$\left|G_{T}\right|+\left(3+N_{A}+3l_{e}\right)\left|G\right|$	2|G|	$l_{e}\left|G\right|$
[24]	$\left|\tilde{U^{d}}\right|\left|G\right|$	$\left(l_{e}+1\right)\left|G_{T}\right|+\left(2+2l_{e}+2l_{s}\right)\left|G\right|$	-	$l_{e}\left|G_{T}\right|$
[25]	$\left|\tilde{U^{d}}\right|\left|G\right|$	$\left(3l_{e}+1\right)\left|G_{T}\right|+4l_{e}\left|G\right|+2l_{e}\left|{\mathbb{Z}}_{p}\right|$	$\left(2\left|G\right|+\left|G_{T}\right|\right)l_{e}$	$l_{e}\left|G_{T}\right|$
[26]	$\left(2N_{A}+\left|\tilde{U^{d}}\right|\right)\left|G\right|$	$\left|G_{T}\right|+\left(1+3l_{e}\right)\left|G\right|$	|G|	$\left|{\mathbb{Z}}_{p}\right|$
Ours	$\left(N_{A}+\left|\tilde{U^{d}}\right|\right)\left|G\right|$	$\left|G_{T}\right|+\left(2+2l_{e}+l_{s}\right)\left|G\right|+2l_{e}\left|{\mathbb{Z}}_{p}\right|$	|G|	$l_{e}\left|G\right|$

**Table 6:** Time Comparison of *Signcryption*, *Decryption* and *UpCiphertext*.

Schemes	*Signcryption* (User Side)		*Decryption* (User Side)	*UpCiphertext*
	Encryption	Signing		
[21]	$N_{A}T_{G_{T}}^{e}+\left(3N_{A}+3l_{e}\right)T_{G}^{e}$	-	$\left(4N_{A}+2l_{e}\right)T^{p}+\left(N_{A}+l_{e}\right)T_{G_{T}}^{e}$	-
[22]	$2N_{A}T_{G_{T}}^{e}+\left(3+N_{A}+4l_{e}\right)T_{G}^{e}$	-	$T_{G_{T}}^{e}$	$2l_{e}T_{G}^{e}$
[24]	$\left(1+2l_{e}\right)T_{G_{T}}^{e}+3l_{e}T_{G}^{e}$	$\left(2+3l_{s}+2l_{s}n_{s}\right)T_{G}^{e}$	$2l_{e}T^{p}$	$\left(1+2l_{e}\right)T^{p}$
[25]	$T_{G_{T}}^{e}$	-	$2T_{G_{T}}^{e}$	$l_{e}T^{p}$
[26]	$N_{A}T_{G_{T}}^{e}+\left(1+5l_{e}\right)T_{G}^{e}$	-	$T_{G_{T}}^{e}$	$l_{e}T_{G}^{e}$
Ours	$T_{G_{T}}^{e}+3T_{G}^{e}$	$\left(l_{s}+l+2\right)T_{G}^{e}$	$T_{G_{T}}^{e}$	$2l_{e}T_{G}^{e}$

**Table 7:** Asymptotic Complexity Comparison of CP-ABSC based schemes.

Schemes	Secret Key	Ciphertext	*DeSigncryption*	
			Verification	Decryption (User Side)
[12]	$\left(4+\left|\tilde{U}\right|\right)\left|G\right|$	$\left(\begin{array}{c} 4+l_{e}\\ +2l_{s} \end{array}\right)\left|G\right|$	$\left(2+2l_{s}\right)T^{p}+\left(2l_{s}+3\right)T_{G}^{e}$	$\left(2+2l_{e}\right)T^{p}+l_{e}T_{G_{T}}^{e}$
[13]	$\left(4+\left|\tilde{U}\right|\right)\left|G\right|$	$\left|G_{T}\right|+\left(\begin{array}{c} 5+l_{e}\\ +l_{s}+\\ n_{s} \end{array}\right)\left|G\right|$	$\left(6+l_{s}n_{s}+2l_{s}\right)T^{p}+l_{s}T_{G_{T}}^{e}+2l_{s}n_{s}T_{G}^{e}$	$2l_{e}T^{p}+l_{e}T_{G_{T}}^{e}$
[14]	$\left(4+\left|\tilde{U}\right|\right)\left|G\right|$	$\left(\begin{array}{c} 5+l_{e}\\ +l_{s} \end{array}\right)\left|G\right|$	$\left(3+l_{s}\right)T^{p}+\left(l_{s}+l+1\right)T_{G}^{e}$	$2T^{p}+3l_{e}T_{G}^{e}$
[15]	$\left(6l_{e}+3l_{s}\right)\left|G\right|$	6|G|	$6T^{p}$	$2T^{p}+\left(4l_{e}+4l_{e}^{2}\right)T_{G}^{e}$
Ours	$\left(2+\left|\tilde{U}\right|\right)\left|G\right|$	$\left|G_{T}\right|+\left(2+2l_{e}+l_{s}\right)\left|G\right|+2l_{e}\left|{\mathbb{Z}}_{p}\right|$	$\left(3+l_{s}\right)T^{p}+\left(l_{s}+l+1\right)T_{G}^{e}$	$T_{G_{T}}^{e}$

**Table 8:** Storage and Computation Overhead Comparison of ABE based Schemes for Fog Computing.

Schemes	Secret Decryption Key	Ciphertext	Encryption		Decryption	
			Fog Node	User	Fog Node	User
[16]	$\left(2+2\left|\tilde{U^{d}}\right|\right)\left|G\right|$	$\left|G_{T}\right|+\left(3+2\left|\tilde{U^{d}}\right|\right)\left|G\right|$	$\left(2+2\left|\tilde{U^{d}}\right|\right)T_{G}^{e}$	$T_{G_{T}}^{e}+3T_{G}^{e}$	$\left(2\left|\tilde{U^{d}}\right|+4\right)T^{p}+\left(2S+2\right)T_{G_{T}}^{e}$	$T^{p}$
[17]	$\left(2+\left|\tilde{U^{d}}\right|\right)\left|G\right|$	$\left|G_{T}\right|+\left(1+2l_{e}\right)\left|G\right|+\left(2+2l_{e}\right)\left|{\mathbb{Z}}_{p}\right|$	$4l_{e}T_{G}^{e}+T_{G}^{e}$	$T_{G_{T}}^{e}$	$\left(1+2l_{e}\right)T^{p}+\left(1+7l_{e}\right)T_{G}^{e}$	$T_{G_{T}}^{e}$
[18]	$\left(1+2\left|\tilde{U^{d}}\right|\right)\left|G\right|$	$\left(2+\left|\tilde{U^{d}}\right|\right)\left|G\right|+2l\left|{\mathbb{Z}}_{p}\right|$	-	$T_{G_{T}}^{e}+\left(2+\left|\tilde{U^{d}}\right|\right)T_{G}^{e}$	$\left(3+3\left|\tilde{U^{d}}\right|\right)T^{p}$	$4T_{G_{T}}^{e}$
[19]	$\left(3+\left|\tilde{U^{d}}\right|\right)\left|G\right|$	$\left|G_{T}\right|+\left(3+\left|\tilde{U^{d}}\right|\right)\left|G\right|$	$\left(2+\left|\tilde{U^{d}}\right|\right)T_{G}^{e}$	$2T_{G_{T}}^{e}+4T_{G}^{e}$	$\left(\left|\tilde{U^{d}}\right|+2\right)T^{p}+\left(2S+2\right)T_{G_{T}}^{e}$	$T^{p}$
[20]	$\left(2+\left|\tilde{U^{d}}\right|\right)\left|G\right|$	$\left(2+2l_{e}\right)\left|G\right|+l\left|{\mathbb{Z}}_{p}\right|$	-	$T_{G_{T}}^{e}+\left(3+3l_{e}\right)T_{G}^{e}$	$\left(1+2l_{e}\right)T^{p}+l_{e}T_{G_{T}}^{e}$	$T_{G_{T}}^{e}+2T_{G}^{e}$
Ours	$\left(1+\left|\tilde{U^{d}}\right|\right)\left|G\right|$	$\left|G_{T}\right|+\left(2+2l_{e}+l_{s}\right)\left|G\right|+2l_{e}\left|{\mathbb{Z}}_{p}\right|$	$3l_{e}T_{G}^{e}$	$T_{G_{T}}^{e}+3T_{G}^{e}$	$\left(1+2l_{e}\right)T^{p}+l_{e}T_{G_{T}}^{e}+3l_{e}T_{G}^{e}$	$T_{G_{T}}^{e}$

## Appendix A

**Table A1:** Notations used in OMDAC-ABSC scheme.

Notations	Meaning
$S_{A},S_{U}$	Set of attribute authorities and the set of users.
$N_{A}$	Number of attribute authorities.
uid/aid	Identity of user/authority.
do/du	Identity of data owner (signcryptor)/data user (designcryptor).
$\tilde{U}$	Attribute set of the user.
$\tilde{AA}$	Attribute set of the attribute authority.
$\tilde{U^{d}}/\tilde{U^{s}}$	Decryption/Signing attribute set of the user. $\tilde{U}=\tilde{U^{d}}\cup \tilde{U^{s}}$
$I_{A}^{s}/I_{A}^{e}$	Set of the indexes of the authorities involved in signing/encryption. $I_{A}=I_{A}^{s}\cup I_{A}^{e}$. $N_{A}^{s}=\left|I_{A}^{s}\right|$. $N_{A}^{e}=\left|I_{A}^{e}\right|$.
$H_{1},H_{2},H_{3}$	Collision resistant hash functions.
$R_{s}≔\left(M_{s},\rho_{s}\right)$ $R_{e,j}≔\left(M_{e},\rho_{e}\right)$	Signing and Encryption Predicate
$M_{s}^{i}/M_{e}^{i}$	ith row of $M_{s}^{i}/M_{e}^{i}$.
$M_{s}^{\left(i,k\right)}/M_{e}^{\left(i,k\right)}$	(i,k)th element of $M_{s}^{i}/M_{e}^{i}$.
$\ell_{s}/\ell_{e}$	Number of rows of $M_{s}/M_{e}$ of $R_{s}/R_{e}$.
$n_{s}/n_{e}$	Number of columns of $M_{s}/M_{e}$ of $R_{s}/R_{e}$.
$\ell_{m}$	Maximum value of $\ell_{s}$.
$PP=\left\{\begin{array}{c} g,\theta ,\gamma_{1},\gamma_{2},\;\\ \left\{k_{0},k_{1},\ldots ,k_{l}\right\},\\ \left\{V_{1},V_{2}\ldots ,V_{\ell_{m}}\right\} \end{array}\right\}$	Public parameters.
$s_{uid},d_{uid}$	Secret values chosen by CA for each user with identity uid.
$\varphi_{x},\varphi_{x}^{\prime }$	Attribute version key for attribute x.
$A_{x},A_{x}^{\prime }$	Attribute public key for attribute x.
$PPK_{uid}=\left\{\begin{array}{c} g^{s_{uid}},g^{d_{uid}},\\ {\left\{V_{i}^{s_{uid}}\right\}}_{i\in \left[\ell_{m}\right]} \end{array}\right\}$	Partial public key generated by CA for each user $U_{uid}$.
$PPK_{aid}=\Delta_{aid}$	Partial public key generated by CA for each attribute authority $AA_{aid}$.
$PK_{uid}=\left\{\begin{array}{c} g^{s_{uid}},g^{d_{uid}},g^{1/z_{uid}},\\ \theta^{z_{uid}},g^{z_{uid}},\\ {\left\{V_{i}^{s_{uid}}\right\}}_{i\in \left[\ell_{m}\right]} \end{array}\right\}$	Public key of the user $U_{uid}$.
$SK_{uid}=z_{uid}$	Secret key of the user $U_{uid}$.
$PK_{aid}=\left\{\begin{array}{c} \Delta_{aid},\\ X_{aid},Y_{aid},Z_{aid},\\ {\left\{A_{x}\right\}}_{x\in \tilde{AA_{aid}}} \end{array}\right\}$	Public key of the authority $AA_{aid}$.
$SK_{aid}=\left\{\beta_{aid},\gamma_{aid},{\left\{\varphi_{x}\right\}}_{x\in \tilde{AA_{aid}}}\right\}$	Secret key of the authority $AA_{aid}$.
$PK_{uid,aid}=\left\{\begin{array}{c} PK_{uid,aid}^{1},PK_{uid,aid}^{2},\\ PK_{uid,aid}^{3} \end{array}\right\}$	Public key for each pair of user $U_{uid}$ and authority $AA_{aid}$.
$F_{uid,x}^{s}/F_{uid,x}^{d}$	Signing/Decryption attribute key of $U_{uid}$ for attribute x.
$SK_{uid,aid}^{s}=\left\{\begin{array}{c} K_{uid,aid}^{s},\\ {\left\{F_{uid,x}^{s}\right\}}_{x\in \tilde{U^{s}}\cap \tilde{AA_{aid}}} \end{array}\right\}$	Secret signing key of $U_{uid}$ generated by $AA_{aid}$.
$SK_{uid,aid}^{d}=\left\{\begin{array}{c} K_{uid,aid}^{d},\\ {\left\{F_{uid,x}^{d}\right\}}_{x\in \tilde{U^{s}}\cap \tilde{AA_{aid}}} \end{array}\right\}$	Secret decryption key of $U_{uid}$ generated by $AA_{aid}$.
$PSK_{uid,aid}^{s}=\left\{\begin{array}{c} PS_{uid,aid},PV_{uid},\\ {\left\{PF_{uid,x}^{1},PF_{uid,x}^{2}\right\}}_{x\in \tilde{U^{s}}\cap \tilde{AA_{aid}}},\\ {\left\{V_{i}^{z_{uid}},V_{i}^{s_{uid}z_{uid}}\right\}}_{i\in \left[\ell_{m}\right]} \end{array}\right\}$	Proxy secret key for signing.
$PSK_{uid,aid}^{d}=SK_{uid,aid}^{d}$	Proxy secret key for decryption.
$sUK_{uid,x},dUK_{uid,x}$	Signing and decryption update keys for attribute x.
cUK,sUK	Ciphertext update keys.
$\vec{a}/\vec{b}$	Vectors chosen by fog node for signing protocol.
$s_{uid}^{\prime }$	Secret value randomly chosen by fog node to randomize proxy secret key.
$\left\{r_{1}^{\prime },r_{2}^{\prime },\ldots ,r_{\ell_{e}}^{\prime }\right\},\left\{\lambda_{1}^{\prime },\lambda_{2}^{\prime },\ldots ,\lambda_{\ell_{e}}^{\prime }\right\},w^{\prime }$	Random values chosen by fog node for signcrypion.
$\left\{r_{1},r_{2},\ldots ,r_{\ell_{e}}\right\},\left\{\varepsilon_{2},\ldots ,\varepsilon_{n_{e}}\right\},w$	Random values chosen by data owner for signcrypion.
$thre_{tt}$	Time threshold.
$\left\{\tau_{2},\tau_{3},\ldots ,\tau_{n_{s}}\right\}$	Random values used for verification. $\varpi_{i}=\left(1,\tau_{2},\tau_{3},\ldots ,\tau_{n_{s}}\right)\cdot M_{s}^{i}$
$\left\{\sigma_{1},\sigma_{2},\ldots ,\sigma_{\ell_{e}}\right\}$	Random values chosen by fog node for designcryption.
$CT^{\prime }$	Partial ciphertext computed by fog node in signcryption.
$CT^{p}$	Partial ciphertext computed by fog node in designcryption.
CT	Ciphertext.
